# Scope and limitations of the dual-gold-catalysed hydrophenoxylation of alkynes

**DOI:** 10.3762/bjoc.12.19

**Published:** 2016-02-01

**Authors:** Adrián Gómez-Suárez, Yoshihiro Oonishi, Anthony R Martin, Steven P Nolan

**Affiliations:** 1 EaStCHEM School of Chemistry, University of St. Andrews, North Haugh, St. Andrews, Fife, KY16 9ST, U.K; 2 Faculty of Pharmaceutical Sciences, Hokkaido University, Sapporo 060-0812, Japan; 3 Institut de Chimie de Nice, UMR 7272, Université de Nice Sophia Antipolis, CNRS, Parc Valrose, 06108 Nice cedex 2, France; 4 Chemistry Department, College of Science, King Saud University, P.O. Box 2455, Riyadh 11451, Saudi Arabia; 5 Universiteit Gent, Department of Inorganic and Physical Chemistry, Krijgslaan 281, S-3, B-9000 Ghent, Belgium

**Keywords:** cooperative catalysis, gold catalysis, hydrophenoxylation, N-heterocyclic carbene, vinyl ethers

## Abstract

Due to the synthetic advantages presented by the dual-gold-catalysed hydrophenoxylation of alkynes, a thorough study of this reaction was carried out in order to fully define the scope and limitations of the methodology. The protocol tolerates a wide range of functional groups, such as nitriles, ketones, esters, aldehydes, ketals, naphthyls, allyls or polyphenols, in a milder and more efficient manner than the previously reported methodologies. We have also identified that while we are able to use highly steric hindered phenols, small changes on the steric bulk of the alkynes have a dramatic effect on the reactivity. More importantly, we have observed that the use of substrates that facilitate the formation of diaurated species such as *gem*-diaurated or σ,π-digold–acetylide species, hinder the catalytic activity. Moreover, we have identified that the use of directing groups in unsymmetrical alkynes can help to achieve high regioselectivity in the hydrophenoxylation.

## Introduction

**Scheme 1:**
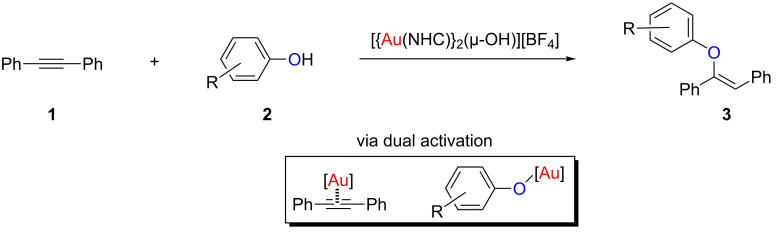
Dual-gold-catalysed hydrophenoxylation of alkynes.

During the last 30 years, N-heterocyclic carbenes (NHCs) have evolved from mere curiosities to one of the most powerful tools within the synthetic chemist’s arsenal [1]. Due to their unique steric and electronic properties [2–6], NHCs are nowadays used in a myriad of chemical processes, for example as catalysts in organocatalysed reactions [7–9], or as ligands in transition metal and main-group chemistry [10–13]. We have been interested in exploring the use of NHC ligands in transition metal complexes for the development of highly active and well-defined catalysts. One of our main interests during the last decade has been to study the use of gold–NHC species as powerful catalysts for the construction of valuable organic molecules via the construction of C–C or C–O bonds [14–23]. In this regard, we have recently reported the use of [{Au(NHC)}_2_(µ-OH)][BF_4_] species as dual-activation catalysts for the hydrophenoxylation of alkynes [24–25]. This transformation proceeds through the interaction of the gold centres with both the phenol and the alkyne motifs, thus generating a synergistic effect that produces unique catalytic activity (Scheme 1) [24–25]. In addition, this reaction is highly stereoselective and only the *Z*-isomer was observed, regardless of the substrate used.

This new, dual-catalysed methodology presents several advantages over previously reported protocols [26–27], such as milder reaction conditions or the use of lower catalyst loading. Therefore, encouraged by our preliminary studies [24], and the potential applications of the vinyl ether derivatives **3** as synthetic building blocks [28], we decided to further explore the scope and limitations of the dual-gold-catalysed hydrophenoxylation of alkynes. With this study, we sought to answer the following questions: what is the functional group tolerance of our transformation? How do steric and electronic factors affect both reaction partners? Can regioselectivity be achieved for the addition of phenols to unsymmetrical alkynes?

## Results and Discussion

### Functional group tolerance

**Scheme 2:**
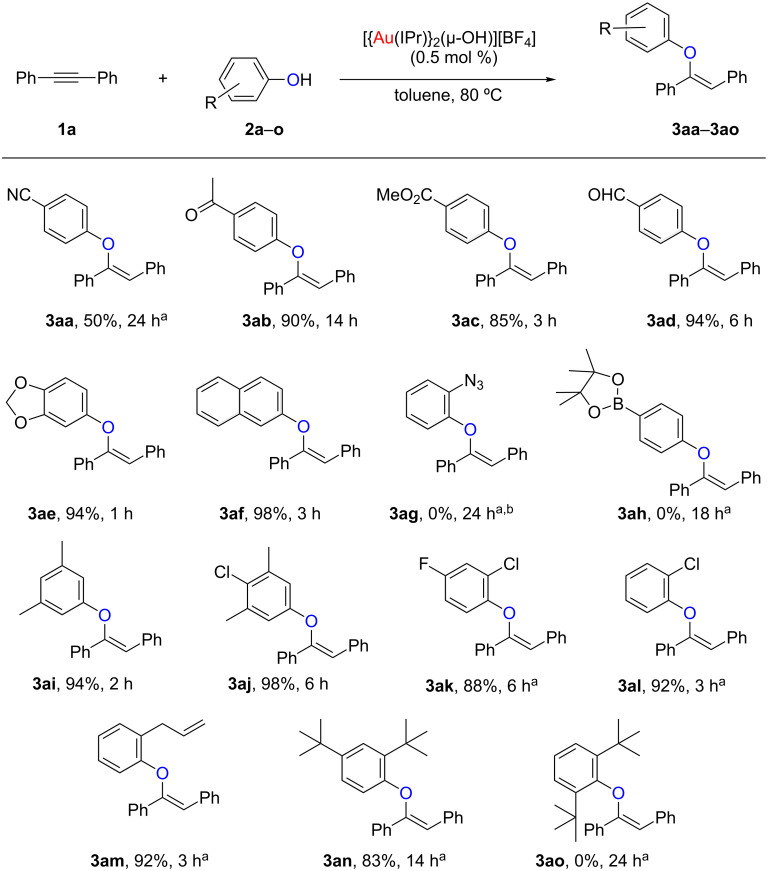
Exploring the functional group tolerance. Reaction conditions: **1a** (0.50 mmol, 1 equiv), **2a**–**o** (0.55 mmol, 1.1 equiv), [Au] (2.5 µmol, 0.5 mol %), toluene (1 mL), 80 °C. Isolated yields, average of two runs. ^a^[Au] (5 µmol, 1 mol %), 110 °C. ^b^GC analysis showed a complex mixture.

We commenced our studies by assessing the functional group tolerance of the methodology (Scheme 2). With that aim we reacted diphenylacetylene (**1a**) with several phenols, **2a**–**o**, in toluene, using 0.5 mol % of [{Au(IPr)}_2_(µ-OH)][BF_4_] (IPr = 1,3-bis(2,6-diisopropylphenyl)imidazol-2-ylidene) as the catalyst. Functional groups such as nitriles (**3aa**), ketones (**3ab**), esters (**3ac**), aldehydes (**3ad**), acetals (**3ae**) and naphthyls (**3af**) were well tolerated and the corresponding vinyl ethers were obtained in moderate to high yields (50–98%). On the contrary, the use of azide (**3ag**) or boronic ester (**3ah**) functionalities did not afford the desired products [29]. The latter case can be explained due to formation of the corresponding *gem*-diaurated aryl species, by reaction of catalyst [{Au(IPr)}_2_(µ-OH)][BF_4_] with the boronic ester [30], thus inhibiting the catalytic activity. The electronic properties of the phenol were also examined. If the electron density on the phenol is decreased, its nucleophilicity decreases and an increase in catalyst loading is needed in order to maintain relatively short reaction times (**3ai** and **3al**) [31]. The steric hindrance on the phenol was studied next. Increasing it, with either allyl (**3am**) or *tert*-butyl (**3an**) groups on the *ortho*-position also required 1 mol % of catalyst, but the reaction proceeded smoothly. Encouraged by these and past results with highly hindered substrates, we attempted the addition of 2,6-di-*tert*-butylphenol (**2ao**). Unfortunately, this reaction did not proceed at all and only starting materials were observed by GC–FID. These results, in combination with our previous studies [24], where *para*-MeO, -CF_3_, -NO_2_, -Cl or -F substituents were also tolerated, support the robustness of our protocol, as it is highly efficient in the presence of a wide range of functional groups.

### Polyphenols as nucleophiles

**Scheme 3:**
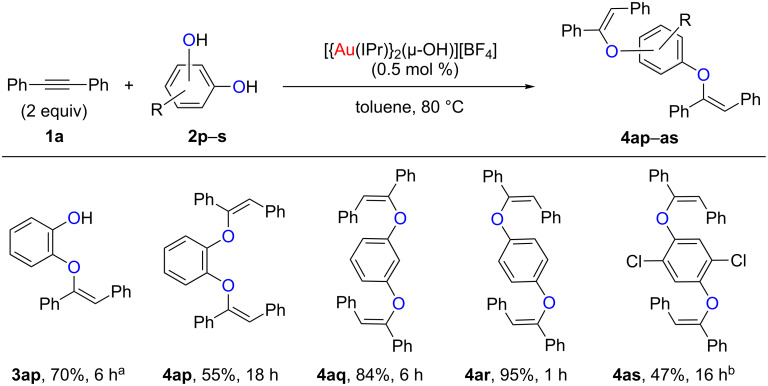
Hydrophenoxylation using polyphenols. Reaction conditions: **1a** (1 mmol, 2 equiv), **2p**–**s** (0.50 mmol, 1 equiv), [Au] (2.5 µmol, 0.5 mol %), toluene (1 mL), 80 °C. Isolated yields, average of two runs. ^a^1 equiv **1a**. ^b^[Au] (5 µmol, 1 mol %), 110 °C.

Intrigued by the versatility of our protocol, we explored the reaction using polyphenols as substrates (Scheme 3). Interestingly, we were able to selectively functionalize one (**3ap**) or both (**4ap**) hydroxy groups of catechol (**2p**) by using one or two equiv of **1a**, respectively. Unfortunately, for the other polyphenol derivatives only the difunctionalised products could be observed (**4aq**–**4as**). We hypothesize that this difference in reactivity may be due to the steric hindrance around the monofunctionalised product **3ap**, allowing for a significant rate difference in the reactions of **3ap** and **4ap**. In other cases, the greater separation between the hydroxy functional groups likely reduces this difference. To put these results in context, the previously reported methodology required 10 mol % of AuCl_3_ and 96 h in order to obtain similar yields for **4aq** [26]. Moreover, only 7% of **4ap** could be isolated after 168 h [26].

### Electronic and steric effects on the alkyne

**Scheme 4:**
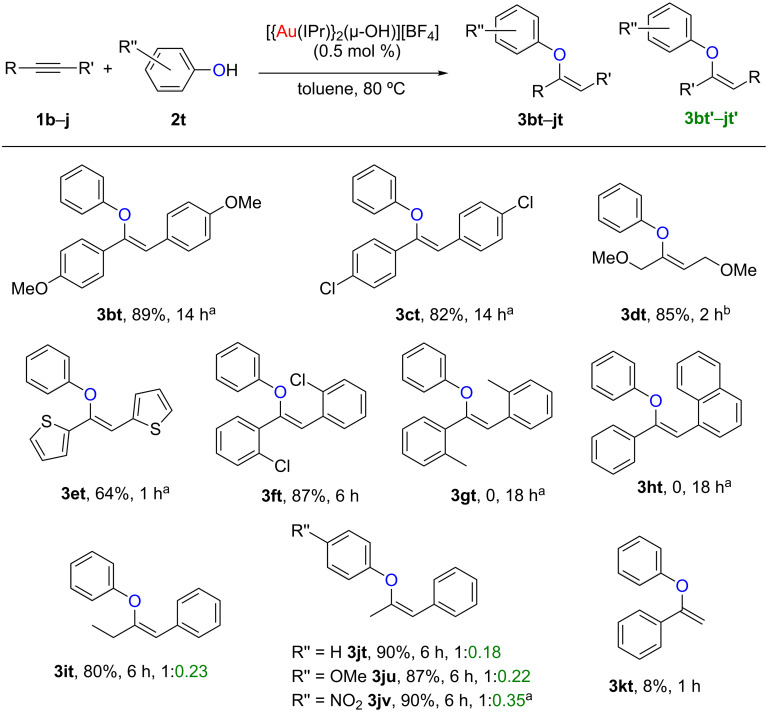
Hydrophenoxylation of (un)symmetrical alkynes. Reaction conditions: **1b**–**k** (0.50 mmol, 1 equiv), **2t** (0.55 mmol, 1.1 equiv), [Au] (2.5 µmol, 0.5 mol %), toluene (1 mL), 80 °C. Isolated yields, average of two runs. Ratio determined by ^1^H NMR. ^a^[Au] (5 µmol, 1 mol %), 110 °C. ^b^**1d** (1 mmol, 2 equiv), **2t** (0.50 mmol, 1 equiv).

Next, we sought to assess how the electron density on the alkyne affected the reaction. Therefore, both electron-rich (**1b**, **1e**) and electron-poor (**1c**) alkynes were tested using phenol **2t** as nucleophile. The desired vinyl ethers, **3bt**, **3ct** and **3et**, were obtained in moderate to good yields (Scheme 4). In order to expand the alkyne scope, propargylic ethers were also tested. In this case, 2 equiv of the alkyne were needed to obtain good yields of the desired product, **3dt**. Next, we targeted the steric hindrance tolerated around the alkyne. With this aim in mind we decided to use diaryl-substituted alkynes bearing *ortho*-substituents due to synthetic reasons. The use of the *ortho*-chlorophenyl derivative **1f** afforded the desired vinyl ether **3ft** in good yields, while changing the chlorides for methyl groups resulted in no reaction, even when using harsher reaction conditions (**3gt**, Scheme 4). One more highly hindered substrate, **1h**, bearing a sterically demanding naphthyl group on one side, and a phenyl on the other was studied. No reaction was observed even after increasing the catalyst loading and temperature (**3ht**, Scheme 4). Next, we focused our attention on whether the chain length on unsymmetrical alkyl–aryl alkynes, or the electron density on the phenol could affect the regioselectivity of the reaction. The use of 1-phenylbut-1-yne (**1i**) afforded the desired vinyl ethers **3it** and **3it'** in a 1:0.23 ratio and in good yields. This selectivity is slightly lower than our previously reported hydrophenoxylation of phenylpropyne (**1j**, 1:0.18) [24]. We also studied if the electron density on the phenol could alter the selectivity. Addition of *p*-methoxyphenol (**2u**) to **1j** afforded a 1:0.22 ratio between the two expected products, **3ju** and **3ju'**. This selectivity is slightly diminished compared to the addition of phenol to **1j** (1:0.18), but better than the addition of *p*-nitrophenol (**2v**, 1:0.35), both reported in our previous study (**3jt** and **3jv**, Scheme 4) [24]. Finally, we tested whether terminal alkynes were tolerated by our methodology. Phenylacetylene (**1k**) was reacted with phenol (**2t**) under our standard reaction conditions, 0.5 mol % [{Au(IPr)}_2_(µ-OH)][BF_4_] as catalyst, in toluene at 80 °C for 1 h. However, GC–FID analysis of the reaction mixture revealed only traces of product. A more careful study of the crude reaction mixture by ^1^H NMR spectroscopy, revealed 8% conversion to the desired vinyl ether **3kt**, as well as 10% of acetophenone, the corresponding hydration product. Furthermore, the formation of a new gold species, which was characterised as the previously reported σ,π-digold–acetylide species could also be observed. This diaurated species can be formed by reaction of [{Au(IPr)}_2_(µ-OH)][BF_4_] with phenylacetylene [30]. As in the case of the formation of a *gem*-diaurated species when boronic ester **3h** was used, it appears that in situ formation of highly stable diaurated species, inhibits catalytic activity.

These results suggest that while both electron-rich/poor alkynes are tolerated by our methodology, the tolerated steric hindrance is very low, in contrast to the results observed for the nucleophile. In addition, in the case of unsymmetrical alkynes, the use of electron-rich alkynes appears to favour a better selectivity than their electron-poor counterparts.

### Enhancing regioselectivity using directing groups

**Scheme 5:**
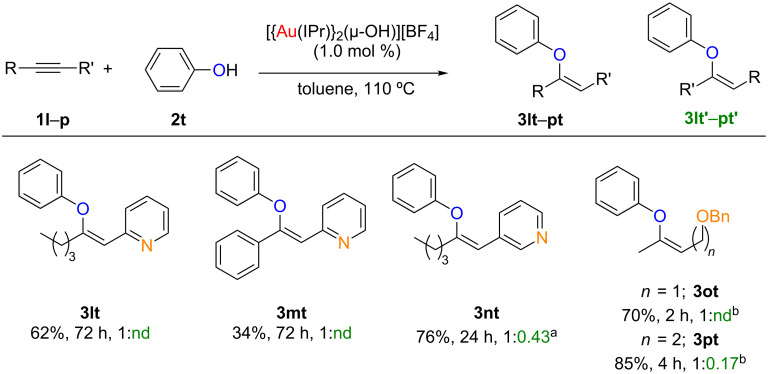
Regioselective hydrophenoxylation of unsymmetrical alkynes. Reaction conditions: **1l**–**p** (1 equiv), **2a** (1.1 equiv), [Au] (1 mol %), toluene (1 mL), 110 °C. Isolated yields, average of two runs. Ratio determined by ^1^H NMR. nd = not detected. ^a^[Au] (3 mol %). ^b^[Au] (0.5 mol %), **1o** (2 equiv.)

During our preliminary study on the hydrophenoxylation of unsymmetrical alkynes [24], we observed that the use of alkynes bearing a 2-pyridinyl moiety afforded only one of the possible vinyl ether derivatives (**3lt** and **3mt**, Scheme 5). We hypothesised that this directing effect could be a combination of the charge polarization on the alkyne and an interaction between the pyridine nitrogen and one of the gold centres. To test our hypothesis, we synthesized an alkyne bearing a 3-pyridinyl (**1n**) substituent. In order to reduce the reaction time the catalyst loading was increased to 3 mol %. Interestingly, the selectivity dropped dramatically and a 1:0.43 ratio of products was observed (**3nt**, Scheme 5). In order to further test our hypothesis, the polarization of the alkyne was next reduced, but the potential chelating heteroatom was retained. The hydrophenoxylation of the unsymmetrical propargylic ether **1o** afforded, with complete regioselectivity, the corresponding vinyl ether **3ot** [32]. As expected, when homopropargylic ether **1p** was used diminished selectivity was observed. Thus supporting our hypothesis that regioselectivity can be obtained by assistance of a directing group, either by polarization of the alkyne or by a chelating effect [33].

## Conclusion

We have explored an extended range of reactions in order to fully define the scope and limitations of the digold-mediated hydrophenoxylation of alkynes. With these additions we have performed the gold-catalysed hydroalkoxylation of alkynes on ca. 50 substrates, thus demonstrating the robustness and synthetic versatility of the methodology. The protocol tolerates a wide range of functional groups, such as nitriles, ketones, esters, aldehydes, acetals, naphthyls or allyls. Furthermore, we have successfully used polyphenols in a milder and more efficient manner than the previously reported methodologies. We have also identified that while we are able to use highly steric hindered phenols, small changes on the steric bulk of the alkynes have a dramatic effect on reactivity. More importantly, we have observed that the use of substrates that facilitate the formation of diaurated species such as *gem*-diaurated or σ,π-digold–acetylide species, hinder the catalytic activity. We have identified that the use of directing groups in unsymmetrical alkynes can help achieve regioselectivity in the hydrophenoxylation reaction. With these studies, we have sought to contribute to a better understanding of the interactions involved in dual-gold-catalysed reactions, and anticipate that this information will help to design more efficient dual-activation protocols. We are currently studying the use of digold–hydroxide species in other dual-gold-catalysed reactions and these results will be published in due course.

## Supporting Information

File 1 Experimental procedures and characterisation data for all the compounds.
