# Supplementary material for: Scope and limitations of the dual-gold-catalysed hydrophenoxylation of alkynes
Source: Beilstein J Org Chem. 2016 Feb 1;12:172–8. doi: 10.3762/bjoc.12.19 (PMC4778530; doi:10.3762/bjoc.12.19)
Supplement: File 1 — Experimental procedures and characterisation data for all the compounds. [file Beilstein_J_Org_Chem-12-172-s001.pdf]

Supporting Information

for

Scope and limitations of the dual-gold-catalysed hydrophenoxylation of alkynes

Adrián Gómez-Suárez[1], Yoshihiro Oonishi[1,2], Anthony R. Martin[1,3] and Steven P. Nolan*[4,5]

Address: [1]EaStCHEM School of Chemistry, University of St. Andrews, North Haugh, St. Andrews, Fife, KY16 9ST, U.K, [2]Faculty of Pharmaceutical Sciences, Hokkaido University, Sapporo 060-0812, Japan, [3]Institut de Chimie de Nice, UMR 7272, Université de Nice Sophia Antipolis, CNRS, Parc Valrose, 06108 Nice cedex 2, France, [4]Chemistry Department, College of Science, King Saud University, P.O. Box 2455, Riyadh 11451, Saudi Arabia and [5]Universiteit Gent, Department of Inorganic and Physical Chemistry, Krijgslaan 281, S-3, B-9000 Ghent, Belgium.

Email: Steven P. Nolan - stevenpnolan@gmail.com

*Corresponding author

Experimental procedures and characterisation data for all the compounds

Table of Contents

1. General considerations S3

2. Synthesis & characterization of vinyl ether derivatives (6) S3

3. References S11

4. NMR Spectra S12

1. General considerations

Unless otherwise stated, all solvents and reagents were used as purchased and all reactions were performed under air. Deuterated solvents (CD_2Cl_2, $CDCl_3$) were filtered through basic alumina in order to remove traces of HCl. NMR spectra were recorded on 500, 400 and 300 MHz spectrometers at room temperature in CD_2Cl_2 or $CDCl_3$. Chemical shifts (δ) are reported in ppm, relative to the solvent residual peak CD_2Cl_2 (5.32 ppm for ^{1}H and 54.00 ppm for ^{13}C) and $CDCl_3$ (7.26 ppm for ^{1}H and 77.16 ppm for ^{13}C). Data for ^{1}H NMR are reported as follows: chemical shift, multiplicity (s = singlet, d = doublet, t = triplet, br = broad signal, m = multiplet), coupling constants (*J*) in Hz and integration. Flash chromatography was performed on silica gel 60 Å pore diameter and 40–63 µm particle size. Elemental analysis was carried out by the analytical services of London Metropolitan University. High-resolution mass spectrometry was performed by the EPSRC National Mass Spectrometry Service Centre (NMSSC) (Grove Building Extn., Swansea University, Singleton Park, Swansea, SA2 8PP, U.K.). [{Au(IPr)}$_2$(µ-OH)][BF$_4$] was synthesized following the reported methodologies [1]:

2. Synthesis & characterization of vinyl ether derivatives (6)

General procedure

As described in reference [2], {Au(NHC)}$_2$(µ-OH)][BF$_4$] (0.5–1.0 mol %) was added to a solution of alkyne (0.5 mmol) and phenol (0.55 mmol, 1.1 equiv) in toluene (1 mL). The reaction mixture was stirred at 80 or 110 °C. After the reaction was completed, the solvent was concentrated in vacuum. The residue was purified by flash column chromatography on silica gel to give the corresponding product.

(*Z*)-4-((1,2-Diphenylvinyl)oxy)benzonitrile (3aa)

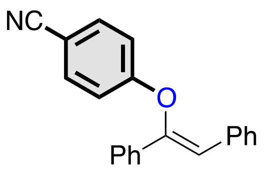

According to the general procedure for hydrophenoxylation, a crude product, which was prepared from diphenylacetylene (**1a**) (89.0 mg, 0.50 mmol), 4-hydroxybenzonitrile (**2a**) (65.5 mg, 0.55 mmol) and [{Au(IPr)}$_2$(µ-OH)][BF$_4$] (6.5 mg, 5 µmol, 1.0 mol %) in toluene (1 mL) at 110 °C for 24 h, was purified by column chromatography on silica gel (*n*-hexane/EtOAc = 95/5) to give **6aa** (74 mg, 50%, average of two runs) as a white solid.

^{1}H NMR ($CDCl_3$, 500 MHz): δ 7.58-7.55 (m, 4H), 7.54-7.51 (m, 2H), 7.38-7.28 (m, 5H), 7.25-7.22 (m, 1H), 7.11-7.07 (m, 2H), 6.75 (s, 1H). **^{13}C{^{1}H} NMR** ($CDCl_3$, 101 MHz): δ 159.94, 148.60, 135.02, 134.36, 134.05, 129.11, 129.03, 128.99, 128.78, 128.03, 125.79, 118.95, 117.43, 117.04, 105.72; **HRMS** (NIS) calcd for $C_{21}H_{16}NO$ [(M+H)+] 298.1226, found 298.1233.

(*Z*)-1-(4-((1,2-Diphenylvinyl)oxy)phenyl)ethanone (3ab)

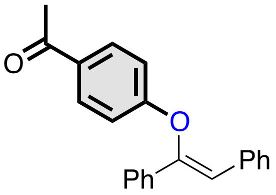

According to the general procedure for hydrophenoxylation, a crude product, which was prepared from diphenylacetylene (**1a**) (89.0 mg, 0.50 mmol), 4-hydroxyacetophenone (**2b**) (74.8 mg, 0.55 mmol) and [{Au(IPr)}$_2$(µ-OH)][BF$_4$] (3.2 mg, 2.5 µmol, 0.5 mol %) in toluene (1 mL) at 80 °C for 14 h, was purified by column chromatography on silica gel (*n*-hexane/EtOAc = 95/5) to give **3ab** (140 mg, 90%, average of two runs) as a white solid.

^{1}H NMR ($CDCl_3$, 500 MHz): δ 7.88-7.86 (m, 2H), 7.61-7.58 (m, 4H), 7.36-7.28 (m, 5H), 7.24-7.21 (m, 1H), 7.09-7.06 (m, 2H), 6.74 (s, 1H), 2.51 (s, 3H).; **^{13}C{^{1}H} NMR** ($CDCl_3$, 101 MHz): δ 196.75, 160.51, 149.03, 135.46, 134.34, 131.69, 130.85, 129.11, 128.89, 128.84, 128.73, 127.83, 125.89, 117.18, 116.12, 26.49; **HRMS** (NIS) calcd for $C_{22}H_{19}O_2$ [$(M+H)^+$] 315.1380, found 315.1385.

(*Z*)-Methyl 4-[(1,2-diphenylvinyl)oxy]benzoate (3ac)

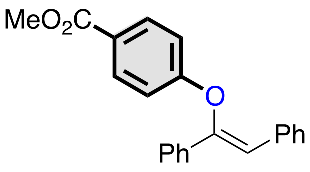

According to the general procedure for hydrophenoxylation, a crude product, which was prepared from diphenylacetylene (**1a**) (89.0 mg, 0.50 mmol), methyl 4-hydroxybenzoate (**2c**) (83.7 mg, 0.55 mmol) and [{Au(IPr)}$_2$(μ-OH)][BF_4] (3.2 mg, 2.5 μmol, 0.5 mol %) in toluene (1 mL) at 80 °C for 3 h, was purified by column chromatography on silica gel (*n*-hexane/EtOAc = 9/1) to give **3ac** (140 mg, 85%, average of two runs) as a white solid.

^{1}H NMR ($CDCl_3$, 400 MHz) δ 7.97-7.88 (m, 2H), 7.64-7.53 (m, 4H), 7.39-7.17 (m, 6H), 7.09-6.99 (m, 2H), 6.72 (s, 1H), 3.85 (s, 3H); **^{13}C{^{1}H} NMR** ($CDCl_3$, 101 MHz) δ 166.7, 160.4, 149.1, 135.5, 134.4, 131.9, 129.1, 128.9, 128.8, 128.7, 127.8, 125.9, 124.2, 117.1, 116.1, 52.1; **HRMS** (NIS) calcd for $C_{22}H_{19}O_3$ [$(M+H)^+$] 331.1329, found 331.1327.

(*Z*)-4-((1,2-Diphenylvinyl)oxy)benzaldehyde (3ad)

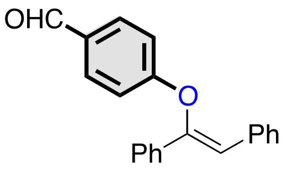

According to the general procedure for hydrophenoxylation, a crude product, which was prepared from diphenylacetylene (**1a**) (89.0 mg, 0.50 mmol), phenol (**2d**) (67.0 mg, 0.50 mmol) and [{Au(IPr)}$_2$(μ-OH)][BF_4] (3.2 mg, 2.5 μmol, 0.5 mol %) in toluene (1 mL) at 80 °C for 6 h, was purified by column chromatography on silica gel (CH_2Cl_2), to give **3ad** (142 mg, 94%, average of two runs) as a colourless solid.

^{1}H NMR (CD_2Cl_2, 300 MHz) δ 9.83 (s, 1H), 7.78-7.76 (m, 2H), 7.62-7.59 (m, 4H), 7.36-7.15 (m, 8H), 6.80 (s, 1H); **^{13}C{^{1}H} NMR** ($CDCl_3$, 75 MHz) δ 151.6, 150.4, 136.5, 135.3, 129.4, 129.1, 129.0, 127.9, 126.6, 117.6, 117.2; **HRMS** (APCI) calcd for $C_{21}H_{17}O_2$ [$(M+H)^+$] 301.1223, found 301.1222.

(*Z*)-5-((1,2-Diphenylvinyl)oxy)benzo[*d*][1,3]dioxole (3ae)

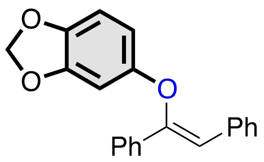

According to the general procedure for hydrophenoxylation, a crude product, which was prepared from diphenylacetylene (**1a**) (89.0 mg, 0.50 mmol), sesamol (**2e**) (70.0 mg, 0.50 mmol) and [Au(IPr)]$_2$(μ-OH)][BF_4] (3.2 mg, 2.5 μmol, 0.5 mol %) in toluene (1 mL) at 80 °C for 1 h, was purified by column chromatography on silica gel (*n*-hexane/EtOAc = 95/5) to give **3ae** (150 mg, 94%, average of two runs) as a white solid.

^{1}H NMR (400 MHz; $CDCl_3$): δ 7.69-7.67 (m, 2H), 7.62-7.60 (m, 2H), 7.37-7.23 (m, 6H), 6.65 (d, *J* = 8.5 Hz, 1H + d, *J* = 2.5 Hz, 1H + s, 1H), 6.49 (dd, *J* = 8.5, 2.5 Hz, 1H), 5.88 (s, 2H). **^{13}C{^{1}H} NMR** (101 MHz, $CDCl_3$): δ 151.46, 150.08, 148.47, 142.66, 136.07, 134.85, 129.03, 128.66, 128.52, 127.52, 126.23, 116.82, 108.30, 108.23, 101.40, 99.18. **HRMS** (APCI) calcd. for $C_{21}H_{17}O_3$ [$(M+H)^+$] 317.1172, found 317.1170.

(*Z*)-2-((1,2-Diphenylvinyl)oxy)naphthalene (3af)

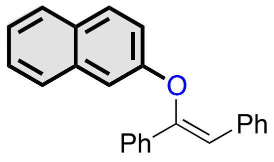

According to the general procedure for hydrophenoxylation, a crude product, which was prepared from diphenylacetylene (**1a**) (89.0 mg, 0.50 mmol), naphthol (**2f**) (79.3 mg, 0.55 mmol) and [{Au(IPr)}$_2$(μ-OH)][BF_4] (3.2 mg, 2.5 μmol, 0.5 mol %) in toluene (1 mL) at 80 °C for 3 h, was purified by column chromatography on silica gel (*n*-hexane/EtOAc = 95/5) to give **3af** (158 mg, 98%, average of two runs) as a white solid.

^{1}H NMR ($CDCl_3$, 300 MHz) δ 7.82-7.72 (m, 2H), 7.71-7.57 (m, 5H), 7.43-7.14 (m, 10H), 6.75 (s, 1H); **^{13}C{^{1}H} NMR** ($CDCl_3$, 75 MHz) δ 154.3, 149.7, 135.9, 134.8, 134.5, 130.0, 129.7, 129.1, 128.7, 128.7, 128.6, 127.8, 127.6, 127.1, 126.5, 126.1, 124.3, 118.3, 117.1, 111.1; **HRMS** (APCI) calcd for $C_{24}H_{19}O$ [$(M+H)^+$] 323.1430, found 323.1430.

(*Z*)-[1-(3,5-Dimethylphenoxy)ethene-1,2-diyl]dibenzene (3ai)

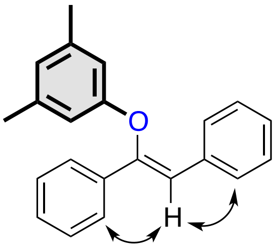

NOESY

According to the general procedure for hydrophenoxylation, a crude product, which was prepared from diphenylacetylene (**1a**) (89.0 mg, 0.50 mmol), 3,5-dimethylphenol (**2i**) (67.0 mg, 0.55 mmol) and [{Au(IPr)}$_2$(μ-OH)][BF_4] (3.2 mg, 2.5 μmol, 0.5 mol %) in toluene (1 mL) at 80 °C for 2 h, was purified by column chromatography on silica gel (*n*-hexane/EtOAc = 95/5) to give **3ai** (142 mg, 94%, average of two runs) as a white solid.

^{1}H NMR ($CDCl_3$, 400 MHz) δ 7.68-7.62 (m, 2H), 7.62-7.57 (m, 2H), 7.37-7.15 (m, 6H), 6.65 (brs, 2H), 6.64 (s, 1H), 6.59 (brs, 1H), 2.21 (s, 6H); **^{13}C{^{1}H} NMR** ($CDCl_3$, 101 MHz) δ 156.4, 149.8, 139.5, 139.5, 136.3, 135.0, 129.1, 128.6, 128.4, 127.4, 126.1, 124.0, 116.7, 114.1, 21.5; **HRMS** (APCI) calcd for $C_{22}H_{20}O$ [M^+] 300.1509, found 300.1507.

(*Z*)-(1-(4-Chloro-3,5-dimethylphenoxy)ethene-1,2-diyl)dibenzene (3aj)

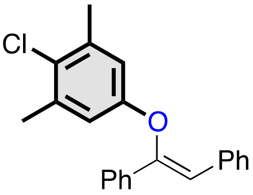

According to the general procedure for hydrophenoxylation, a crude product, which was prepared from alkyne **1a** (89.0 mg, 0.50 mmol), phenol (**2j**) (86.0 mg, 0.50 mmol) and [{Au(IPr)}$_2$(μ-OH)][BF_4] (3.2 mg, 2.5 μmol, 0.5 mol %) in toluene (1 mL) at 80 °C for 6 h, was purified by column chromatography on silica gel (pentane/EtOAc = 95/5), to give **3aj** (164 mg, 98%, average of two runs) as a colourless solid.

^{1}H NMR ($CDCl_3$, 300 MHz) δ 7.64 (ddd, *J* = 14.4, 7.7, 1.5 Hz, 4H), 7.38-7.22 (m, 6H), 6.80 (s, 2H), 6.69 (s, 1H), 2.30 (s, 6H); **^{13}C{^{1}H} NMR** ($CDCl_3$, 75 MHz) δ 154.2, 149.5, 137.6, 135.9, 134.8, 129.1, 128.7, 128.7, 128.6, 127.6, 126.1, 117.0, 116.2, 21.1; **HRMS** (APCI) calcd for $C_{22}H_{20}OCl$ [$(M+H)^+$] 335.1197, found 335.1195.

(*Z*)-[1-(2-Chloro-4-fluorophenoxy)ethene-1,2-diyl]dibenzene (3ak)

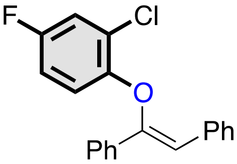

According to the general procedure for hydrophenoxylation, a crude product, which was prepared from diphenylacetylene (**1a**) (89.0 mg, 0.50 mmol), 2-chloro-4-fluorophenol (**2k**) (81.0 mg, 0.55 mmol) and [{Au(IPr)}$_2$(μ-OH)][BF_4] (6.5 mg, 5 μmol, 1.0 mol %) in toluene (1 mL) at 110 °C for 6 h, was purified by column chromatography on silica gel (*n*-hexane/EtOAc = 95/5) to give **3ak** (143 mg, 88%, average of two runs) as a white solid whose NMR data were consistent to those reported in the literature [3].

^{1}H NMR ($CDCl_3$, 300 MHz) δ 7.70-7.61 (m, 2H), 7.61-7.54 (m, 2H), 7.39-7.13 (m, 7H), 6.77 (dd, *J* = 9.1, 5.0 Hz, 1H), 6.73-6.64 (m, 2H); **^{13}C{^{1}H} NMR** ($CDCl_3$, 75 MHz) δ 157.3 (d, J_{C-F} = 243 Hz), 149.7, 148.1 (d, J_{C-F} = 2.8 Hz), 135.2, 134.4, 129.0, 128.88, 128.87, 128.7, 127.8, 126.0, 123.4 (d, J_{C-F} = 11 Hz), 117.8 (d, J_{C-F} = 26 Hz), 117.2, 116.6 (d, J_{C-F} = 8.6 Hz), 114.5 (d, J_{C-F} = 23 Hz); ^{19}F NMR ($CDCl_3$, 282 MHz) δ -120.3.

(*Z*)-[1-(2-Chlorophenoxy)ethene-1,2-diyl]dibenzene (3al)

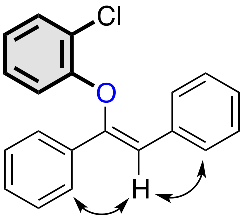

NOESY

According to the general procedure for hydrophenoxylation, a crude product, which was prepared from diphenylacetylene (**1a**) (89.0 mg, 0.50 mmol), 2-chlorophenol (**2l**) (70.7 mg, 0.55 mmol) and [{Au(IPr)}$_2$(μ-OH)][BF_4] (6.5 mg, 5 μmol, 1.0 mol %) in toluene (1 mL) at 110 °C for 3 h, was purified by column chromatography on silica gel (*n*-hexane/EtOAc = 95/5) to give **3al** (141 mg, 92%, average of two runs) as a colourless liquid whose NMR data were consistent to those reported in the literature [3].

^{1}H NMR ($CDCl_3$, 300 MHz) δ 7.72-7.65 (m, 2H), 7.65-7.57 (m, 2H), 7.43 (dd, *J* = 7.8, 1.7 Hz, 1H), 7.39-7.18 (m, 6H), 6.99 (dt, *J* = 7.7, 1.7 Hz, 1H), 6.93-6.79 (m, 2H), 6.72 (s, 1H); **^{13}C{^{1}H} NMR** ($CDCl_3$, 75 MHz) δ 151.7, 149.5, 135.4, 134.5, 130.6, 129.1, 128.8, 128.8, 128.7, 127.8, 127.7, 125.9, 123.0, 122.9, 117.1, 116.3.

(*Z*)-[1-(2-Allylphenoxy)ethene-1,2-diyl]dibenzene (3am)

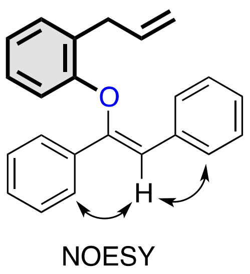

According to the general procedure for hydrophenoxylation, a crude product, which was prepared from diphenylacetylene (**1a**) (89.0 mg, 0.50 mmol), 2-allylphenol (**2m**) (74.0 mg, 0.55 mmol) and [{Au(IPr)}$_2$(μ-OH)][BF_4] (6.5 mg, 5 μmol, 1.0 mol %) in toluene (1 mL) at 110 °C for 3 h, was purified by column chromatography on silica gel (*n*-hexane/EtOAc = 95/5) to give **3am** (144 mg, 92%, average of two runs) as a white solid.

^{1}H NMR ($CDCl_3$, 300 MHz): δ 7.66-7.59 (m, 2H), 7.58-7.51 (m, 2H), 7.39-7.15 (m, 7H), 7.02-6.84 (m, 2H), 6.73 (dd, *J* = 7.8, 1.6 Hz, 1H), 6.67 (s, 1H), 6.25-6.06 (m, 1H), 5.25-5.09 (m, 2H), 3.69 (ddd, *J* = 6.5, 1.6, 1.6 Hz, 2H); **^{13}C{^{1}H} NMR** ($CDCl_3$, 101 MHz) δ 153.8, 149.7, 137.0, 136.1, 134.9, 130.6, 129.0, 128.7, 128.6, 128.5, 128.4, 127.51, 127.45, 126.0, 122.0, 116.9, 116.1, 114.5, 34.3; **HRMS** (APCI) calcd for $C_{23}H_{21}O$ [$(M+H)^+$] 313.1587, found 313.1588.

(*Z*)-(1-(2,4-Di-*tert*-butylphenoxy)ethene-1,2-diyl)dibenzene (3an)

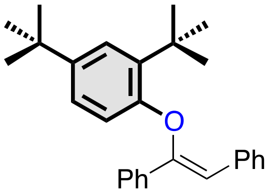

According to the general procedure for hydrophenoxylation, a crude product, which was prepared from diphenylacetylene (**1a**) (89.0 mg, 0.50 mmol), 2,4-di-*tert*-butylphenol (**2o**) (103.2 mg, 0.50 mmol) and [{Au(IPr)}$_2$(μ-OH)][BF_4] (6.5 mg, 5 μmol, 1.0 mol %) in toluene (1 mL) at 80 °C for 14 h, was purified by column chromatography on silica gel (*n*-hexane/EtOAc = 95/5) to give **3an** (160 mg, 83%, average of two runs) as an off-white solid.

^{1}H NMR ($CDCl_3$, 300 MHz): δ 7.62 - 7.58 (m, 2H), 7.57 - 7.53 (m, 2H), 7.40 (d, *J* = 2.5 Hz, 1H), 7.35 - 7.21 (m, 5H), 7.20 - 7.15 (m, 1H), 6.93 (dd, *J* = 8.5, 2.5 Hz, 1H), 6.69 (d, *J* = 8.7 Hz, 1H), 6.67 (s, 1H), 1.58 (s, 9H), 1.26 (s, 9H). **^{13}C{^{1}H} NMR** (101 MHz, $CDCl_3$): δ 152.77, 149.60, 143.77, 136.60, 136.35, 134.95, 129.04, 128.61, 128.42, 128.30, 127.35, 126.43, 124.51, 123.79, 117.49, 114.69, 35.40, 34.44, 31.72, 30.63. **HRMS** (APCI) calcd for $C_{28}H_{33}O$ [$(M+H)^+$] 385.2526, found 385.2519.

(*Z*)-2-((1,2-Diphenylvinyl)oxy)phenol (3ap)

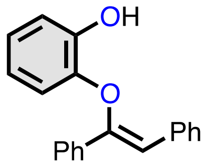

According to the general procedure for hydrophenoxylation, a crude product, which was prepared from diphenylacetylene (**1a**) (89.0 mg, 0.50 mmol), catechol (**2p**) (55.1 mg, 0.50 mmol) and [Au(IPr)]$_2$(μ-OH)][BF_4] (3.2 mg, 2.5 μmol, 0.5 mol %) in toluene (1 mL) at 80 °C for 6 h, was purified by column chromatography on silica gel (*n*-hexane/EtOAc = 95/5) to give **3ap** (100 mg, 70%, average of two runs) as a colourless liquid, whose NMR data were consistent to those reported in the literature [4].

^{1}H NMR (300 MHz; $CDCl_3$): δ 7.60-7.57 (m, 2H), 7.54-7.50 (m, 2H), 7.36-7.28 (m, 5H), 7.27-7.21 (m, 1H), 7.03 (dd, *J* = 8.0, 1.5 Hz, 1H), 6.88 (td, *J* = 7.7, 1.5 Hz, 1H), 6.75 (dd, *J* = 8.1, 1.5 Hz, 1H), 6.71 (s, 1H), 6.64 (ddd, *J* = 8.1, 7.4, 1.6 Hz, 1H), 5.87 (s, 1H). **^{13}C{^{1}H} NMR** (101 MHz, $CDCl_3$): δ 149.27, 145.79, 143.16, 135.25, 134.45, 128.91, 128.84, 128.77, 127.81, 125.79, 123.22, 120.62, 117.44, 115.78, 115.08.

1,2-Bis(((*Z*)-1,2-diphenylvinyl)oxy)benzene (4ap)

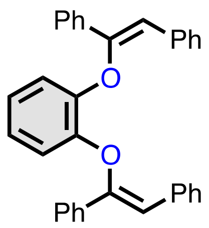

According to the general procedure for hydrophenoxylation, a crude product, which was prepared from diphenylacetylene (**1a**) (178.0 mg, 1 mmol), catechol (**2p**) (55.1 mg, 0.50 mmol) and [Au(IPr)]$_2$(μ-OH)][BF_4] (3.2 mg, 0.25 μmol, 0.5 mol %) in toluene (1 mL) at 80 °C for 18 h, was purified by column chromatography on silica gel (*n*-hexane/EtOAc = 95/5) to give **4ap** (256 mg, 55%, average of two runs) as a colourless liquid, whose NMR data were consistent to those reported in the literature [4].

^{1}H NMR (400 MHz; $CDCl_3$): δ 7.82-7.77 (m, 8H), 7.42-7.33 (m, 10H), 7.32-7.27 (m, 2H), 6.90-6.86 (dt, *J* = 6.0, 3.6 Hz, 2H), 6.76 (s, 2H), 6.69 (dt, *J* = 6.4, 3.3 Hz, 2H). **^{13}C{^{1}H} NMR** ($CDCl_3$, 101 MHz): δ 150.00, 145.53, 136.01, 135.01, 129.19, 128.78, 128.71, 128.66, 127.59, 126.16, 122.60, 117.04, 116.33, 77.48, 77.16, 76.84.

1,3-Bis[(*Z*)-1,2-diphenylvinyloxy]benzene (4aq)

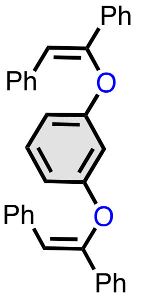

According to the general procedure for hydrophenoxylation, a crude product, which was prepared from diphenylacetylene (**1a**) (89.0 mg, 0.50 mmol), resorcinol (**2q**) (28.0 mg, 0.25 mmol) and [{Au(IPr)}$_2$(μ-OH)][BF_4] (3.2 mg, 2.5 μmol, 1.0 mol %) in toluene (1 mL) at 80 °C for 6 h, was purified by column chromatography on silica gel (*n*-hexane/EtOAc = 95/5) to give **4aq** (198 mg, 84%, average of two runs) as a white solid, whose NMR data were consistent to those reported in the literature [4].

^{1}H NMR ($CDCl_3$, 300 MHz) δ 7.61-7.53 (m, 4H), 7.52-7.43 (m, 4H), 7.34-7.16 (m, 12H), 7.02 (t, *J* = 8.2 Hz, 1H), 6.74 (t, *J* = 2.3 Hz, 1H), 6.60 (brs, 3H), 6.58 (d, *J* = 2.3 Hz, 1H); **^{13}C{^{1}H} NMR** ($CDCl_3$, 75 MHz) δ 157.7, 149.7, 135.9, 134.7, 130.4, 129.0, 128.6, 128.6, 128.4, 127.4, 126.1, 116.7, 110.2, 105.6.

1,4-Bis(((*Z*)-1,2-diphenylvinyl)oxy)benzene (4ar)

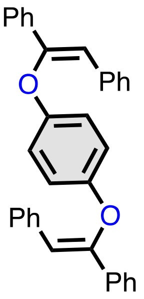

According to the general procedure for hydrophenoxylation, a crude product, which was prepared from alkyne **1a** (89.0 mg, 0.50 mmol), hydroquinone (**2r**) (28.0 mg, 0.25 mmol) and [{Au(IPr)}$_2$(μ-OH)][BF_4] (3.2 mg, 2.5 μmol, 0.5 mol %) in toluene (1 mL) at 80 °C for 1 h, was purified by trituration in pentane after evaporation of the volatiles, to give **4ar** (212 mg, 95%, average of two runs) as a colourless solid, whose NMR data were consistent to those reported in the literature [4].

^{1}H NMR (CD_2Cl_2, 500 MHz) δ 7.62-7.60 (m, 2H), 7.56-7.54 (m, 2H), 7.33-7.26 (m, 5H), 7.22-7.18 (m, 1H), 6.88 (s, 2H), 6.61 (s, 1H); **^{13}C{^{1}H} NMR** ($CDCl_3$, 75 MHz) δ 151.6, 150.7, 136.5, 135.3, 129.4, 129.1, 129.0, 127.9, 126.6, 117.6, 117.2.

1,4-Bis(((*Z*)-1,2-diphenylvinyl)oxy)-2,5-dichlorobenzene (4as)

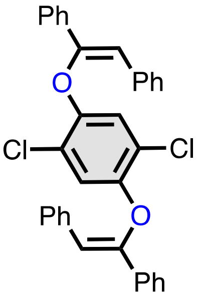

According to the general procedure for hydrophenoxylation, a crude product, which was prepared from alkyne (**1a**) (89.0 mg, 0.50 mmol), hydroquinone **2s** (45.0 mg, 0.25 mmol) and [{Au(IPr)}$_2$(μ-OH)][BF_4] (3.2 mg, 2.5 μmol, 0.5 mol %) in toluene (1 mL) at 80 °C for 16 h, was purified by trituration in pentane after evaporation of the volatiles, to give **4as** (64 mg, 47%, average of two runs) as an off-white solid.

^{1}H NMR (CD_2Cl_2, 400 MHz): δ 7.61-7.54 (m, 8H), 7.36-7.23 (m, 12H), 6.91 (s, 2H), 6.72 (s, 2H); **HRMS** (APCI) calcd for $C_{34}H_{26}O_2Cl_2$ [$(M+H)^+$] 534.1148, found 534.1140.

(*Z*)-4,4'-(1-Phenoxyethene-1,2-diyl)bis(methoxybenzene) (3bt)

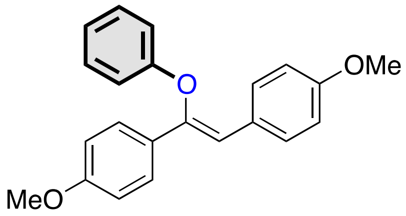

According to the general procedure for hydrophenoxylation, a crude product, which was prepared from alkyne (**1b**) (119.2 mg, 0.50 mmol), phenol (**2t**) (52.0 mg, 0.55 mmol) and [{Au(IPr)}$_2$(μ-OH)][BF$_4$] (6.5 mg, 5 μmol, 1.0 mol %) in toluene (1 mL) at 110 °C for 14 h, was purified by column chromatography on silica gel (pentane/EtOAc = 95/5) to give **2bt** (148 mg, 89%, average of two runs) as a off-white solid.
^{1}H NMR (300 MHz; CDCl$_3$): δ 7.57 (d, *J* = 8.8 Hz, 2H), 7.51 (d, *J* = 8.8 Hz, 2H), 7.26-7.18 (m, 2H), 7.03 (d, *J* = 7.8 Hz, 2H), 6.94 (t, *J* = 7.3 Hz, 1H), 6.82 (dd, *J* = 9.0, 2.6 Hz, 4H), 6.53 (s, 1H), 3.65 (s, 6H). **^{13}C{^{1}H} NMR** (101 MHz, CDCl$_3$): δ 159.60, 158.68, 156.54, 147.82, 130.16, 129.72, 128.72, 127.85, 127.19, 121.92, 116.26, 114.66, 114.06, 114.03, 55.32, 55.28. **HRMS** (APCI) calcd for $C_{22}H_{21}O_3$ [M$^+$] 333.1485, found 333.1482.

(*Z*)-4,4'-(1-Phenoxyethene-1,2-diyl)bis(chlorobenzene) (3ct)

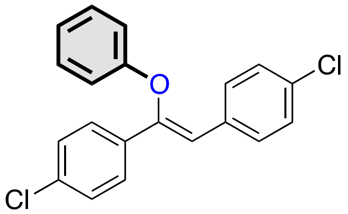

According to the general procedure for hydrophenoxylation, a crude product, which was prepared from alkyne **1c** (123.5 mg, 0.50 mmol), phenol (**2t**) (52.0 mg, 0.55 mmol) and [{Au(IPr)}$_2$(μ-OH)][BF$_4$] (6.5 mg, 5 μmol, 1.0 mol %) in toluene (1 mL) at 110 °C for 14 h, was purified by column chromatography on silica gel (pentane/EtOAc = 9/1) to give **3ct** (140 mg, 82%, average of two runs) as a off-white solid.
^{1}H NMR (300 MHz; CDCl$_3$): δ 7.58-7.54 (m, 2H), 7.52-7.48 (m, 2H), 7.30-7.21 (m, 6H), 6.99-6.95 (m, 3H), 6.58 (s, 1H). **^{13}C{^{1}H} NMR** (126 MHz, CDCl$_3$): 155.93, 149.23, 134.54, 134.32, 133.24, 133.06, 130.29, 129.93, 129.01, 128.87, 127.45, 122.57, 116.32, 115.99. **HRMS** (APCI) calcd for $C_{20}H_{15}Cl_2O$ [M$^+$] 341.0494, found 341.0491.

(*Z*)-[(1,4-Dimethoxybut-2-en-2-yl)oxy]benzene (3dt)

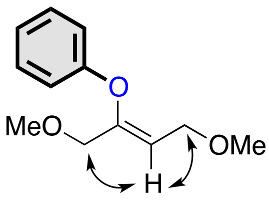

NOESY

According to the general procedure for hydrophenoxylation, a crude product, which was prepared from alkyne **1d** [5] (114.0 mg, 1.00 mmol), phenol (**2t**) (47.0 mg, 0.50 mmol) and [{Au(IPr)}$_2$(μ-OH)][BF$_4$] (3.2 mg, 2.5 μmol, 0.5 mol %) in toluene (1 mL) at 80 °C for 2 h, was purified by column chromatography on silica gel (*n*-hexane/EtOAc = 10/1) to give **3dt** (89 mg, 85%, average of two runs) as a colourless liquid.
^{1}H NMR (CDCl$_3$, 300 MHz) δ 7.34-7.23 (m, 2H), 7.08-6.93 (m, 3H), 5.51 (tt, *J* = 6.4, 0.9 Hz, 1H), 4.02 (dt, *J* = 6.4, 0.9 Hz, 2H), 3.91 (dt, *J* = 0.9 Hz, 2H), 3.35 (s, 3H), 3.31 (s, 3H); **^{13}C{^{1}H} NMR** (CDCl$_3$, 75 MHz) δ 156.3, 149.9, 129.7, 122.5, 116.5, 114.9, 70.6, 66.4, 58.4, 58.2; **HRMS** (APCI) calcd for $C_{12}H_{16}O_3$ [M$^+$] 208.1094, found 208.1093.

(*Z*)-2,2'-(1-Phenoxyethene-1,2-diyl)dithiophene (3et)

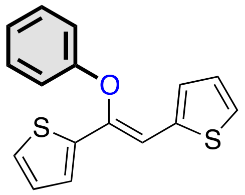

According to the general procedure for hydrophenoxylation, a crude product, which was prepared from alkyne **1e** [6] (95.0 mg, 0.50 mmol), phenol (**2t**) (52.0 mg, 0.55 mmol) and [{Au(IPr)}$_2$(μ-OH)][BF$_4$] (6.5 mg, 5 μmol, 1.0 mol %) in toluene (1 mL) at 110 °C for 1 h, was purified by column chromatography on silica gel (*n*-hexane/EtOAc = 9/1) to give **3et** (91 mg, 64%, average of two runs) as a off-white solid.
^{1}H NMR (CDCl$_3$, 400 MHz) δ 7.31-7.24 (m, 2H), 7.23-7.20 (m, 1H), 7.19 (ddd, *J* = 5.0, 1.0, 1.0 Hz, 1H), 7.17-7.13 (m, 1H), 7.10-7.07 (m, 3H), 7.04-6.95 (m, 3H), 6.94-6.90 (m, 1H); **^{13}C{^{1}H} NMR** (CDCl$_3$, 101 MHz) δ 156.0, 142.8, 139.1, 137.0, 129.8, 127.9, 127.7, 127.0, 126.6, 125.6, 125.4, 122.6, 116.0, 110.6; **HRMS** (APCI) calcd for $C_{16}H_{13}OS_2$ [(M+H)$^+$] 285.0402, found 285.0397.

(*Z*)-2,2'-(1-Phenoxyethene-1,2-diyl)bis(chlorobenzene) (3ft)

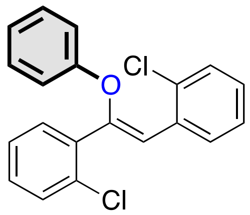

According to the general procedure for hydrophenoxylation, a crude product, which was prepared from alkyne **1f** (123.0 mg, 0.50 mmol), phenol (**2t**) (47.0 mg, 0.50 mmol) and [{Au(IPr)}$_2$(μ-OH)][BF_4] (3.2 mg, 2.5 μmol, 0.5 mol %) in toluene (1 mL) at 80 °C for 6 h, was purified by column chromatography on silica gel (pentane), to give **3ft** (148 mg, 87%, average of two runs) as a colourless oil.
^{1}H NMR ($CDCl_3$, 300 MHz) δ 8.18-8.15 (m, 1H), 7.66-7.62 (m, 1H), 7.50-7.47 (m, 1H), 7.44-7.41 (m, 1H), 7.33-7.10 (m, 6H), 7.17-7.13 (m, 2H), 7.05-7.00 (m, 1H), 6.93 (s, 1H); **^{13}C{^{1}H} NMR** ($CDCl_3$, 75 MHz) δ 155.7, 148.7, 136.1, 134.9, 133.6, 132.9, 132.6, 131.1, 130.6, 130.0, 129.8, 129.5, 128.5, 126.8, 126.5, 122.8, 117.6, 116.2; **HRMS** (ESI) calcd for $C_{20}H_{15}OCl_2$ [$(M+H)^+$] 341.0494, found 341.0495.

(*Z*)-(2-Phenoxybut-1-en-1-yl)benzene and (*Z*)-(1-phenoxybut-1-en-1-yl)benzene (3it /3it' = 1/0.23)

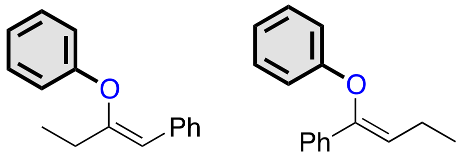

According to the general procedure for hydrophenoxylation, a crude product, which was prepared from **1i** (65.0 mg, 0.50 mmol), phenol (**2t**) (52.0 mg, 0.55 mmol) and [Au(IPr)]$_2$(μ-OH)][BF_4] (3.2 mg, 2.5 μmol, 0.5 mol %) in toluene (1 mL) at 80 °C for 6 h, was purified by column chromatography on silica gel (pentane/EtOAc = 95/5) to give the inseparable mixture of **3it** and **3it'** (**3it** /**3it'** = 1/0.23, 90 mg, 80%, average of two runs) as a colourless liquid.
^{1}H NMR (400 MHz; $CDCl_3$): δ 7.54-7.52 (m, 2H), 7.51-7.48 (m, 0.6H), 7.33-7.21 (m, 6H), 7.17-7.13 (m, 1H), 7.06 (t, *J* = 1.1 Hz, 0.32H), 7.04-7.01 (m, 3H), 6.98-6.95 (m, 0.5H), 6.93-6.91 (m, 0.15H), 5.94 (s, 1H), 5.87 (t, *J* = 7.3 Hz, 0.23H), 2.31 (qd, *J* = 7.4, 0.8 Hz, 2H), 2.23 (quintet, *J* = 7.5 Hz, 0.48H), 1.14 (t, *J* = 7.4 Hz, 3H), 1.04 (t, *J* = 7.5 Hz, 0.74H). **^{13}C{^{1}H} NMR** (126 MHz; $CDCl_3$): δ 157.61, 155.53, 154.39, 148.36, 135.56, 135.22, 129.73, 129.62, 128.54, 128.45, 128.38, 127.92, 126.68, 125.32, 122.44, 121.43, 119.87, 117.32, 115.48, 114.06, 26.41, 19.47, 13.97, 11.96. **HRMS** (APCI) calcd. for $C_{16}H_{17}O$ [$(M+H)^+$] 225.1274, found 225.1271.

(*Z*)-1-Methoxy-4-((1-phenylprop-1-en-2-yl)oxy)benzene and (*Z*)-1-methoxy-4-((1-phenylprop -1-en-1-yl)oxy)benzene (3ju /3ju = 1/0.22)

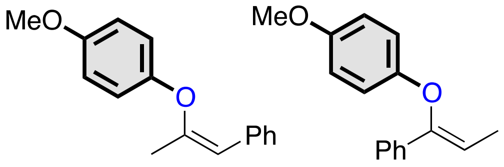

According to the general procedure for hydrophenoxylation, a crude product, which was prepared from **1j** (58.0 mg, 0.50 mmol), phenol **2u** (68.3 mg, 0.55 mmol) and [Au(IPr)]$_2$(μ-OH)][BF_4] (3.2 mg, 2.5 μmol, 0.5 mol %) in toluene (1 mL) at 80 °C for 6 h, was purified by column chromatography on silica gel (*n*-hexane/EtOAc = 95/5) to give the inseparable mixture of **3ju** and **3ju'** (**3ju** /**3ju'** = 1/0.22, 104 mg, 87%, average of two runs) as a colourless liquid.
^{1}H NMR (400 MHz; $CDCl_3$): δ 7.58-7.55 (m, 2H), 7.49-7.46 (m, 0.45), 7.30-7.23 (m, 3H), 7.17-7.13 (m, 1H), 6.99-6.95 (m, 2H), 6.91-6.83 (m, 3H), 6.79-6.76 (m, 0.40), 5.89 (q, *J* = 7.0 Hz, 0.23), 5.78 (s, 1H), 3.79 (s, 3H), 3.73 (s, 0.62H), 1.93 (s, 3H), 1.77 (d, *J* = 7.0 Hz, 0.62H). **^{13}C{^{1}H} NMR** (126 MHz; $CDCl_3$): δ 155.50, 154.23, 151.33, 150.17, 150.09, 149.03, 135.75, 135.62, 128.50, 128.41, 128.16, 127.84, 126.38, 125.32, 121.65, 119.23, 116.17, 114.77, 114.75, 113.55, 112.35, 110.03, 108.34, 55.75, 55.71, 19.69, 11.52. **HRMS** (APCI) calcd. for $C_{16}H_{17}O_2$ [$(M+H)^+$] 241.1223, found 241.1223.

(*Z*)-3-(2-Phenoxyhex-1-en-1-yl)pyridine (3nt) and (*Z*)-3-(1-phenoxyhex-1-en-1-yl)pyridine (3nt') (3nt/3nt' =1/0.43)

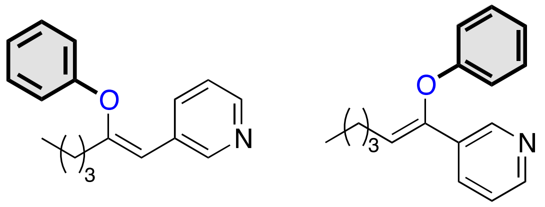

According to the general procedure for hydrophenoxylation, a crude product, which was prepared from alkyne **1n** (79.0 mg, 0.50 mmol), phenol (**2t**) (47.0 mg, 0.50 mmol) and [{Au(IPr)}$_2$(μ-OH)][BF$_4$] (19.5 mg, 15 μmol, 3.0 mol %) in toluene (1 mL) at 110 °C for 24 h, was purified by column chromatography on silica gel (*n*-hexane/EtOAc = 95/5~70/30) to give **3nt** and **3nt'** (**3nt** / **3nt'** = 1/0.43, 96 mg, 76%, average of two runs) as a pale yellow oil.

^{1}H NMR (CDCl$_3$, 300 MHz) δ 8.77 (d, *J* = 1.8 Hz, 1H), 8.63 (br, 2.25H), 8.45 (dd, *J* = 4.7, 1.4 Hz, 1H), 8.36 (d, *J* = 3.7 Hz, 2.33H), 7.94 (dt, *J* = 8.1, 1.9 Hz, 2.36H), 7.71 (dt, *J* = 8.0, 2.0 Hz, 1H), 7.34-7.12 (m, 12H), 7.08-6.92 (m, 10H), 5.95-5.89 (m, 1+2.21H), 2.33-2.20 (m, 6.62H), 1.58-1.21 (m, 15.38H), 0.89 (t, *J* = 7.3 Hz, 10.38H); **^{13}C{^{1}H} NMR** (CDCl$_3$, 75 MHz) δ 157.0, 155.7, 155.1, 149.7, 148.8, 147.5, 147.1, 146.4, 134.9, 132.6, 131.3, 129.8, 129.8, 123.5, 123.3, 122.9, 121.9, 120.4, 117.4, 115.6, 111.2, 32.9, 31.4, 29.3, 25.7, 22.6, 22.2, 14.0, 14.0. **HRMS** (ESI) calcd for $C_{17}H_{20}O_1N_1$ [(M+H)$^+$] 254.1539, found 254.1541.

(*Z*)-[{4-(Benzyloxy)but-2-en-2-yl}oxy]benzene (3ot)

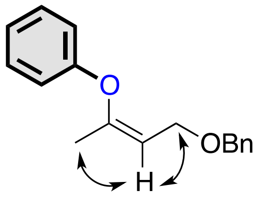

NOESY

According to the general procedure for hydrophenoxylation, a crude product, which was prepared from alkyne **1o** [7] (160.0 mg, 1.00 mmol), phenol (**2t**) (47.0 mg, 0.50 mmol) and [{Au(IPr)}$_2$(μ-OH)][BF$_4$] (3.2 mg, 2.5 μmol, 0.5 mol %) in toluene (1 mL) at 80 °C for 2 h, was purified by column chromatography on silica gel (*n*-hexane/EtOAc = 95/5) to give **3ot** (89 mg, 70%, average of two runs) as a colourless liquid.

^{1}H NMR (CDCl$_3$, 300 MHz) δ 7.34-7.18 (m, 7H), 7.03-6.94 (m, 1H), 6.94-6.86 (m, 2 H), 5.21 (t, *J* = 6.8 Hz, 1H), 4.43 (s, 2H), 4.06 (d, *J* = 6.8 Hz, 2H), 1.81 (s, 3H); **^{13}C{^{1}H} NMR** (CDCl$_3$, 101 MHz) δ 156.1, 150.9, 138.6, 129.7, 128.4, 127.9, 127.6, 122.4, 117.0, 112.6, 72.4, 64.5, 18.6; **HRMS** (APCI) calcd for $C_{17}H_{19}O_2$ [(M+H)$^+$] 255.1380, found 255.1375.

(*Z*)-[{5-(Benzyloxy)pent-2-en-2-yl}oxy]benzene (3pt) and (*Z*)-[{5-(benzyloxy)pent-2-en-3-yl} oxy]benzene (3pt') (3pt/3pt' =1/0.17)

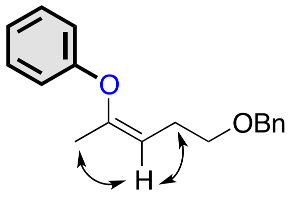

NOESY

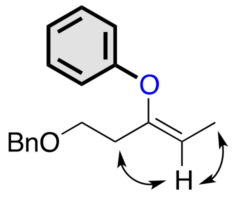

NOESY

According to the general procedure for hydrophenoxylation, a crude product, which was prepared from alkyne **1p** [8] (87.0 mg, 0.50 mmol), phenol (**2t**) (52.0 mg, 0.55 mmol) and [{Au(IPr)}$_2$(μ-OH)][BF$_4$] (3.2 mg, 2.5 μmol, 0.5 mol %) in toluene (1 mL) at 80 °C for 3 h, was purified by column chromatography on silica gel (*n*-hexane/EtOAc = 95/5) to give **3pt** and **3pt'** (**3pt** /**3pt'** = 1/0.17, 114 mg, 85%, average of two runs) as a colourless liquid.

^{1}H NMR (CDCl$_3$, 300 MHz) δ 7.33-7.14 (m, 7+1.19H), 6.96-6.80 (m, 3+0.51H), 5.12 (q, *J* = 6.7 Hz, 0.17 H), 5.03 (tq, *J* = 7.2, 1.1 Hz, 1H), 4.43 (s, 2H), 4.42 (s, 0.34H), 3.51 (t, *J* = 6.7 Hz, 0.34H), 3.41 (t, *J* = 7.2 Hz, 2H), 2.45-2.36 (m, 0.34H), 2.31 (dtq, *J* = 7.2, 7.2 1.1 Hz, 2H), 1.75 (dt, *J* = 7.2, 1.1 Hz, 3H), 1.50-1.47 (m, 0.51H); **^{13}C{^{1}H} NMR** (CDCl$_3$, 75 MHz) δ 156.6, 156.4, 148.6, 148.4, 138.7, 138.5, 129.6, 129.6, 128.5, 128.4, 127.8, 127.8, 127.7, 127.6, 121.8, 121.6, 116.4, 115.9, 112.7, 112.3, 73.0, 72.8, 69.8, 67.5, 33.5, 26.0, 18.5, 10.9.

3. References

[1] a) R. S. Ramón, S. Gaillard, A. Poater, L. Cavallo, A. M. Z. Slawin, S. P. Nolan, *Chem. Eur. J.* **2011**, *17*, 1238-1246; b) A. Gómez-Suárez, Y. Oonishi, S. Meiries, S. P. Nolan, *Organometallics* **2013**, *32*, 1106-1111.

[2] Y. Oonishi, A. Gomez-Suarez, A. R. Martin, Y. Makida, A. M. Slawin, S. P. Nolan, *Chem. Eur. J.* **2014**, *20*, 13507-13510.

[3] M. R. Kuram, M. Bhanuchandra, A. K. Sahoo, *J. Org. Chem.* **2010**, *75*, 2247-2258.

[4] H. Hiranuma, S. I. Miller, *J. Org. Chem.* **1982**, *47*, 5083-5088.

[5] P. Chuentragool, K. Vongnam, P. Rashatasakhon, M. Sukwattanasinitt, S. Wacharasindhu, *Tetrahedron* **2011**, *67*, 8177-8182.

[6] K. Semba, T. Fujihara, J. Terao, Y. Tsuji, *Chem. Eur. J.* **2012**, *18*, 4179-4184.

[7] F. Alonso, I. Osante, M. Yus, *Tetrahedron* **2007**, *63*, 93-102.

4. NMR Spectra

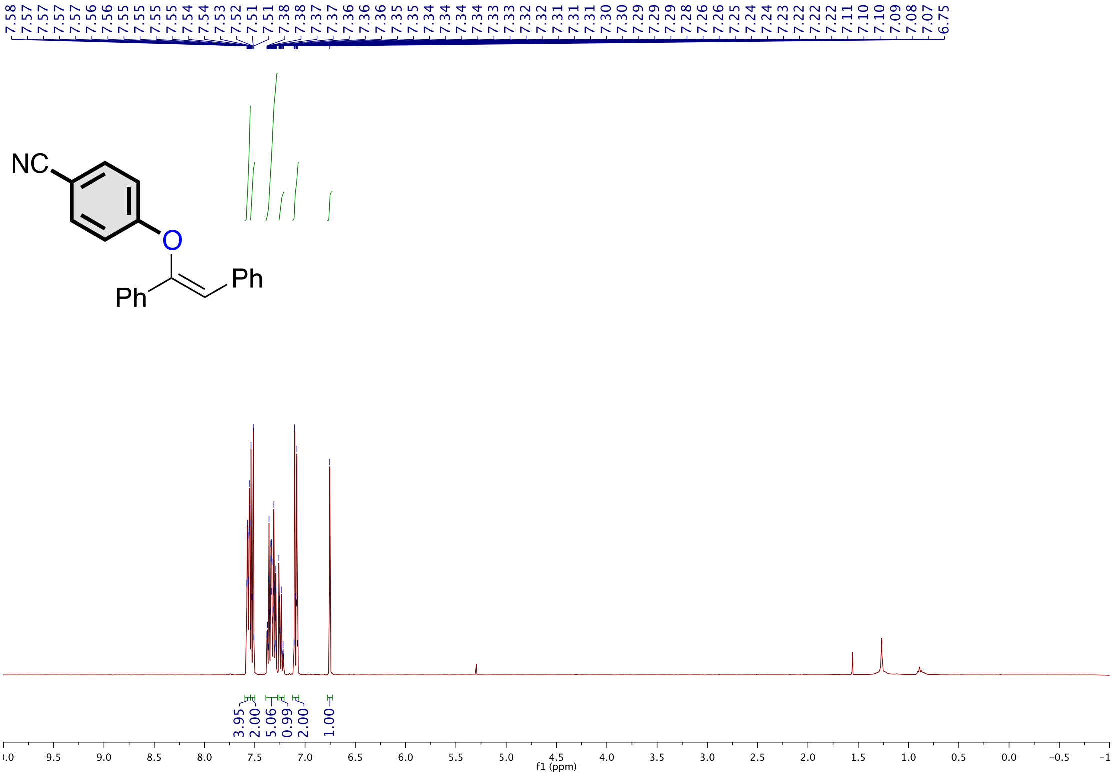

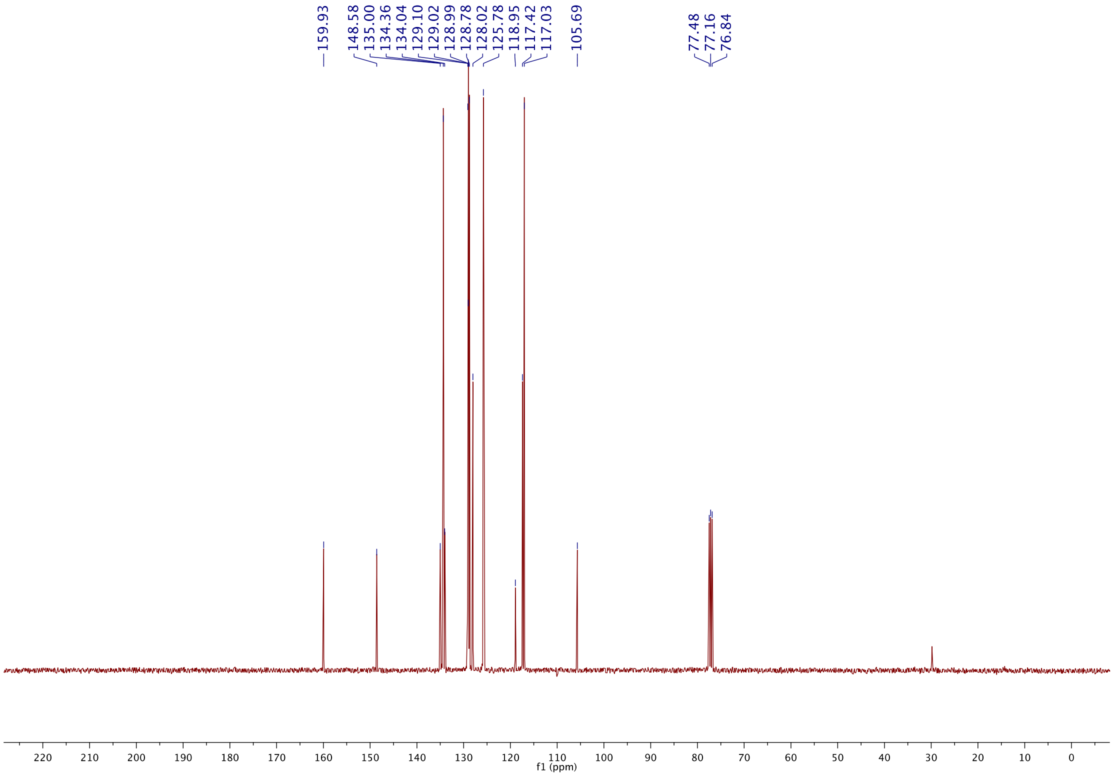

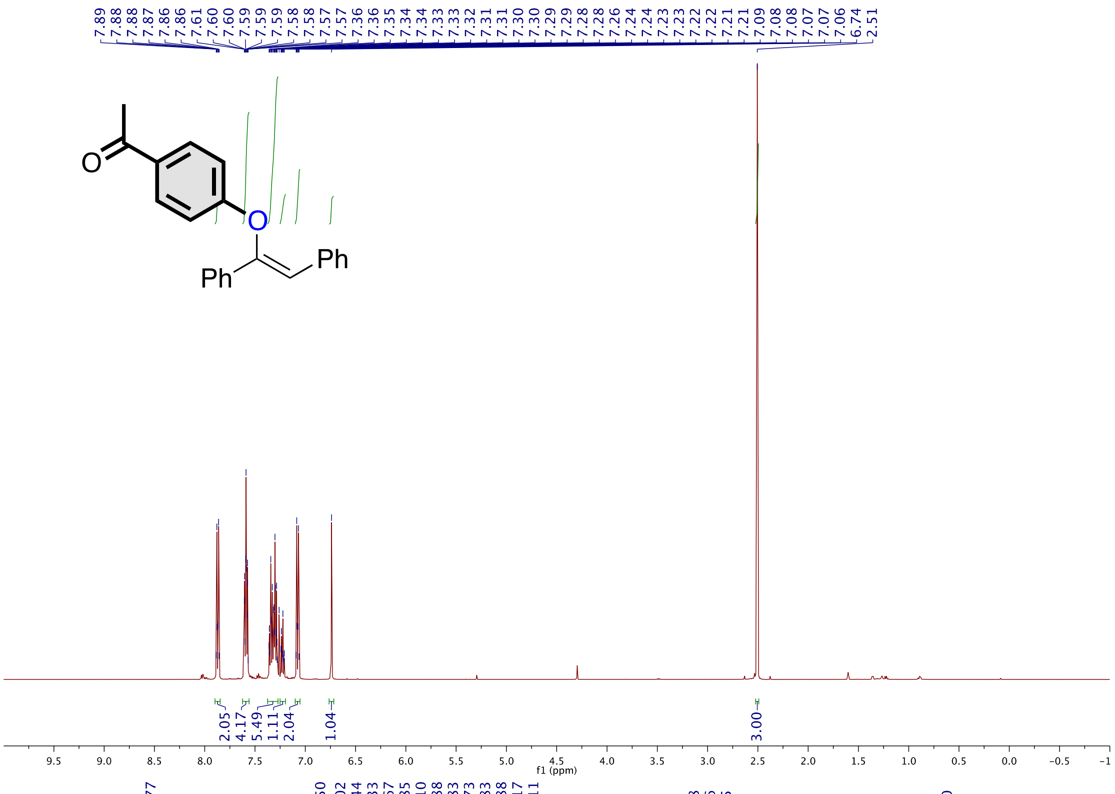
7.89
7.88
7.88
7.87
7.86
7.86
7.61
7.60
7.60
7.59
7.59
7.59
7.58
7.58
7.57
7.57
7.36
7.36
7.35
7.34
7.34
7.33
7.33
7.32
7.31
7.31
7.30
7.30
7.29
7.29
7.28
7.28
7.26
7.24
7.24
7.23
7.23
7.22
7.22
7.21
7.21
7.09
7.08
7.08
7.07
7.07
7.06
6.74
2.51
O
O
Ph
Ph
2.05
4.17
5.49
1.11
2.04
1.04
3.00
f1 (ppm)

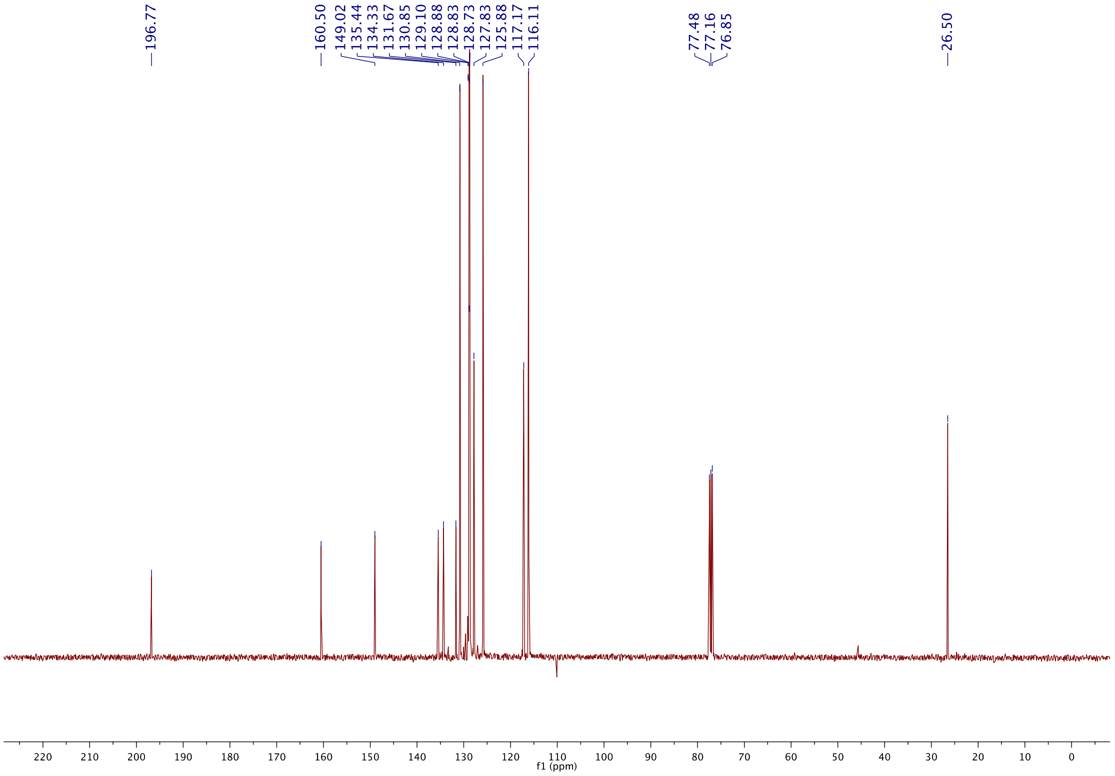
196.77
160.50
149.02
135.44
134.33
131.67
130.85
129.10
128.88
128.83
128.73
127.83
125.88
117.17
116.11
77.48
77.16
76.85
26.50
f1 (ppm)

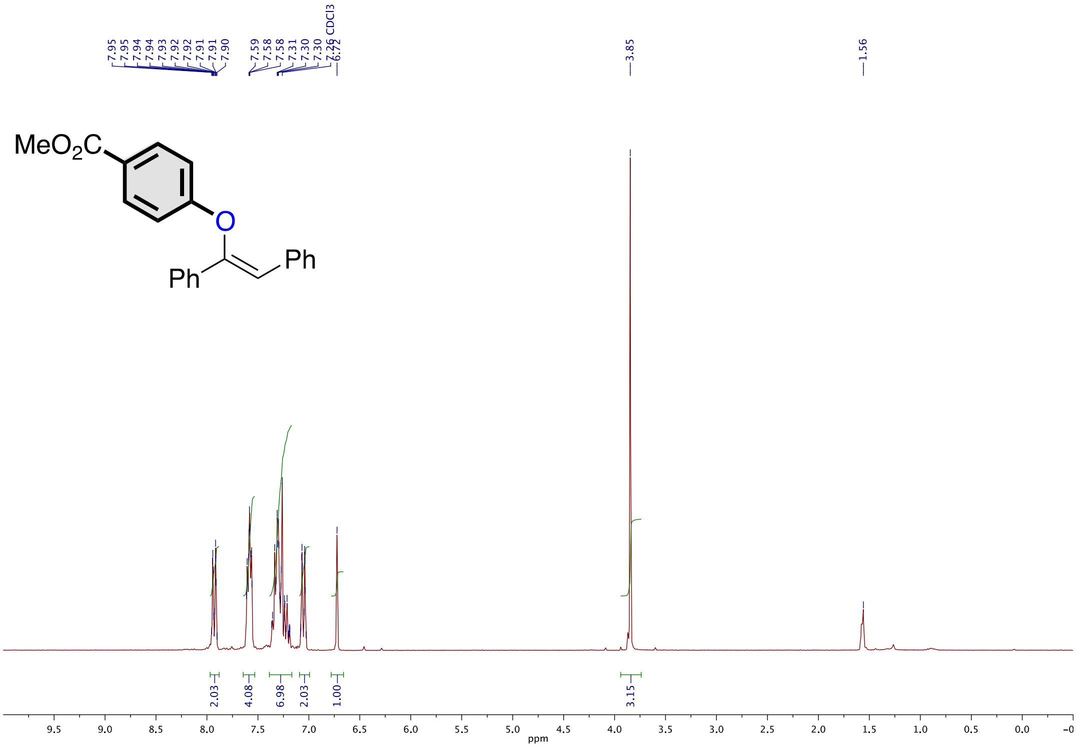
7.95
7.95
7.94
7.94
7.93
7.92
7.92
7.91
7.91
7.90
7.59
7.58
7.58
7.31
7.30
7.30
7.26 CDCl3
6.72
3.85
1.56
MeO2C
O
Ph
Ph
2.03
4.08
6.98
2.03
1.00
3.15
ppm

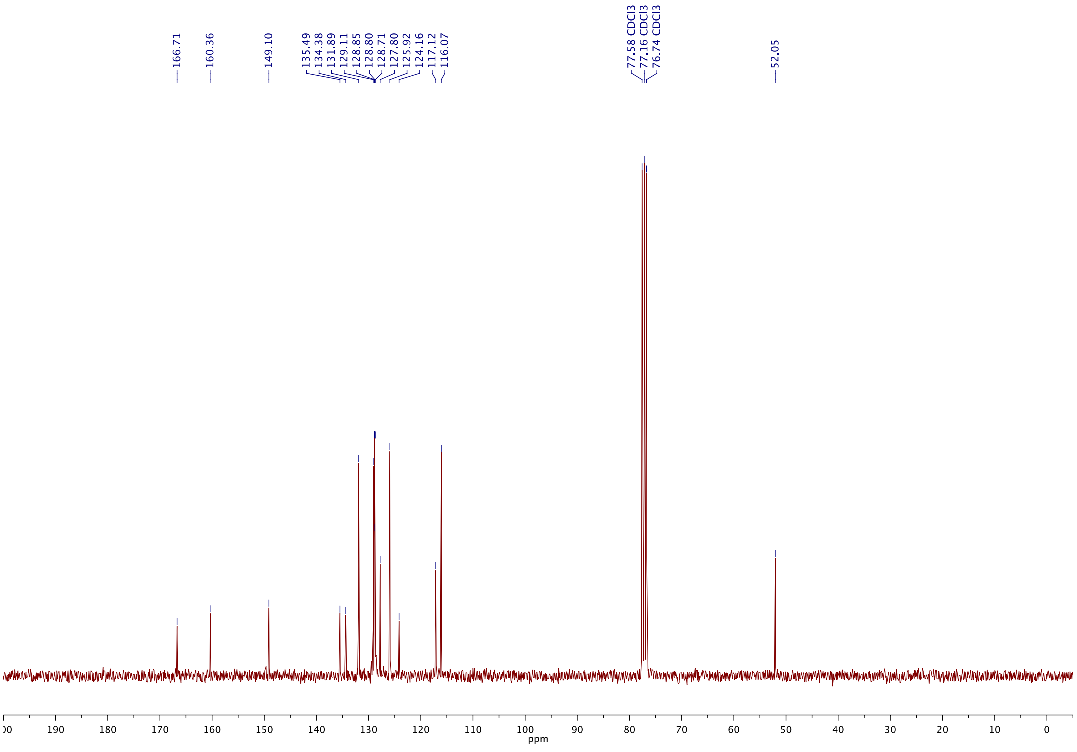
166.71
160.36
149.10
135.49
134.38
131.89
129.11
128.85
128.80
128.71
127.80
125.92
124.16
117.12
116.07
77.58 CDCl3
77.16 CDCl3
76.74 CDCl3
52.05
ppm

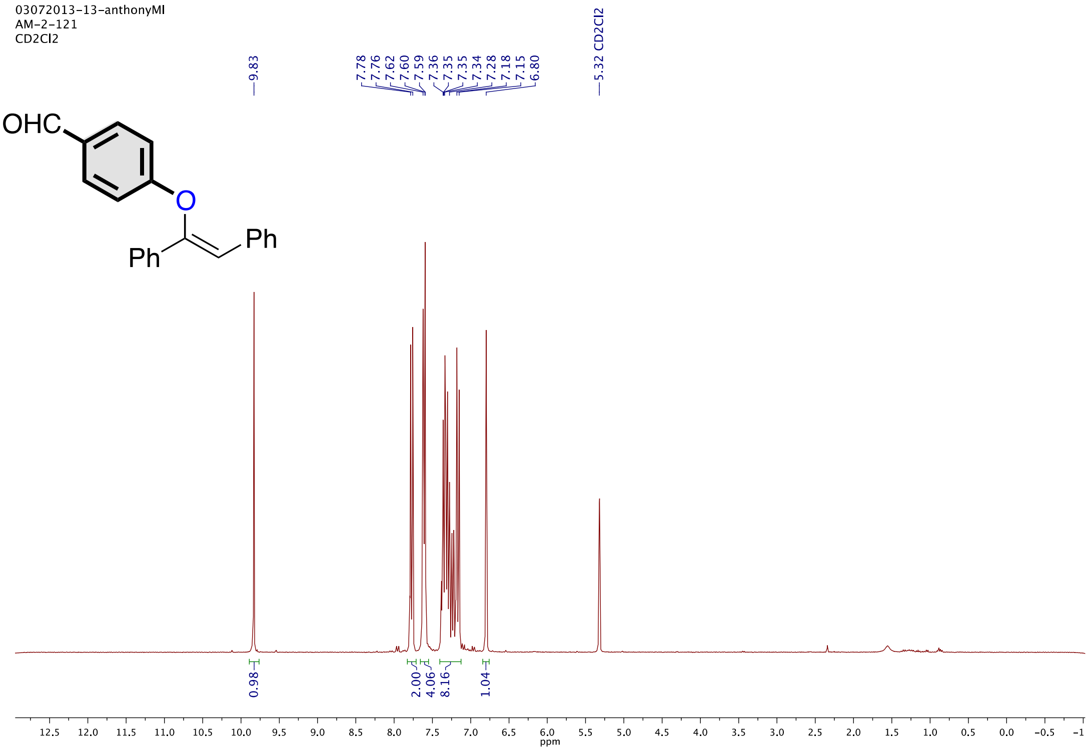
03072013-13-anthonyMI
AM-2-121
CD2Cl2
9.83
7.78
7.76
7.62
7.60
7.59
7.36
7.35
7.35
7.34
7.28
7.18
7.15
6.80
5.32 CD2Cl2
OHC
O
Ph
Ph
0.98
2.00
4.06
8.16
1.04
ppm

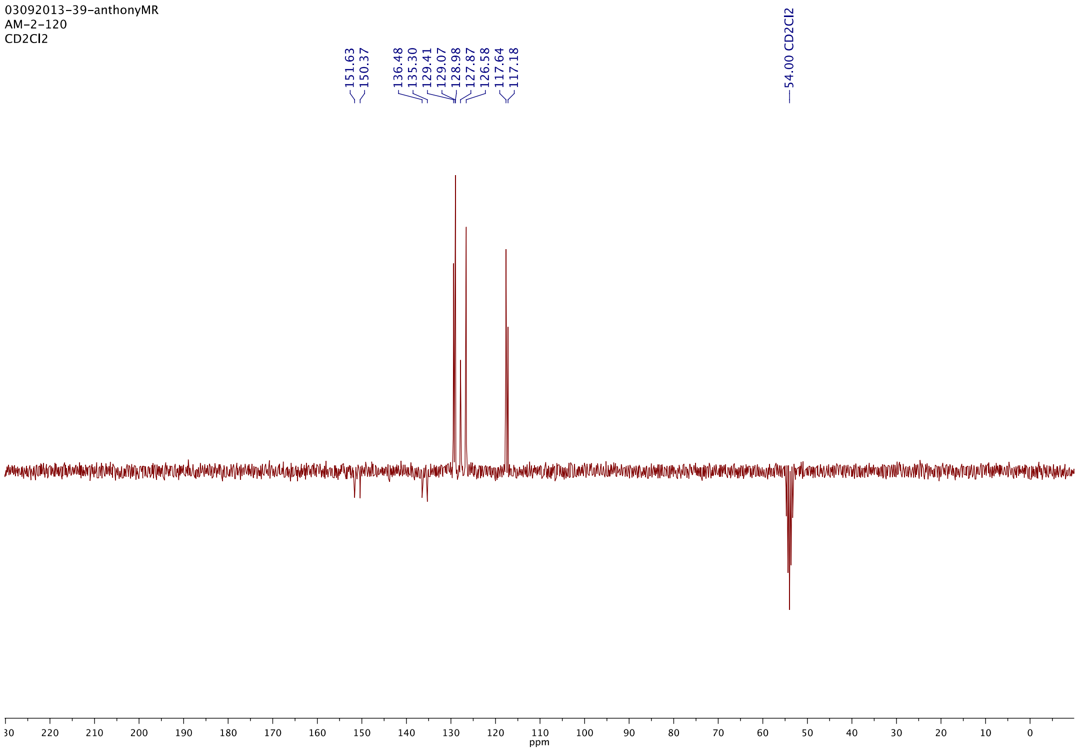
03092013-39-anthonyMR
AM-2-120
CD2Cl2
151.63
150.37
136.48
135.30
129.41
129.07
128.98
127.87
126.58
117.64
117.18
54.00 CD2Cl2
ppm

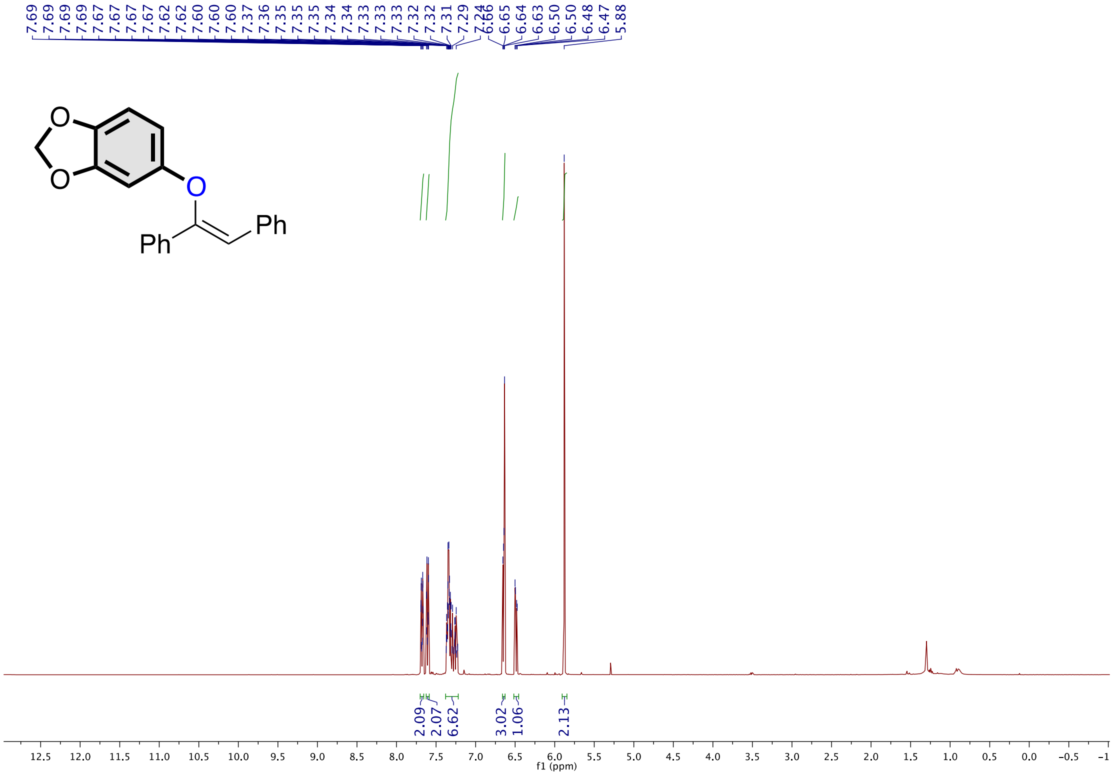
7.69
7.69
7.69
7.67
7.67
7.67
7.62
7.62
7.60
7.60
7.37
7.36
7.35
7.35
7.34
7.33
7.33
7.32
7.32
7.31
7.29
7.24
6.66
6.65
6.64
6.63
6.50
6.50
6.48
6.47
5.88
O
O
O
Ph
Ph
2.09
2.07
6.62
3.02
1.06
2.13
f1 (ppm)

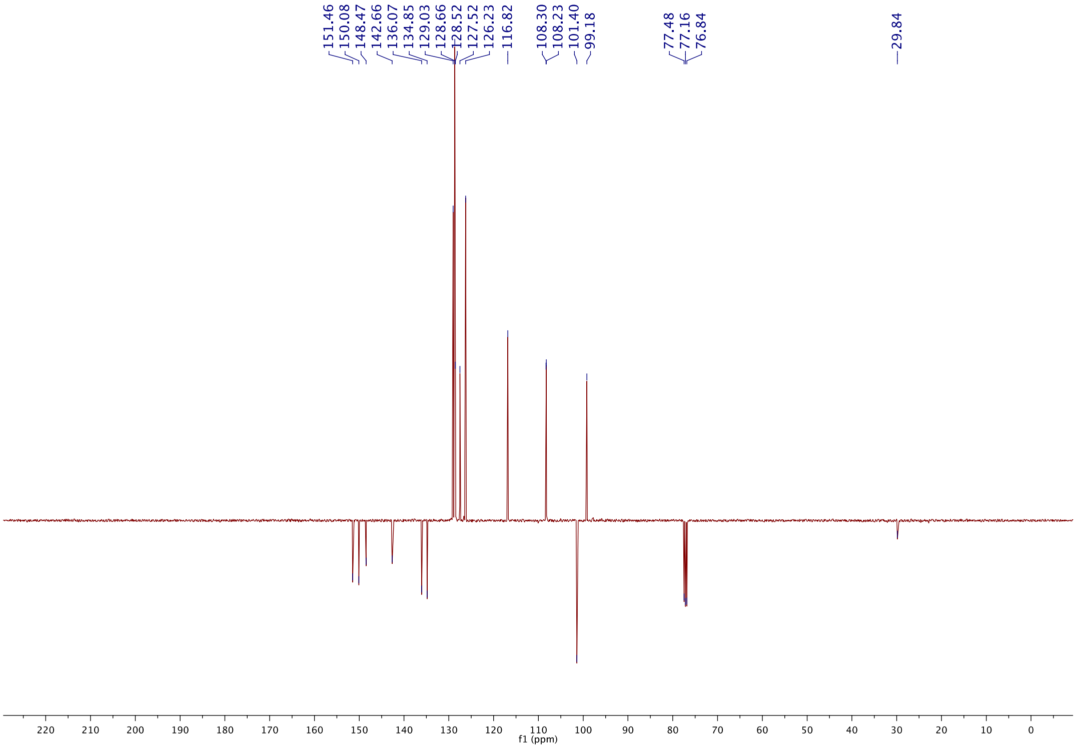
151.46
150.08
148.47
142.66
136.07
134.85
129.03
128.66
128.52
127.52
126.23
116.82
108.30
108.23
101.40
99.18
77.48
77.16
76.84
29.84
f1 (ppm)

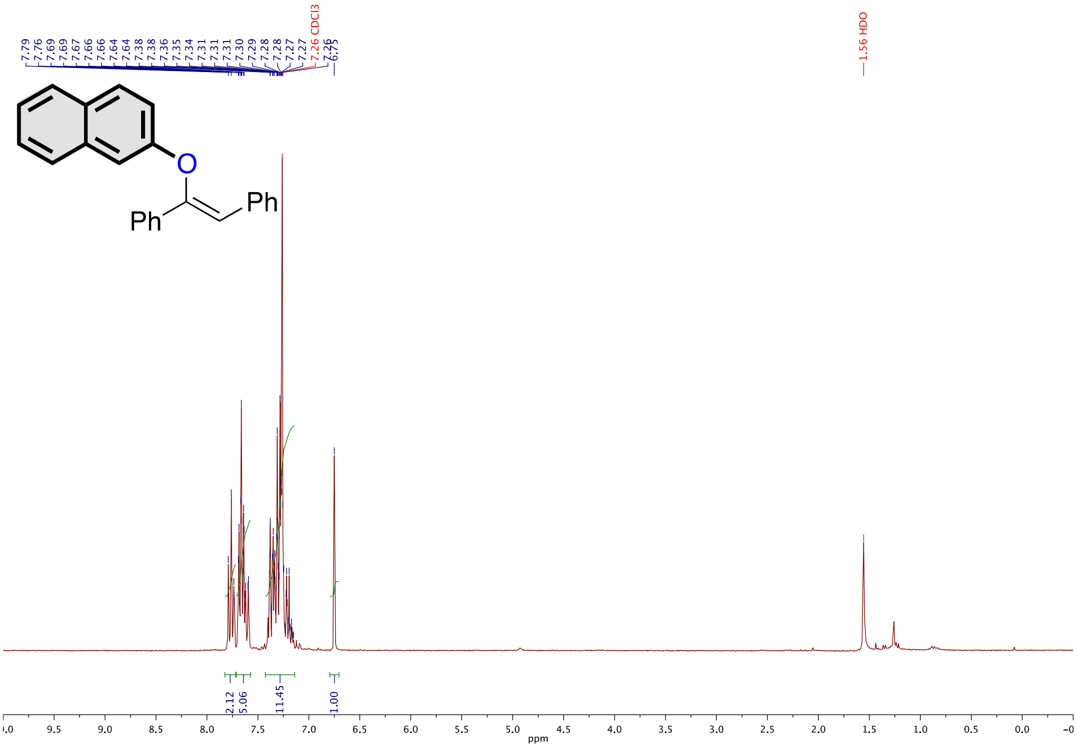
7.79
7.76
7.69
7.69
7.67
7.66
7.66
7.64
7.64
7.38
7.38
7.36
7.35
7.34
7.31
7.31
7.30
7.29
7.28
7.28
7.27
7.27
7.26 CDCl3
7.26
6.75
1.56 HDO
O
Ph
Ph
2.12
5.06
11.45
1.00
ppm

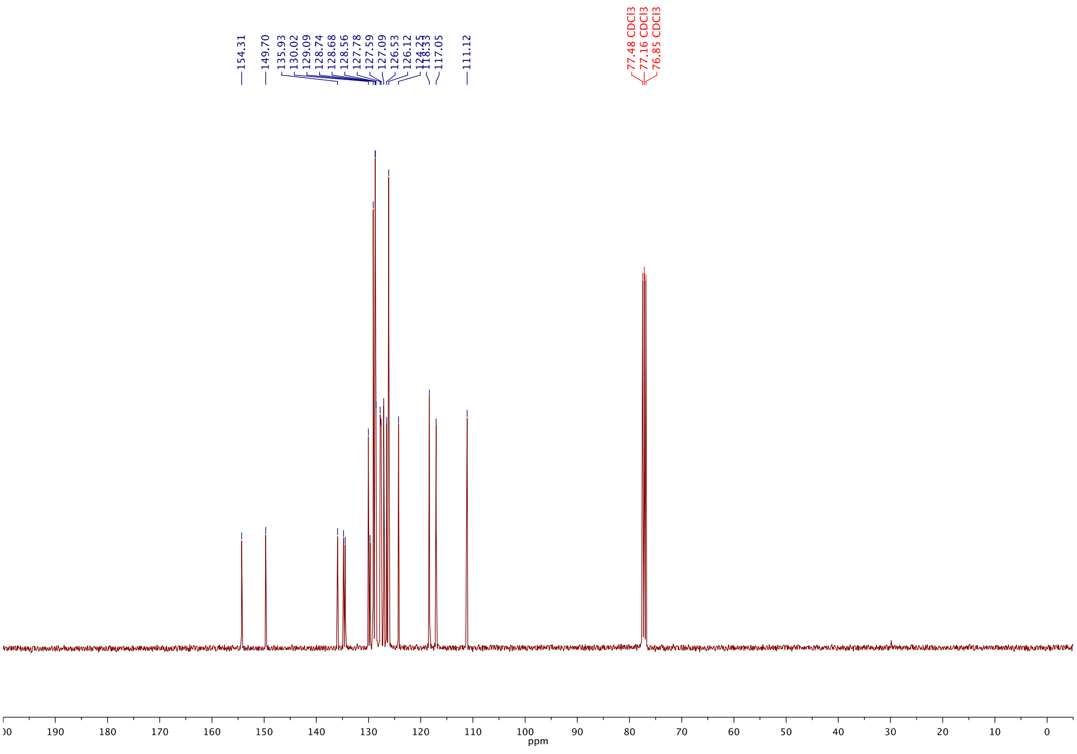
154.31
149.70
135.93
130.02
129.09
128.74
128.68
128.56
127.78
127.59
127.09
126.53
126.12
124.25
118.33
117.05
111.12
77.48 CDCl3
77.16 CDCl3
76.85 CDCl3
ppm

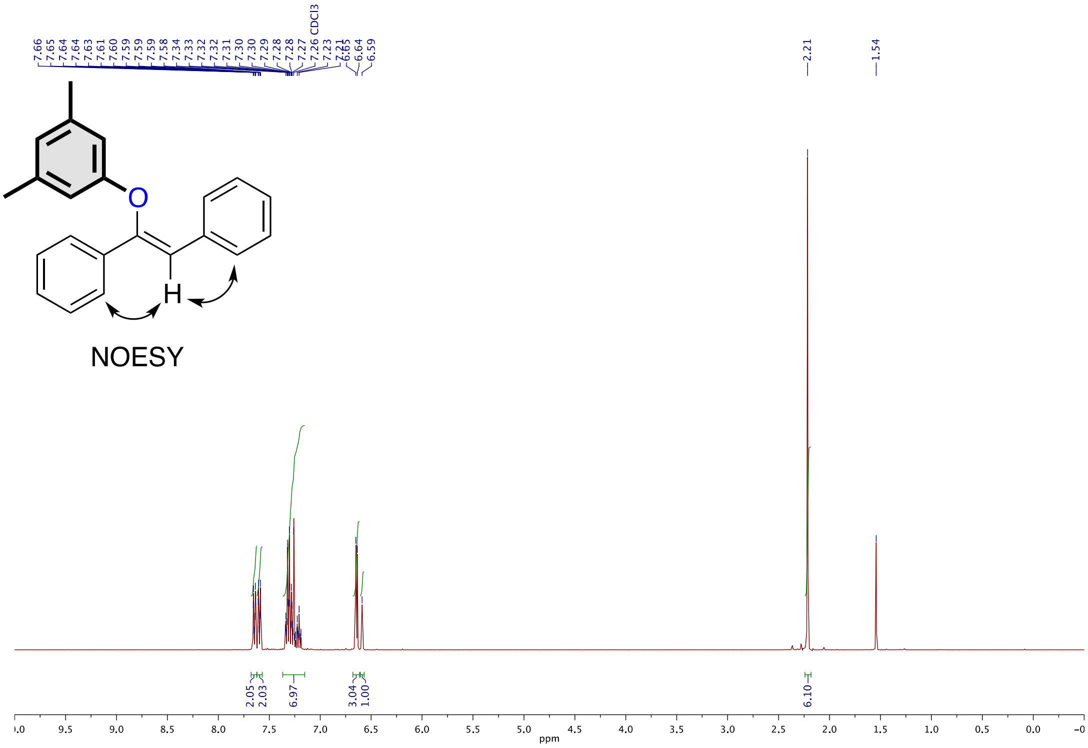
7.66
7.65
7.64
7.63
7.61
7.60
7.59
7.59
7.58
7.34
7.33
7.32
7.32
7.31
7.30
7.30
7.29
7.28
7.28
7.27
7.26 CDCl3
7.23
7.21
6.65
6.64
6.59
2.21
1.54
O
H
NOESY
2.05
2.03
6.97
3.04
1.00
6.10
ppm

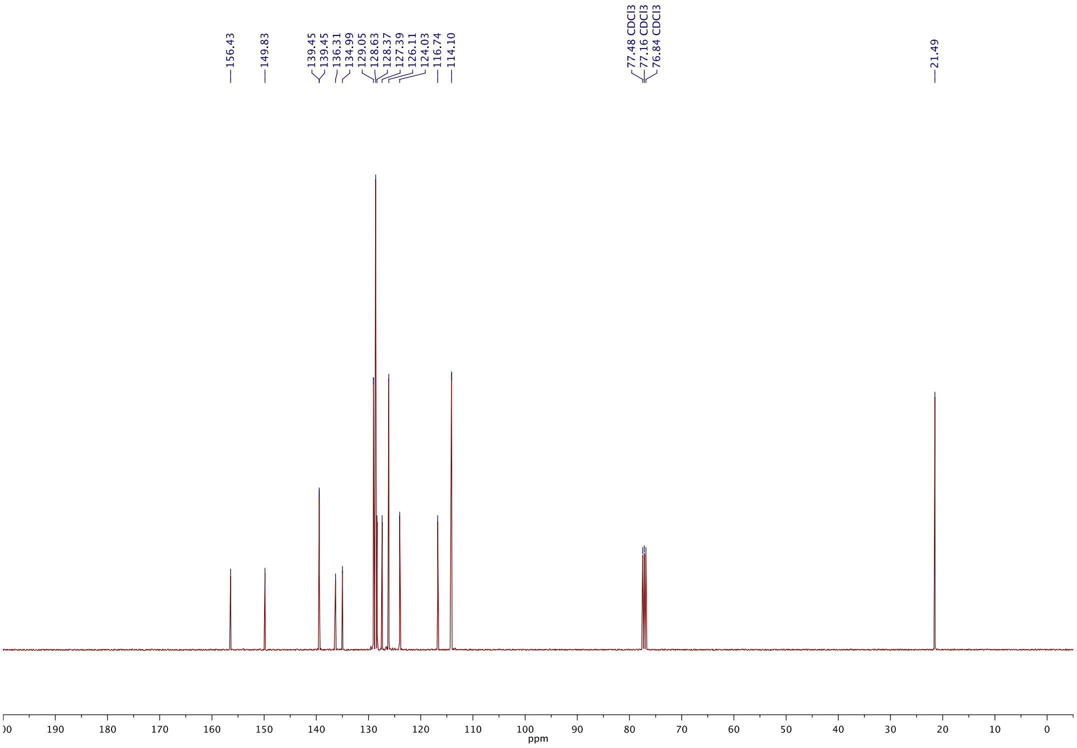
156.43
149.83
139.45
139.45
136.31
134.99
129.05
128.63
128.37
127.39
126.11
124.03
116.74
114.10
77.48 CDCl3
77.16 CDCl3
76.84 CDCl3
21.49
ppm

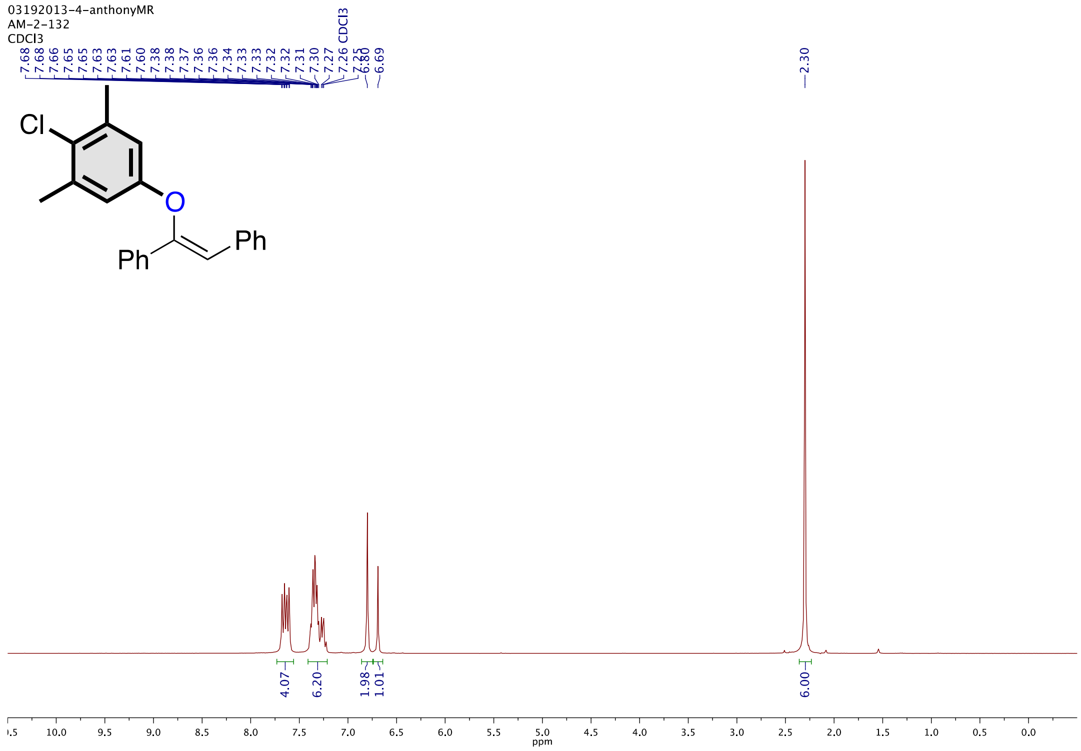

03192013-4-anthonyMR
AM-2-132
CDCl3
7.68
7.68
7.66
7.65
7.65
7.63
7.63
7.61
7.60
7.38
7.38
7.37
7.36
7.36
7.34
7.33
7.33
7.32
7.32
7.31
7.30
7.27
7.26 CDCl3
7.25
6.80
6.69
2.30
Cl
O
Ph
Ph
4.07
6.20
1.98
1.01
6.00
ppm

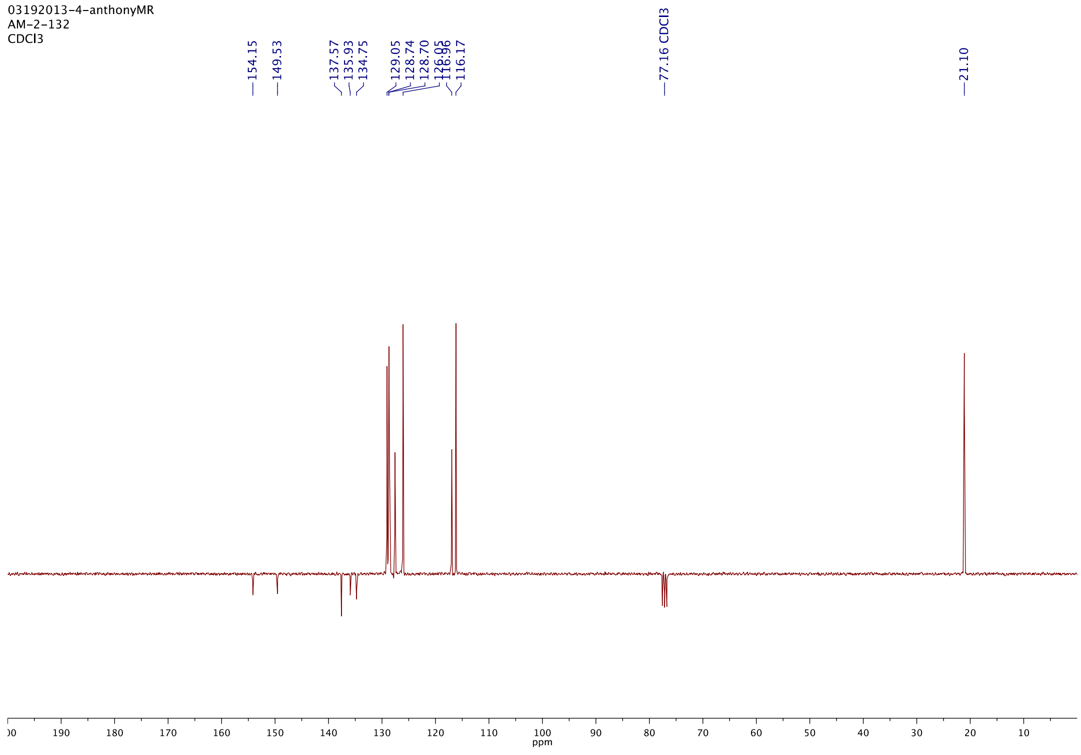

03192013-4-anthonyMR
AM-2-132
CDCl3
154.15
149.53
137.57
135.93
134.75
129.05
128.74
128.70
126.05
116.96
116.17
77.16 CDCl3
21.10
ppm

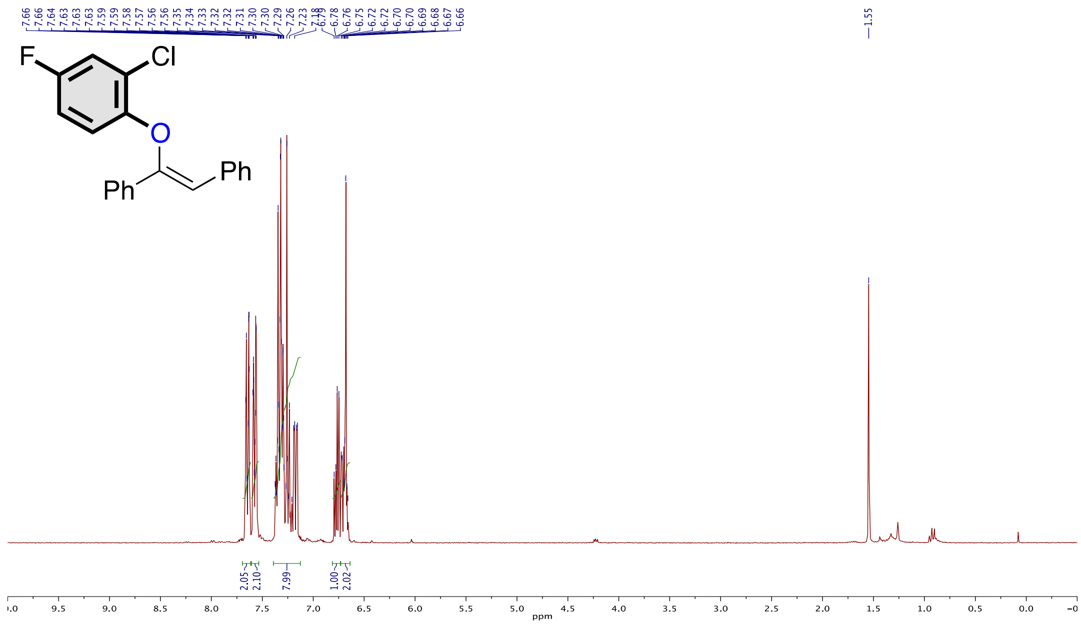
7.66
7.66
7.64
7.63
7.63
7.63
7.59
7.59
7.58
7.57
7.56
7.56
7.35
7.34
7.33
7.32
7.32
7.31
7.30
7.30
7.29
7.26
7.23
7.18
6.79
6.78
6.76
6.75
6.72
6.72
6.70
6.70
6.69
6.68
6.67
6.66
1.55
F
Cl
O
Ph
Ph
2.05
2.10
7.99
1.00
2.02
ppm

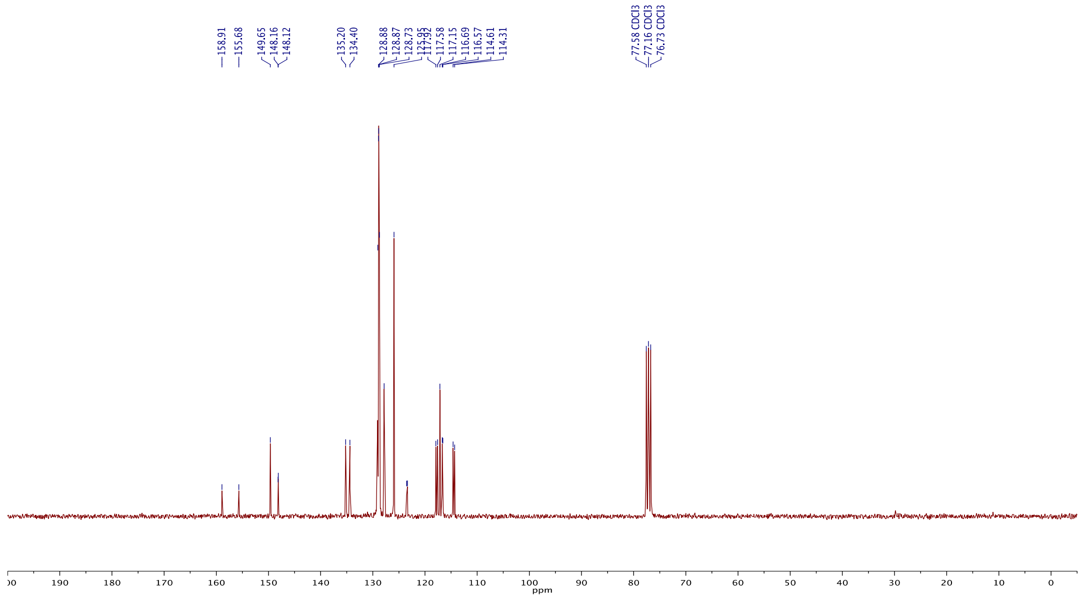
158.91
155.68
149.65
148.16
148.12
135.20
134.40
128.88
128.87
128.73
117.58
117.15
116.69
116.57
114.61
114.31
77.58 CDCl3
77.16 CDCl3
76.73 CDCl3
ppm

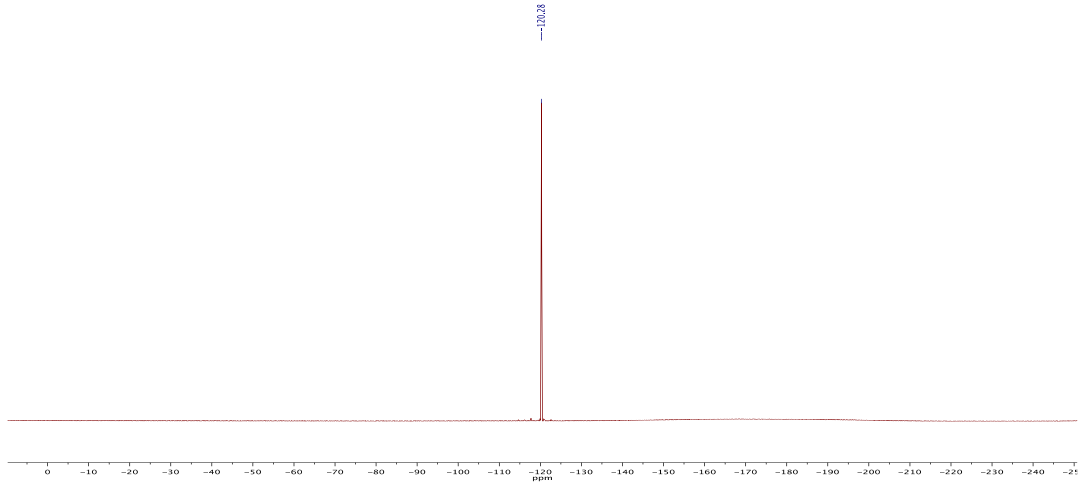
-120.28
ppm

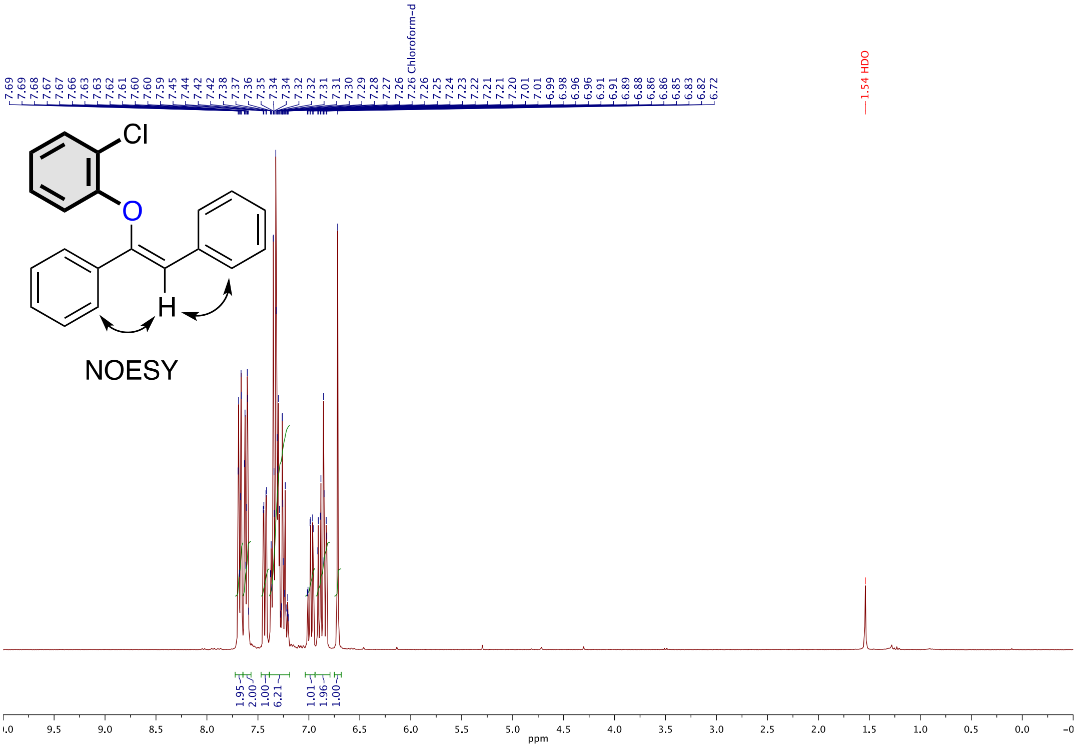
7.69
7.69
7.68
7.67
7.67
7.66
7.63
7.63
7.62
7.61
7.60
7.59
7.45
7.44
7.42
7.42
7.38
7.37
7.36
7.35
7.34
7.34
7.32
7.32
7.31
7.31
7.30
7.29
7.28
7.27
7.26 Chloroform-d
7.26
7.25
7.24
7.23
7.22
7.21
7.21
7.20
7.01
7.01
6.99
6.98
6.96
6.96
6.91
6.91
6.89
6.88
6.86
6.86
6.85
6.83
6.82
6.72
1.54 HDO
Cl
O
H
NOESY
1.95
2.00
1.00
6.21
1.01
1.96
1.00
ppm

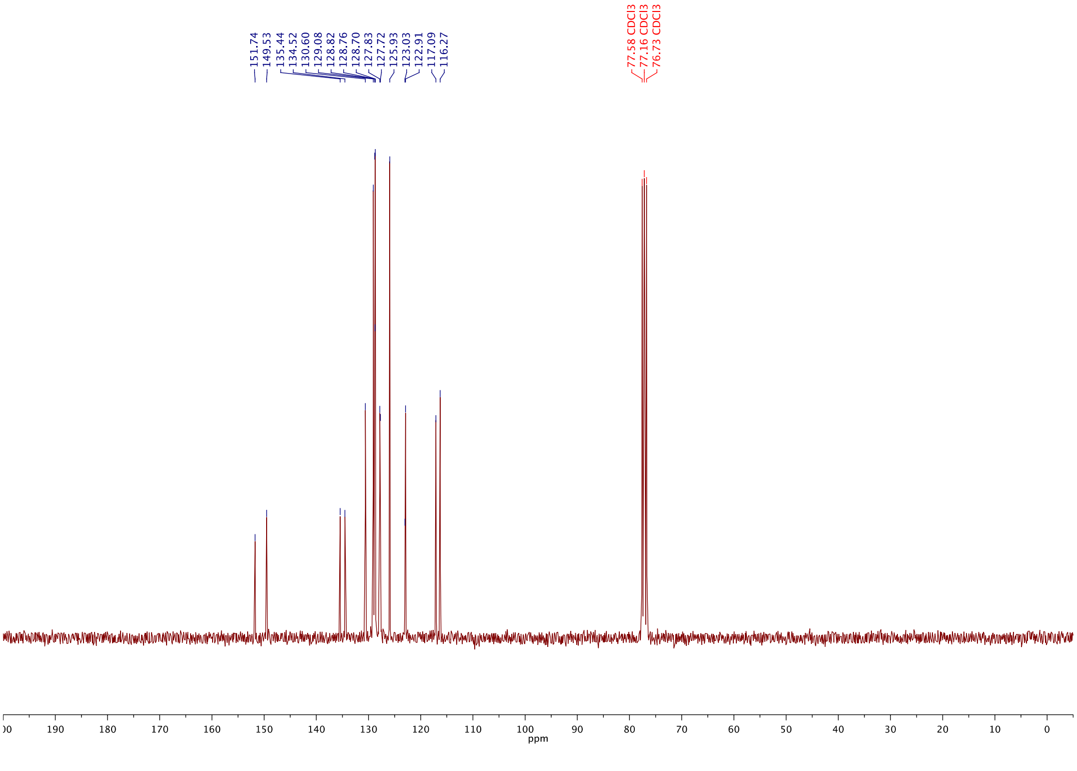
151.74
149.53
135.44
134.52
130.60
129.08
128.82
128.76
128.70
127.83
127.72
125.93
123.03
122.91
117.09
116.27
77.58 CDCl3
77.16 CDCl3
76.73 CDCl3
ppm

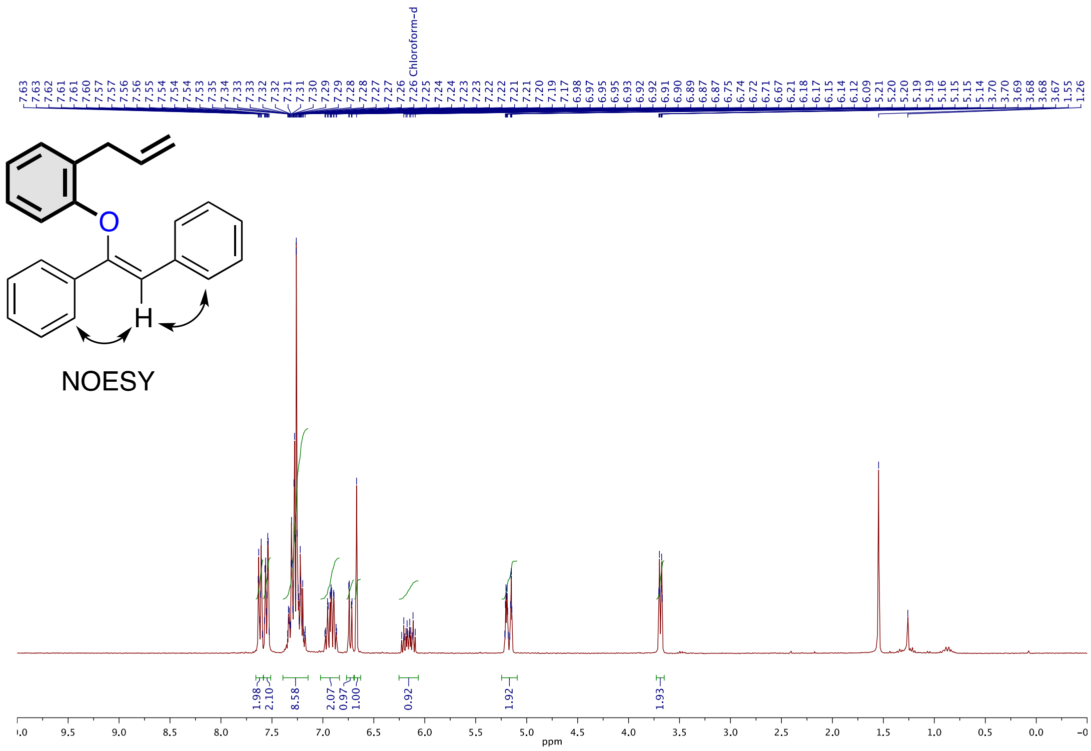

7.63 7.63 7.62 7.61 7.61 7.60 7.57 7.57 7.56 7.56 7.55 7.54 7.54 7.54 7.53 7.35 7.34 7.33 7.33 7.32 7.32 7.31 7.31 7.30 7.29 7.29 7.28 7.28 7.27 7.27 7.26 Chloroform-d 7.26 7.25 7.24 7.24 7.23 7.23 7.22 7.22 7.21 7.21 7.20 7.19 7.17 6.98 6.97 6.95 6.95 6.93 6.92 6.92 6.91 6.90 6.89 6.87 6.87 6.75 6.74 6.72 6.71 6.67 6.21 6.18 6.17 6.15 6.14 6.12 6.09 5.21 5.20 5.20 5.19 5.19 5.16 5.15 5.15 5.14 3.70 3.69 3.68 3.68 3.67 1.55 1.26
O
H
NOESY
1.98
2.10
8.58
2.07
0.97
1.00
0.92
1.92
1.93
ppm

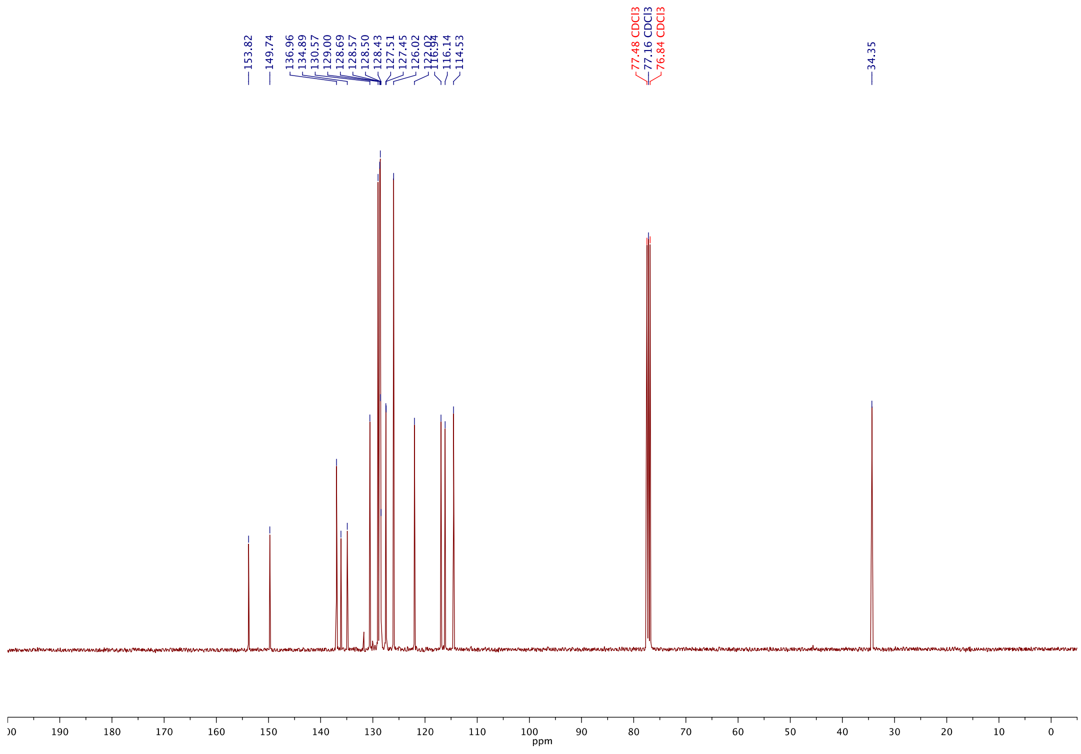

153.82
149.74
136.96 134.89 130.57 129.00 128.69 128.57 128.50 128.43 127.51 127.45 126.02 122.02 116.94 116.14 114.53
77.48 CDCl3
77.16 CDCl3
76.84 CDCl3
34.35
ppm

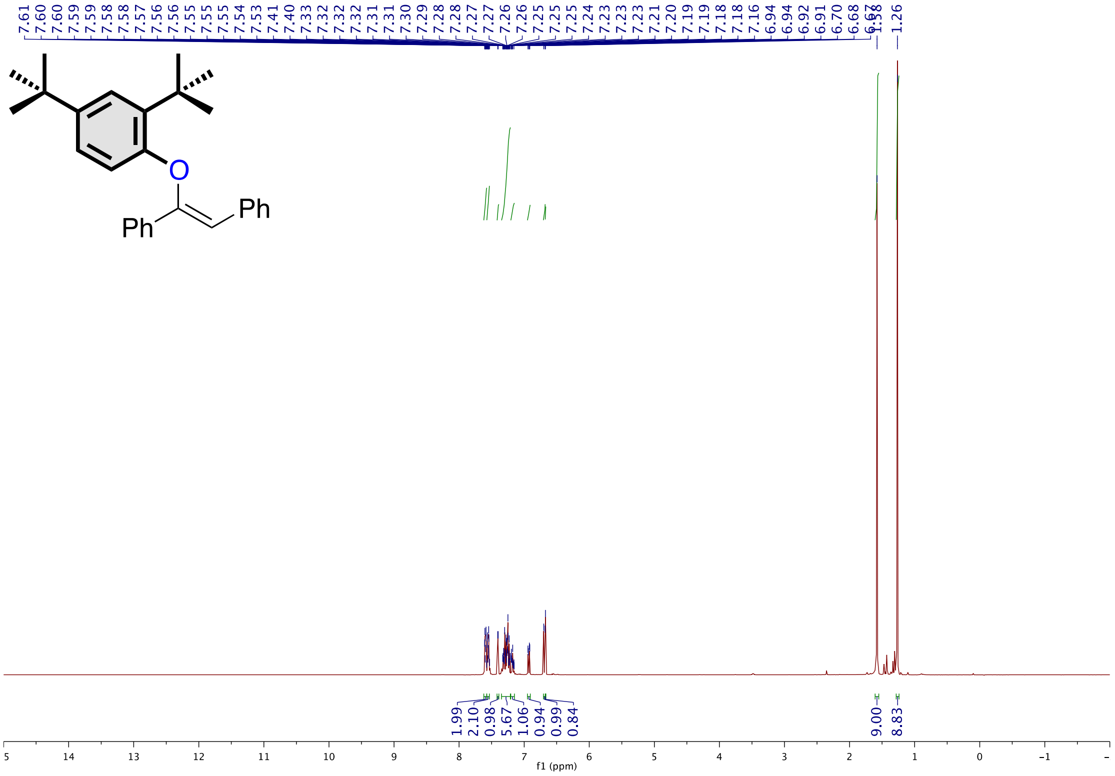
7.61
7.60
7.60
7.59
7.59
7.58
7.58
7.57
7.56
7.56
7.55
7.55
7.55
7.54
7.53
7.41
7.40
7.33
7.32
7.32
7.32
7.31
7.31
7.30
7.29
7.28
7.28
7.27
7.27
7.26
7.26
7.25
7.25
7.25
7.24
7.23
7.23
7.23
7.21
7.20
7.19
7.19
7.18
7.18
7.16
6.94
6.92
6.91
6.70
6.68
6.67
1.58
1.26
O
Ph
Ph
1.99
2.10
0.98
5.67
1.06
0.94
0.99
0.84
9.00
8.83
f1 (ppm)

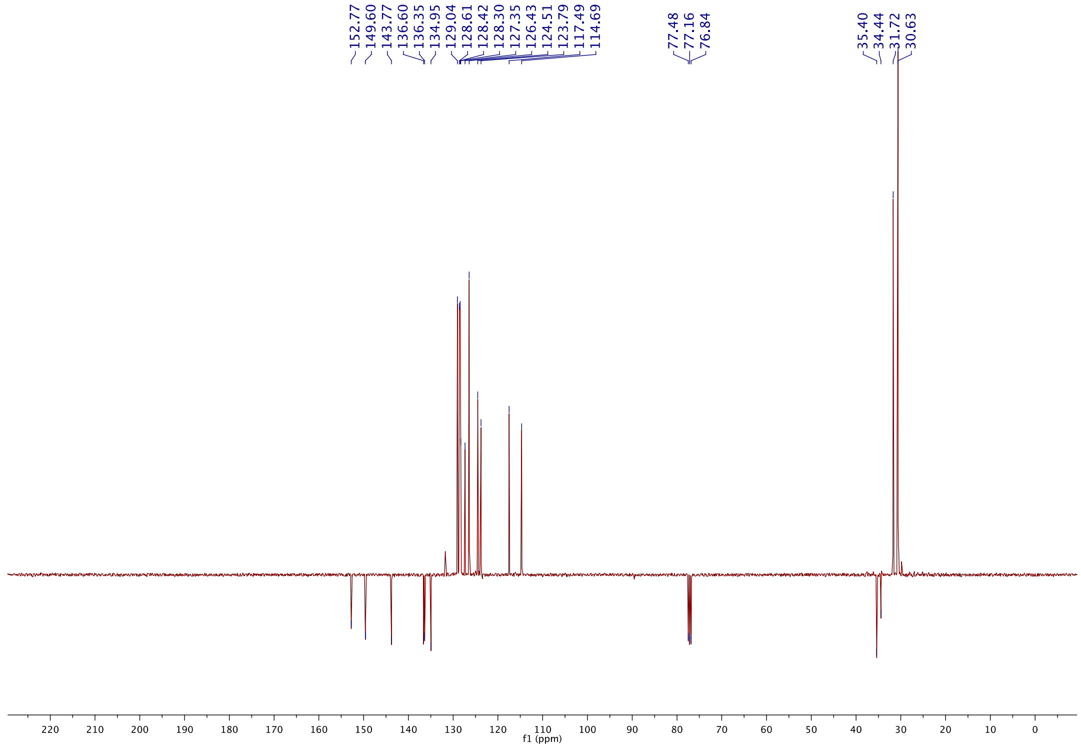
152.77
149.60
143.77
136.60
136.35
134.95
129.04
128.61
128.42
128.30
127.35
126.43
124.51
123.79
117.49
114.69
77.48
77.16
76.84
35.40
34.44
31.72
30.63
f1 (ppm)

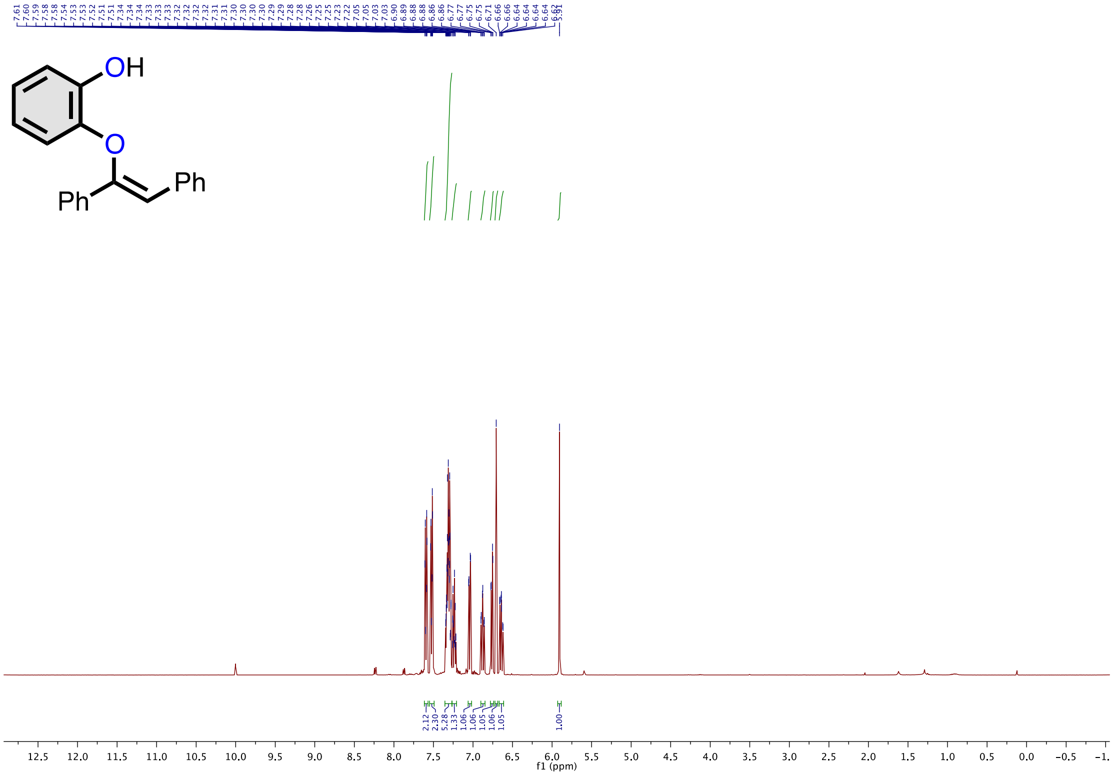
OH
O
Ph
Ph
f1 (ppm)

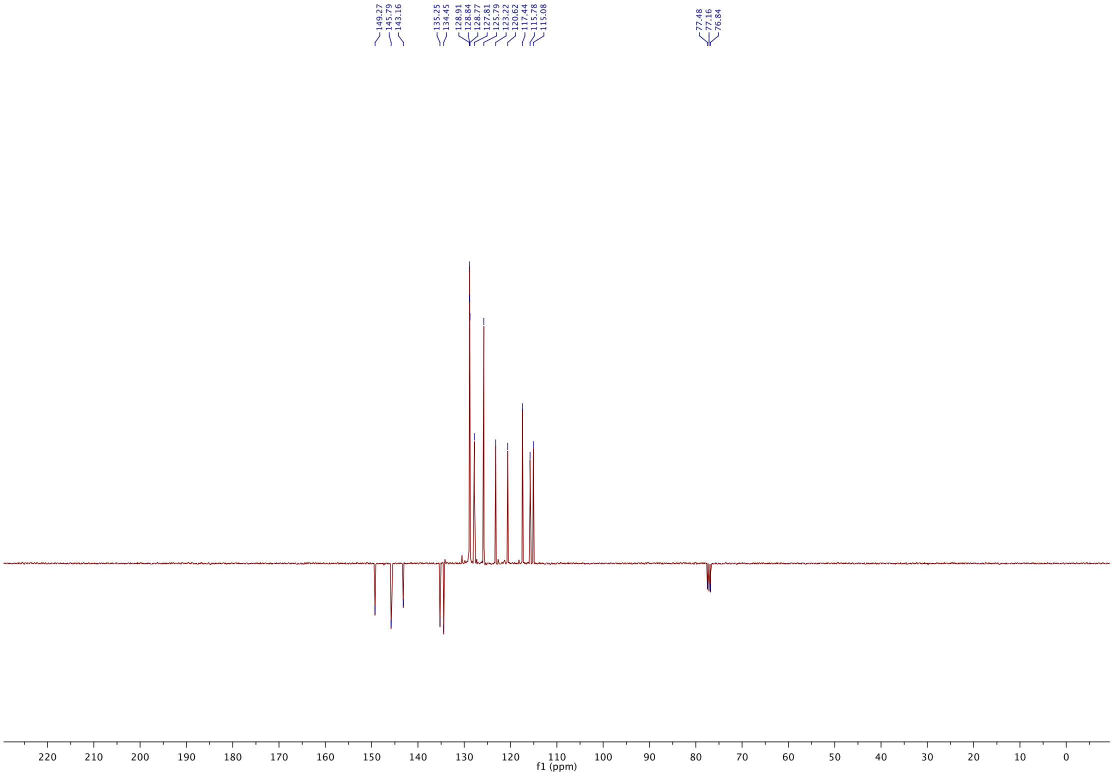
149.27
145.79
143.16
135.25
134.45
128.91
128.84
128.77
127.81
125.79
123.22
120.62
117.44
115.78
115.08
77.48
77.16
76.84
f1 (ppm)

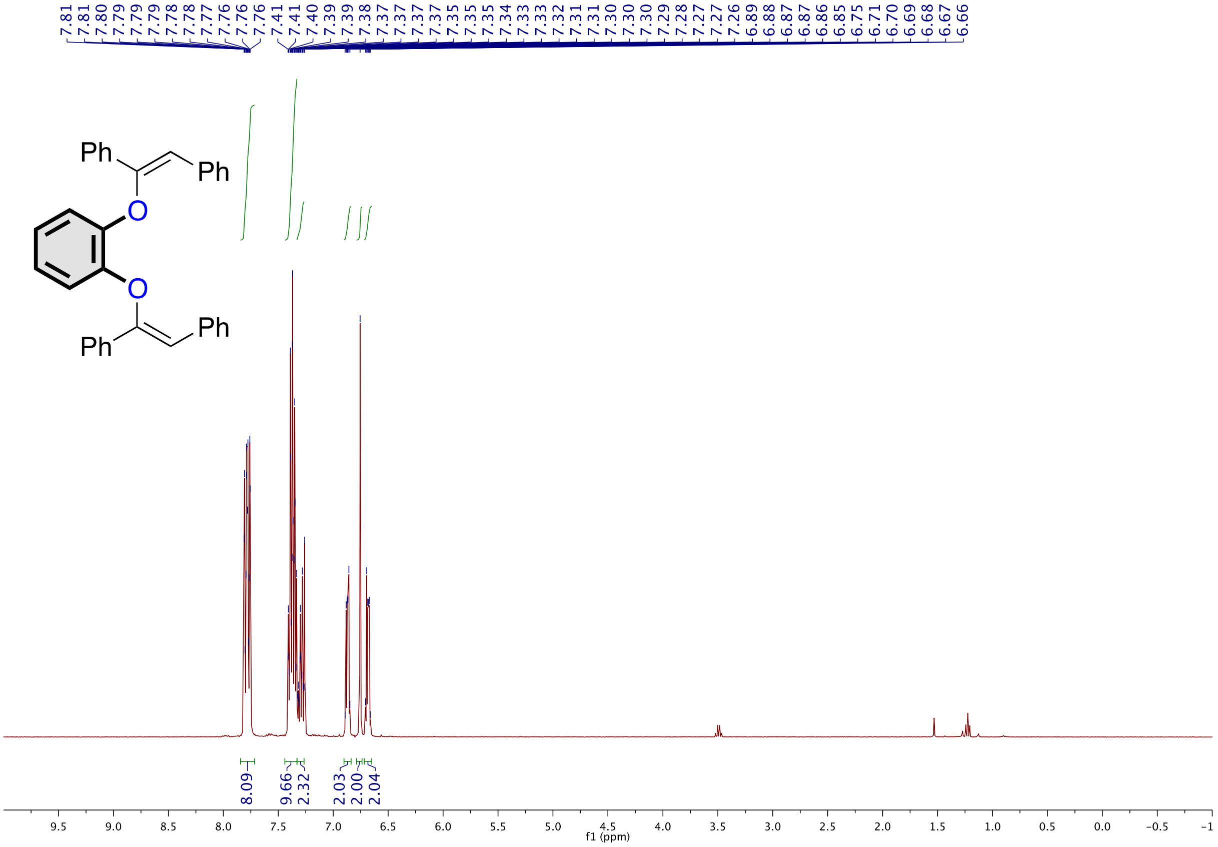

7.81 7.81 7.80 7.79 7.79 7.78 7.78 7.77 7.76 7.76 7.41 7.41 7.40 7.39 7.38 7.37 7.37 7.37 7.35 7.35 7.34 7.33 7.33 7.32 7.31 7.31 7.30 7.30 7.29 7.28 7.27 7.27 7.26 6.89 6.88 6.87 6.87 6.86 6.85 6.75 6.71 6.70 6.69 6.68 6.67 6.66
Ph
Ph
O
O
Ph
Ph
8.09
9.66
2.32
2.03
2.00
2.04
9.5 9.0 8.5 8.0 7.5 7.0 6.5 6.0 5.5 5.0 4.5 4.0 3.5 3.0 2.5 2.0 1.5 1.0 0.5 0.0 −0.5 −1
f1 (ppm)

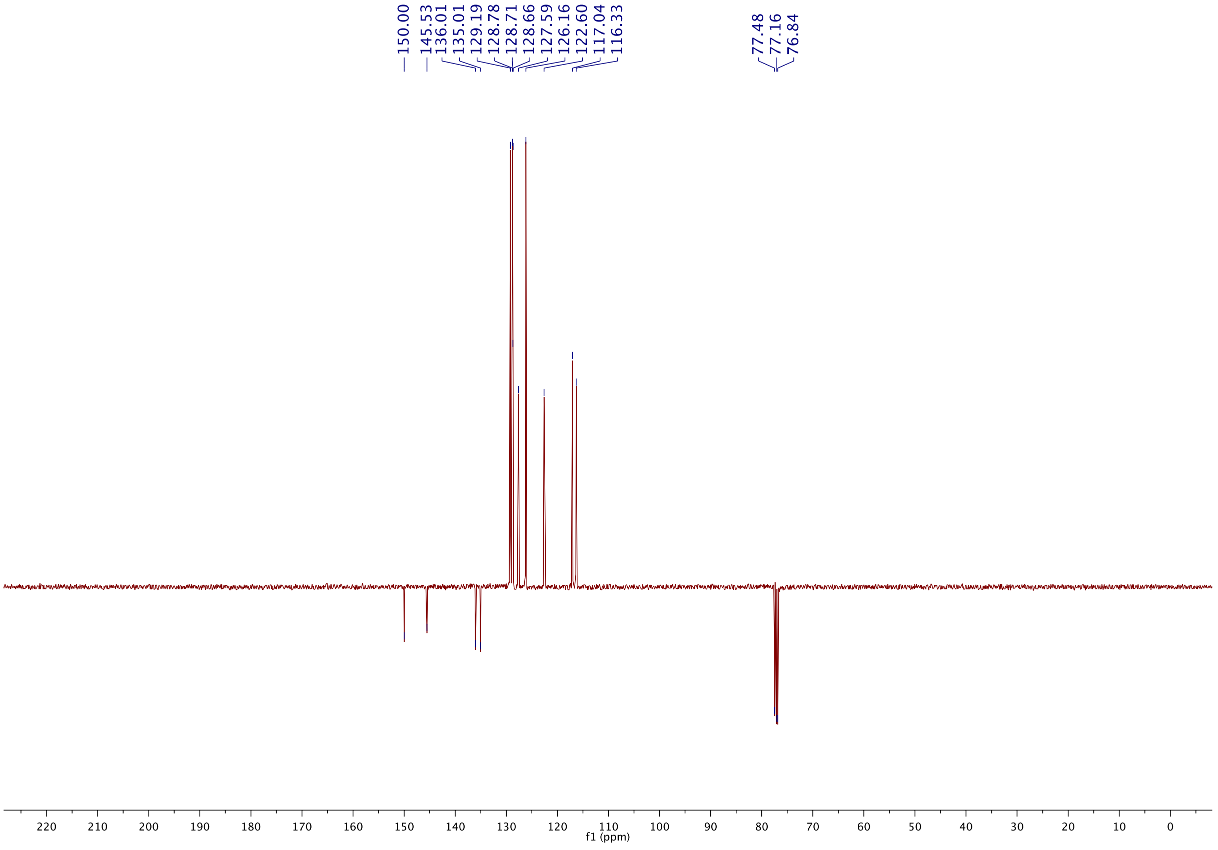

150.00 145.53 136.01 135.01 129.19 128.78 128.71 128.66 127.59 126.16 122.60 117.04 116.33
77.48 77.16 76.84
220 210 200 190 180 170 160 150 140 130 120 110 100 90 80 70 60 50 40 30 20 10 0
f1 (ppm)

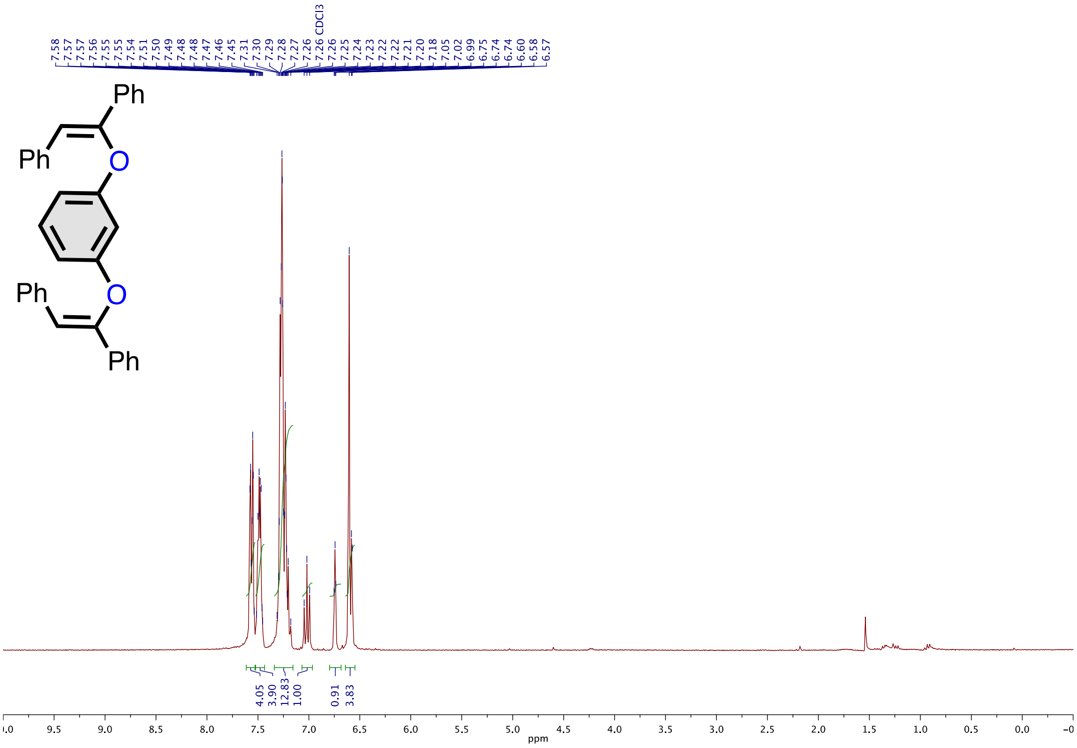
7.58
7.57
7.57
7.56
7.55
7.55
7.54
7.51
7.50
7.49
7.48
7.48
7.47
7.46
7.45
7.31
7.30
7.29
7.28
7.27
7.26
7.26 CDCl3
7.26
7.25
7.24
7.23
7.22
7.22
7.21
7.20
7.18
7.05
7.02
6.99
6.75
6.74
6.74
6.60
6.58
6.57
Ph
Ph
O
Ph
O
Ph
4.05
3.90
12.83
1.00
0.91
3.83
ppm

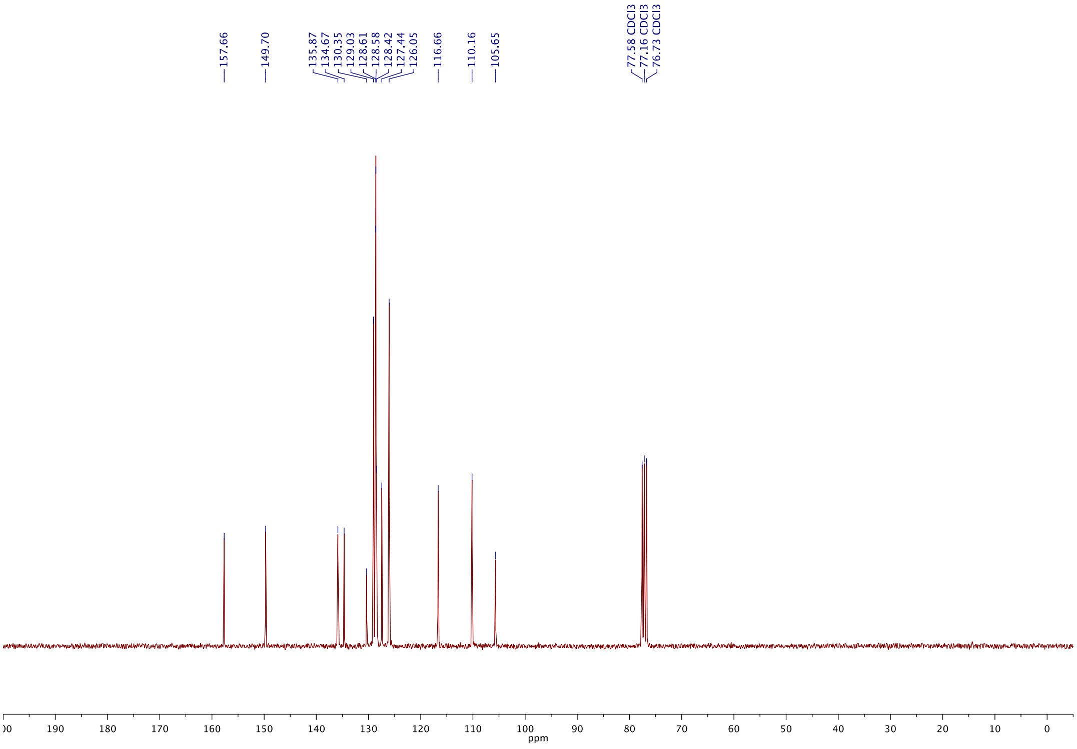
157.66
149.70
135.87
134.67
130.35
129.03
128.61
128.58
128.42
127.44
126.05
116.66
110.16
105.65
77.58 CDCl3
77.16 CDCl3
76.73 CDCl3
ppm

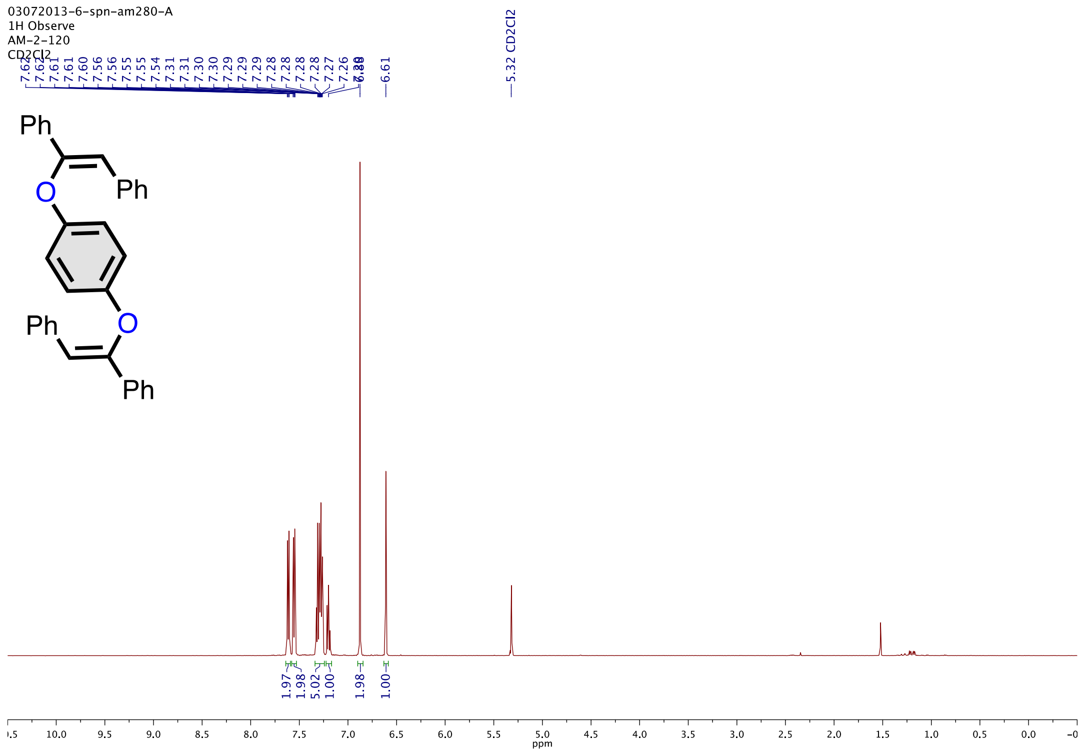

03072013-6-spn-am280-A
1H Observe
AM-2-120
CD2Cl2
7.62
7.62
7.61
7.60
7.56
7.56
7.55
7.55
7.54
7.31
7.31
7.30
7.30
7.29
7.29
7.29
7.28
7.28
7.28
7.28
7.27
7.26
6.61
5.32 CD2Cl2
Ph
O
Ph
Ph
O
Ph
1.97
1.98
5.02
1.00
1.98
1.00
10.0
9.5
9.0
8.5
8.0
7.5
7.0
6.5
6.0
5.5
5.0
4.5
4.0
3.5
3.0
2.5
2.0
1.5
1.0
0.5
0.0
ppm

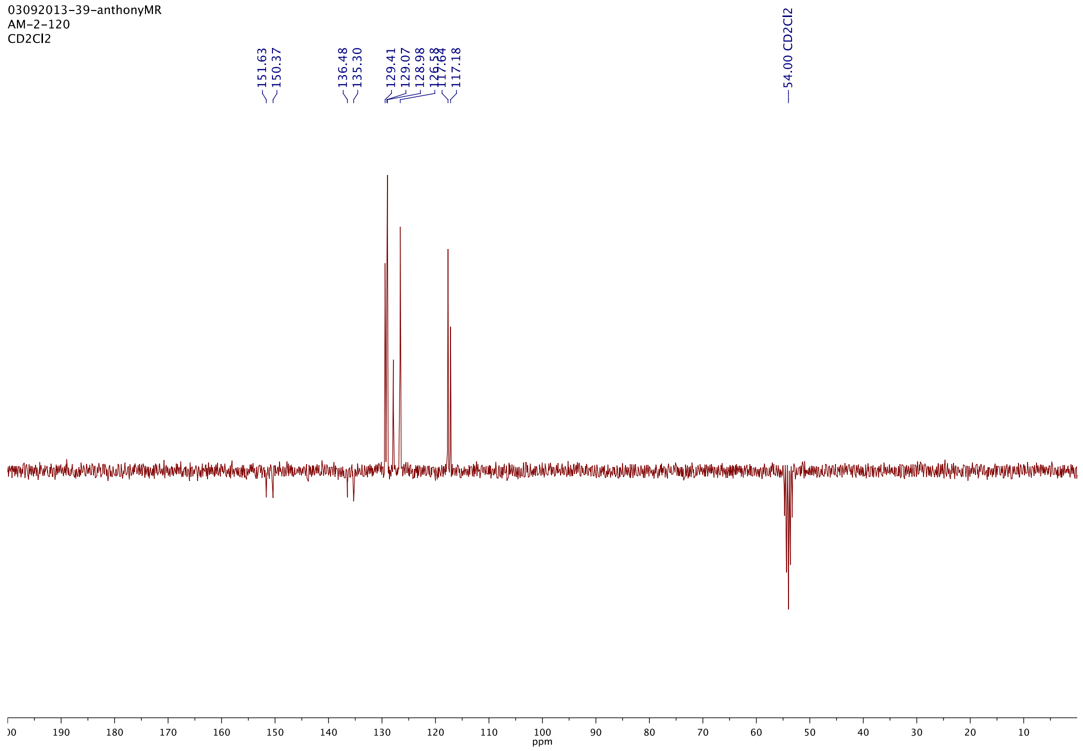

03092013-39-anthonyMR
AM-2-120
CD2Cl2
151.63
150.37
136.48
135.30
129.41
129.07
128.98
117.18
54.00 CD2Cl2
190
180
170
160
150
140
130
120
110
100
90
80
70
60
50
40
30
20
10
ppm

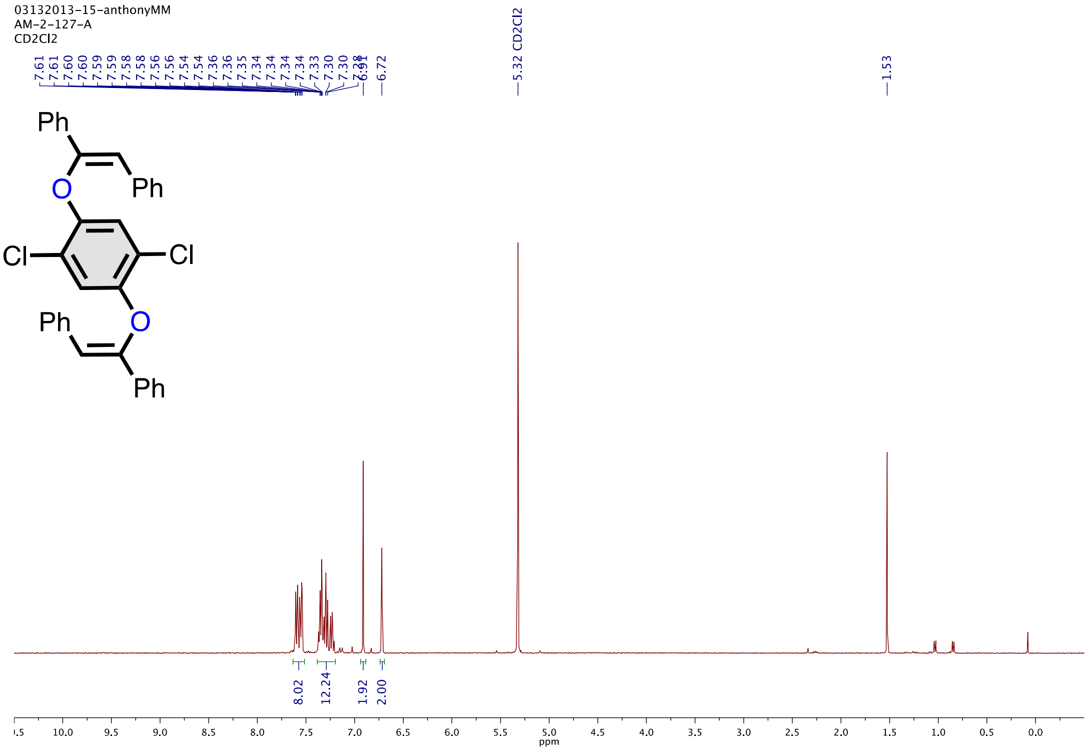
03132013-15-anthonyMM
AM-2-127-A
CD2Cl2
7.61
7.61
7.60
7.59
7.59
7.58
7.58
7.56
7.56
7.54
7.54
7.36
7.36
7.35
7.34
7.34
7.34
7.34
7.33
7.30
7.30
6.91
6.72
5.32 CD2Cl2
1.53
Ph
O
Ph
Cl
Cl
Ph
O
Ph
8.02
12.24
1.92
2.00
ppm

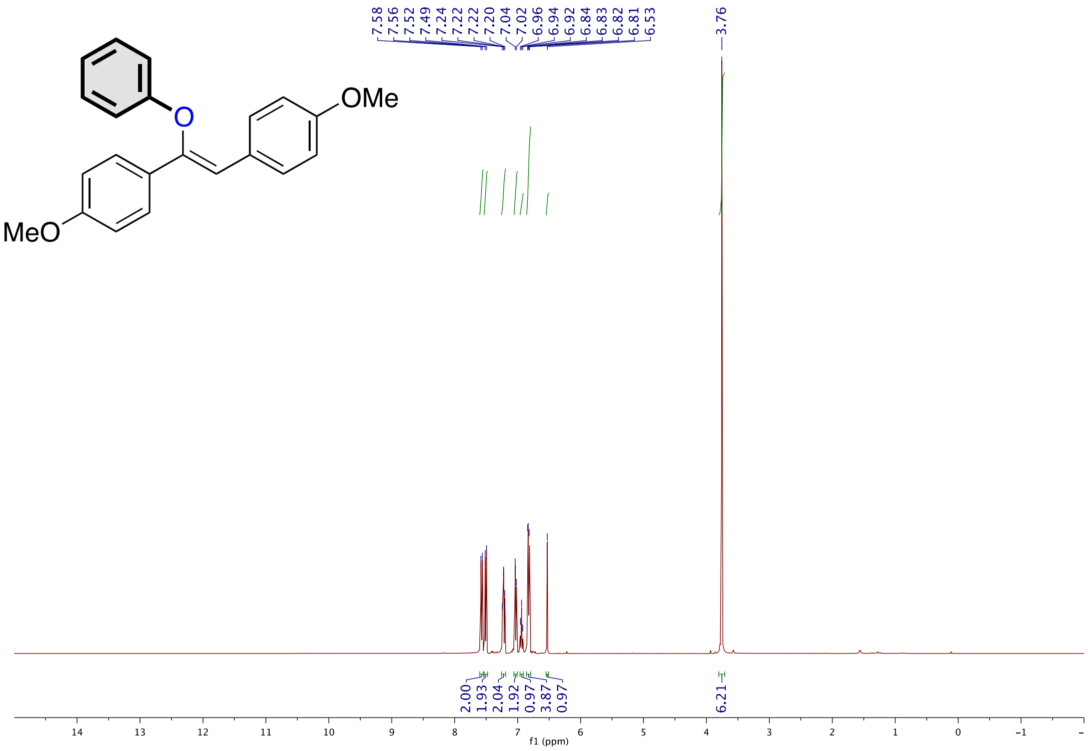
OMe
O
MeO
7.58
7.56
7.52
7.49
7.24
7.22
7.22
7.20
7.04
7.02
6.96
6.94
6.92
6.84
6.83
6.82
6.81
6.53
3.76
2.00
1.93
2.04
1.92
0.97
3.87
0.97
6.21
f1 (ppm)

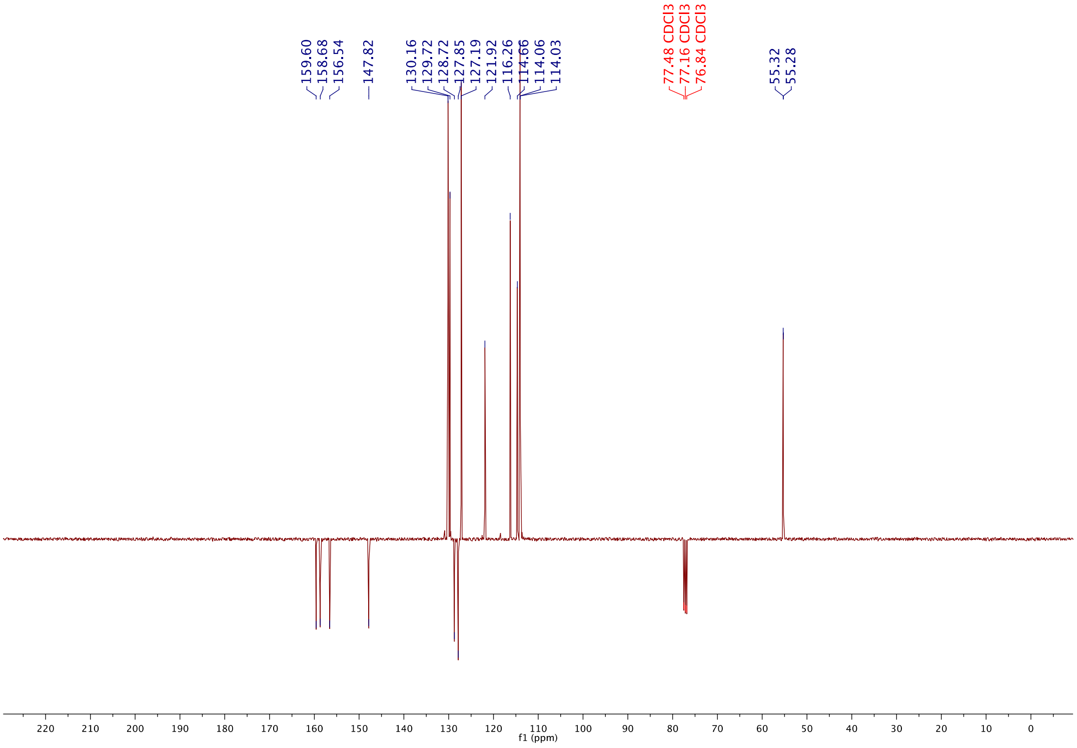
159.60
158.68
156.54
147.82
130.16
129.72
128.72
127.85
127.19
121.92
116.26
114.66
114.06
114.03
77.48 CDCl3
77.16 CDCl3
76.84 CDCl3
55.32
55.28
f1 (ppm)

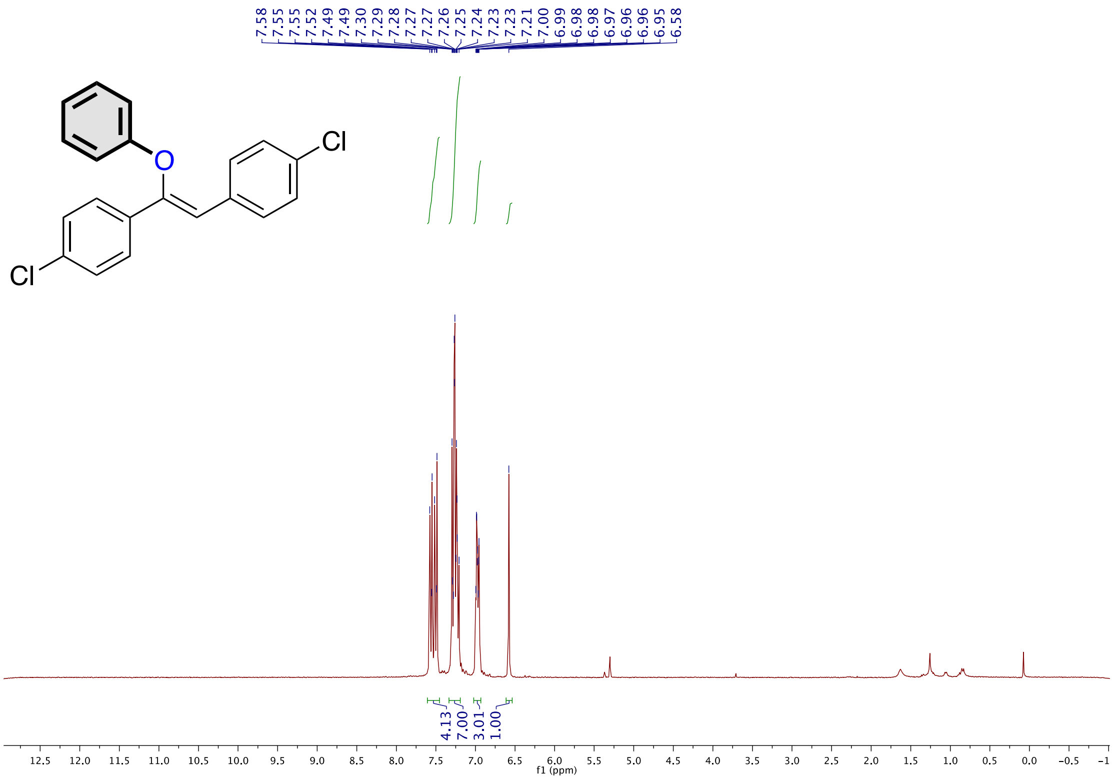
Cl
O
Cl
7.58
7.55
7.55
7.52
7.49
7.49
7.30
7.29
7.28
7.27
7.27
7.26
7.25
7.24
7.23
7.23
7.21
7.00
6.99
6.98
6.98
6.97
6.96
6.96
6.95
6.58
4.13
7.00
3.01
1.00
f1 (ppm)

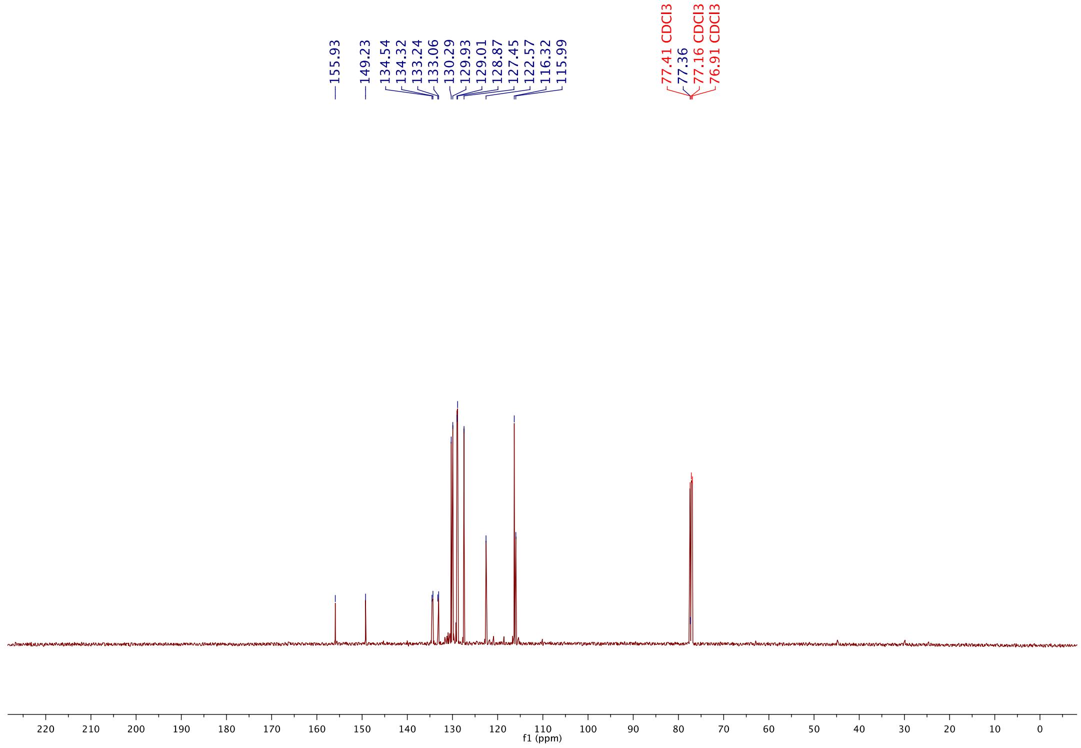
155.93
149.23
134.54
134.32
133.24
133.06
130.29
129.93
129.01
128.87
127.45
122.57
116.32
115.99
77.41 CDCl3
77.36
77.16 CDCl3
76.91 CDCl3
f1 (ppm)

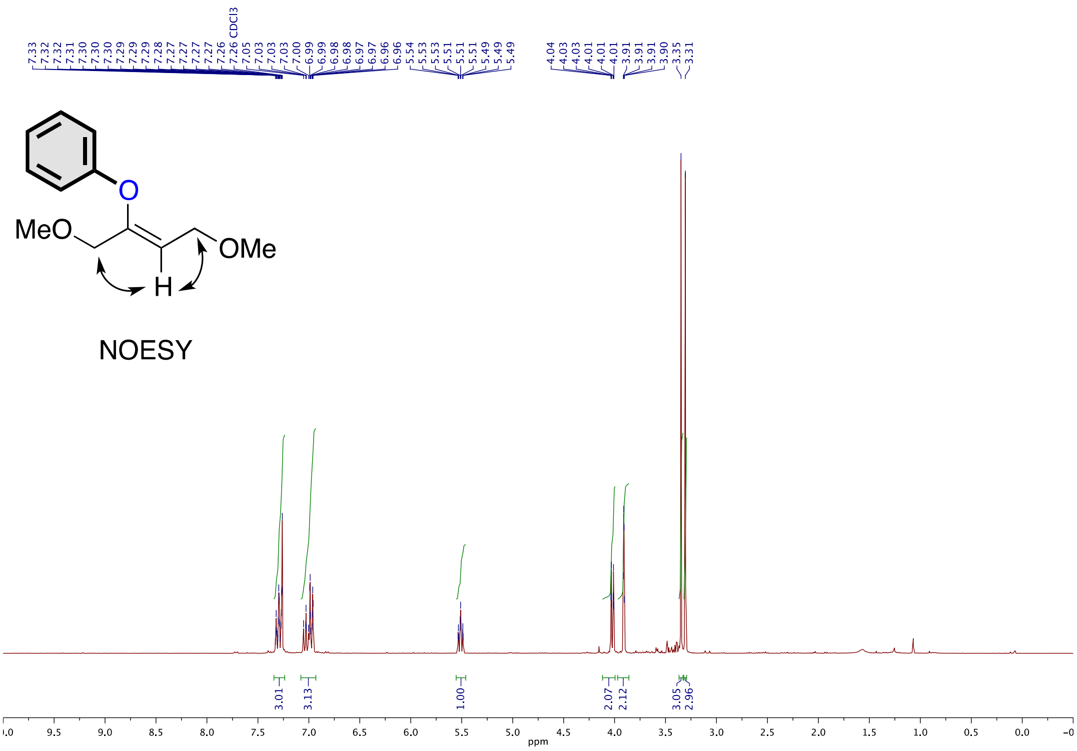
7.33
7.32
7.32
7.31
7.30
7.30
7.29
7.29
7.29
7.28
7.27
7.27
7.27
7.27
7.26 CDCl3
7.05
7.03
7.03
7.03
7.00
6.99
6.99
6.98
6.98
6.97
6.97
6.96
6.96
5.54
5.53
5.53
5.51
5.51
5.51
5.49
5.49
5.49
4.04
4.03
4.03
4.01
4.01
4.01
3.91
3.91
3.91
3.90
3.35
3.31
MeO
O
OMe
H
NOESY
3.01
3.13
1.00
2.07
2.12
3.05
2.96
ppm

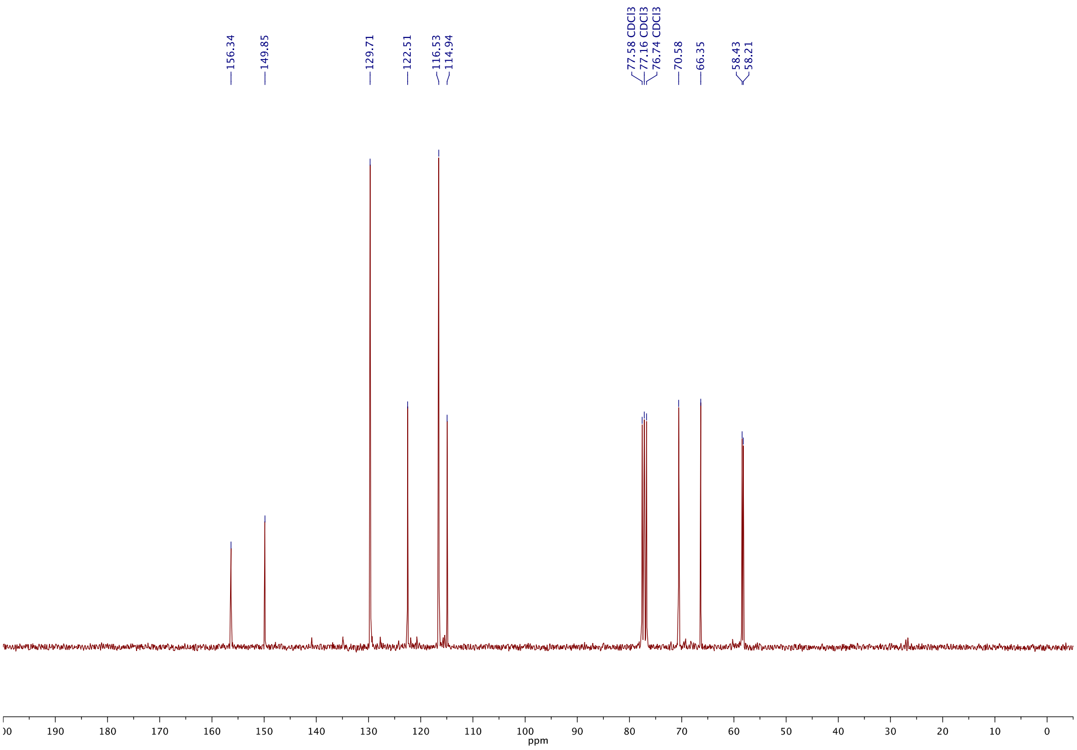
156.34
149.85
129.71
122.51
116.53
114.94
77.58 CDCl3
77.16 CDCl3
76.74 CDCl3
70.58
66.35
58.43
58.21
ppm

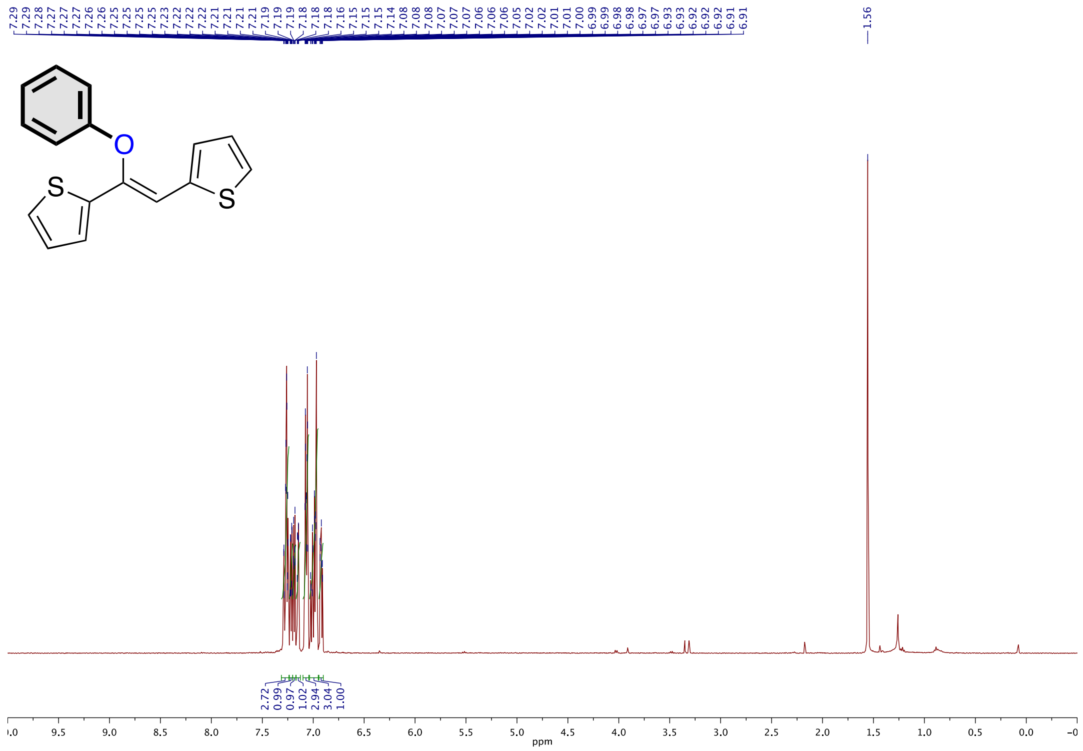

7.29 7.29 7.28 7.27 7.27 7.27 7.26 7.26 7.25 7.25 7.25 7.23 7.22 7.22 7.22 7.21 7.21 7.21 7.21 7.19 7.19 7.18 7.18 7.18 7.16 7.15 7.15 7.15 7.14 7.08 7.08 7.08 7.07 7.07 7.07 7.06 7.06 7.05 7.02 7.02 7.01 7.01 7.00 6.99 6.99 6.98 6.98 6.97 6.97 6.93 6.93 6.92 6.92 6.91 6.91
1.56
O
S
S
2.72 0.99 0.97 1.02 2.94 3.04 1.00
ppm

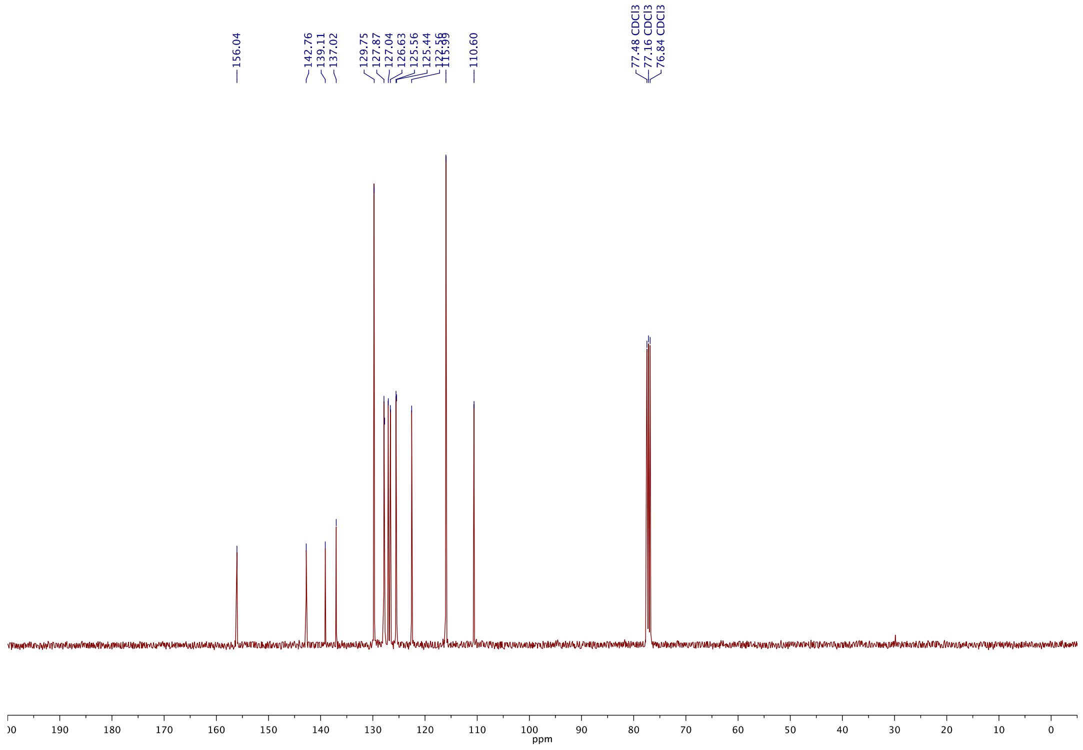

156.04
142.76 139.11 137.02
129.75 127.87 127.04 126.63 125.56 125.44 122.56 115.99
110.60
77.48 CDCl3
77.16 CDCl3
76.84 CDCl3
ppm

05312013-4-anthonyMR.10.fid
AM-2-190
CDCl3

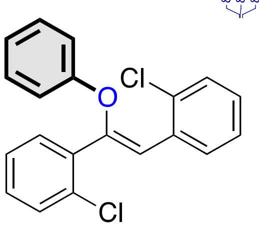

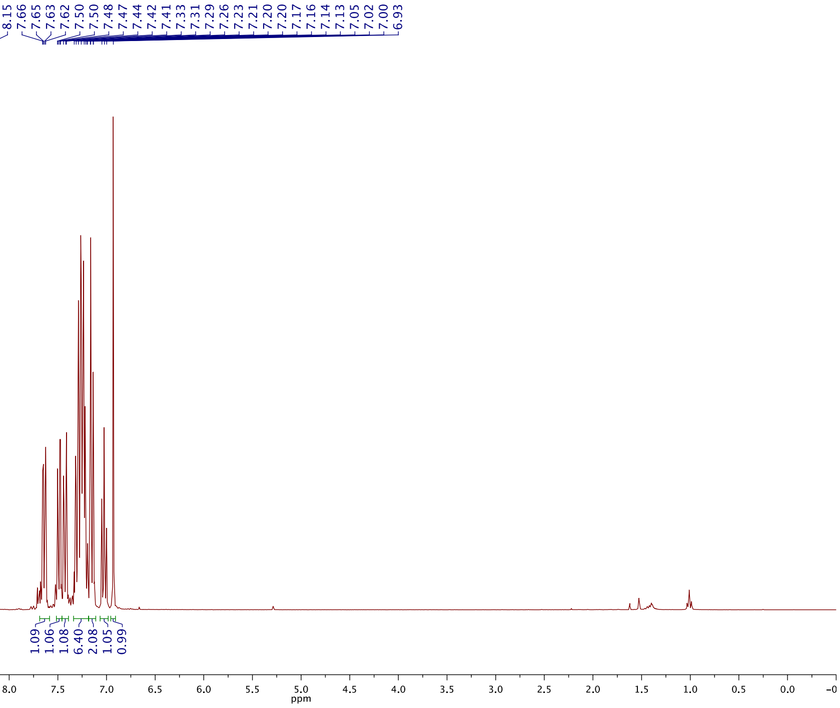

06012013-25-anthonyMI.10.fid
AM-2-190
CDCl3

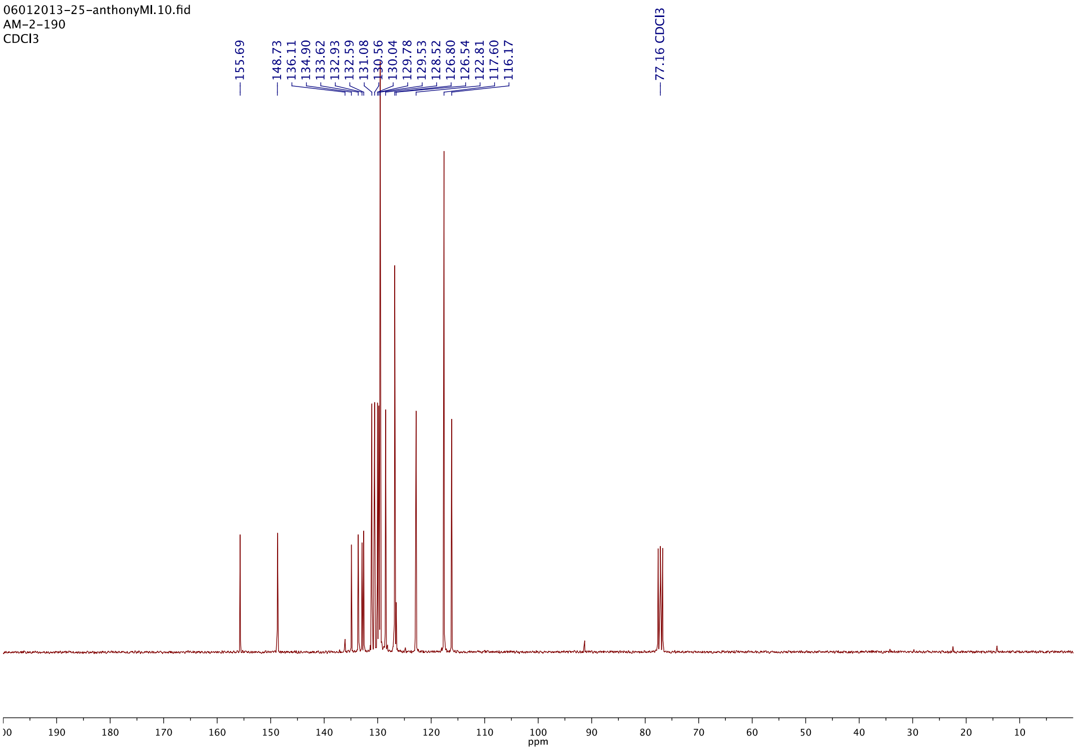

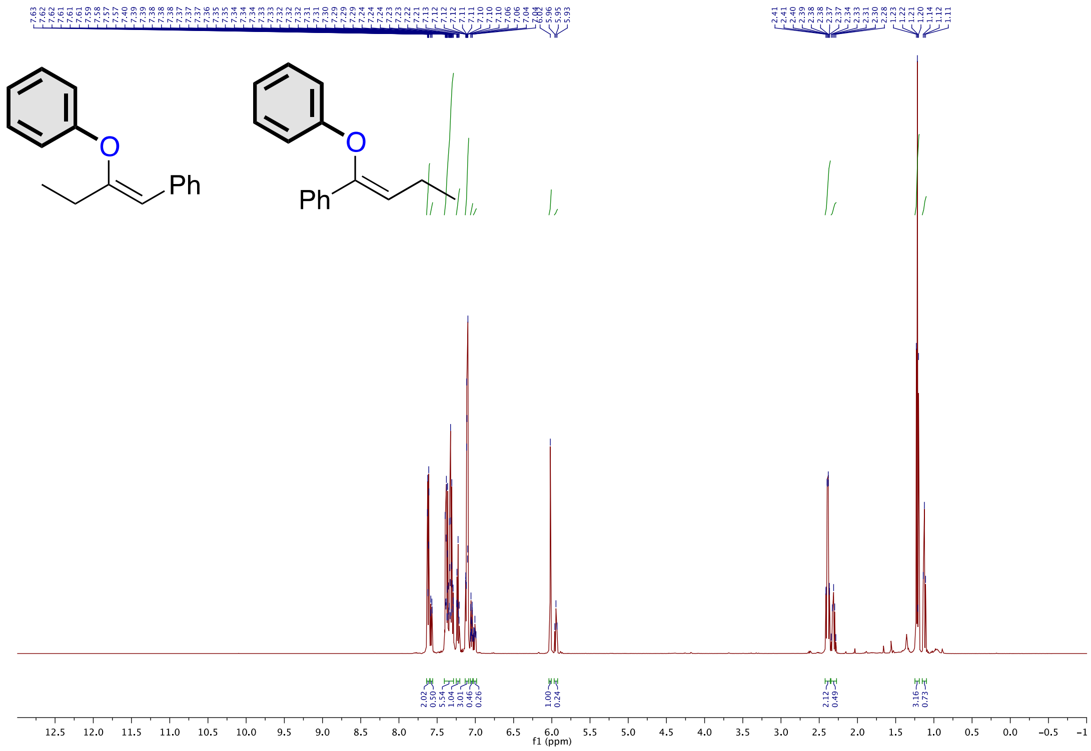
O
Ph
O
Ph
f1 (ppm)

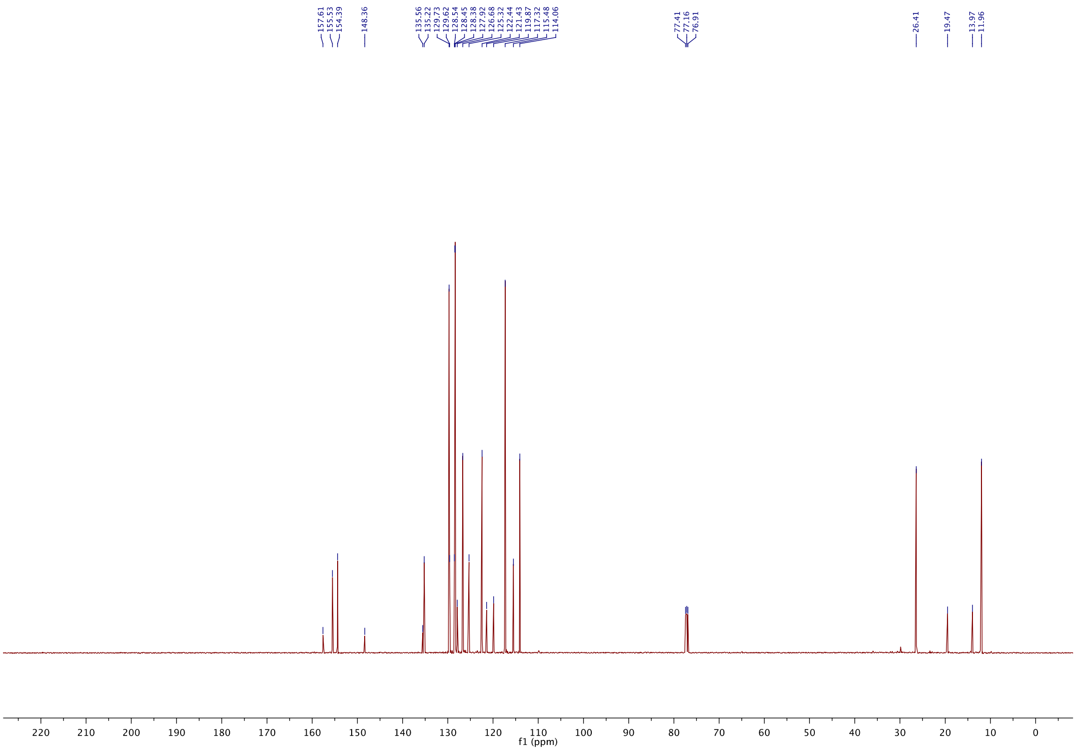
f1 (ppm)

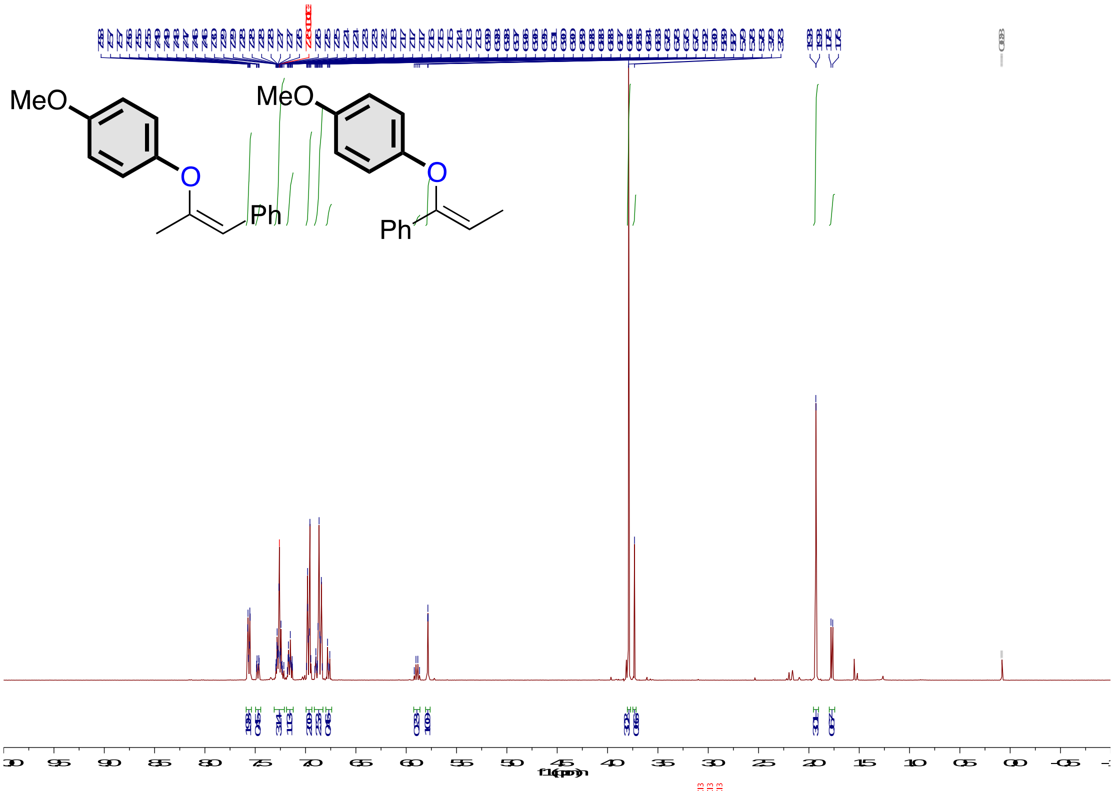
MeO
O
Ph
MeO
O
Ph
f1 (ppm)

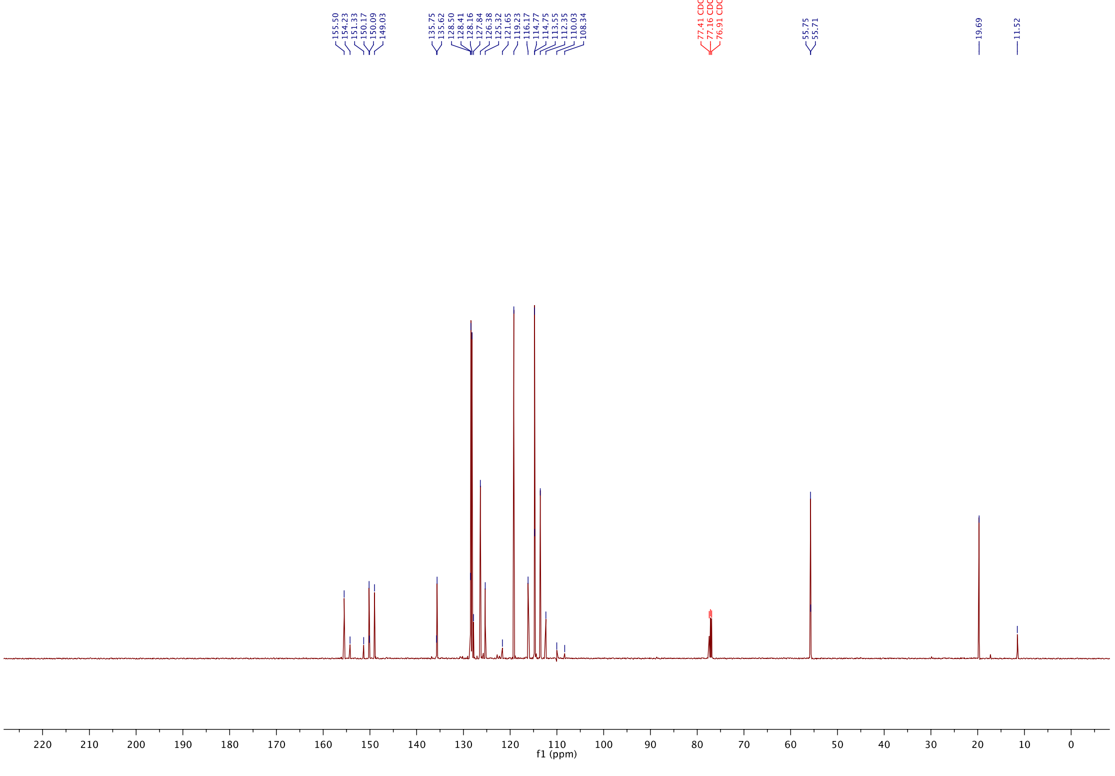
155.50
154.23
151.33
150.17
150.09
149.03
135.75
135.62
128.50
128.41
128.16
127.84
126.38
125.32
121.65
119.23
116.17
114.77
114.75
113.55
112.35
110.03
108.34
77.41 CDCl3
77.16 CDCl3
76.91 CDCl3
55.75
55.71
19.69
11.52
f1 (ppm)

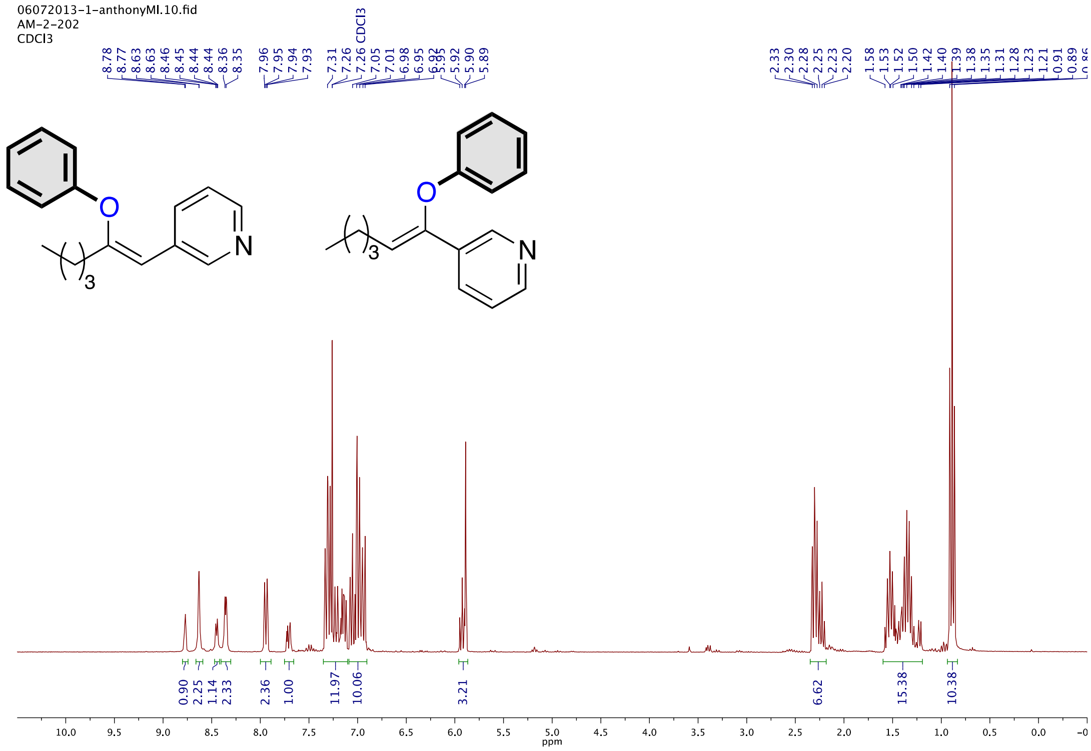
06072013-1-anthonyMI.10.fid
AM-2-202
CDCl3
O
N
3
ppm

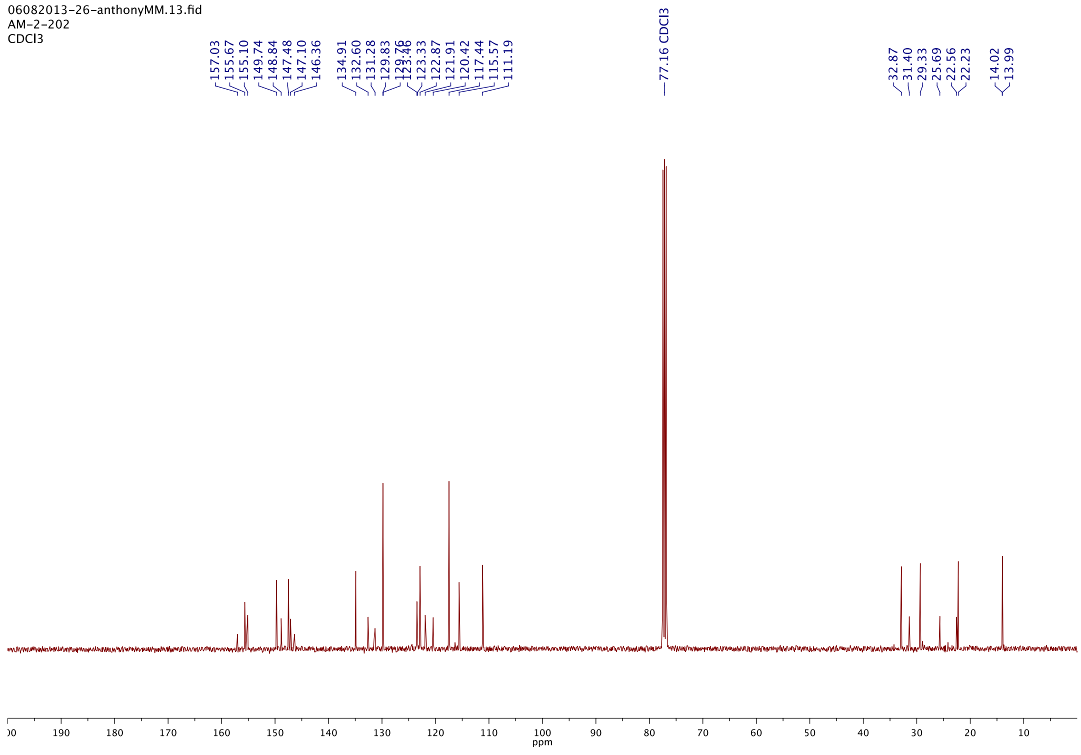
06082013-26-anthonyMM.13.fid
AM-2-202
CDCl3
77.16 CDCl3
ppm

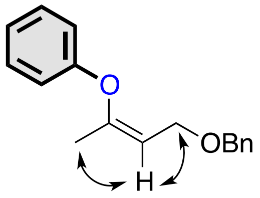
O
OBn
H

NOESY

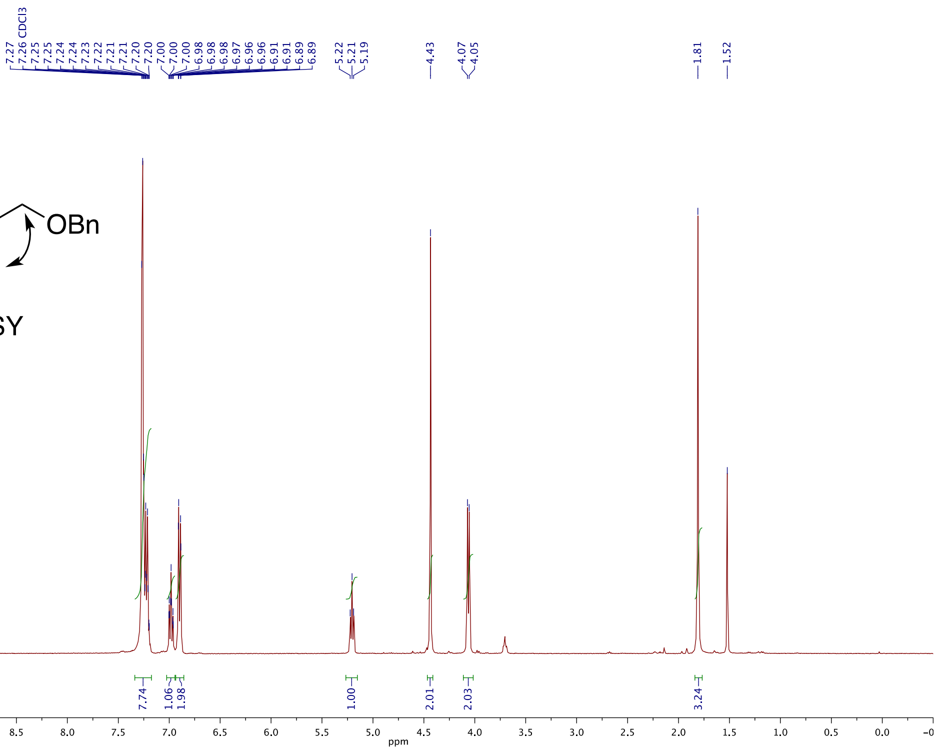
7.27 CDCl3
7.26 CDCl3
7.25
7.25
7.24
7.24
7.23
7.22
7.21
7.21
7.20
7.20
7.00
7.00
7.00
6.98
6.98
6.98
6.97
6.96
6.96
6.91
6.91
6.89
6.89
5.22
5.21
5.19
4.43
4.07
4.05
1.81
1.52
7.74
1.06
1.98
1.00
2.01
2.03
3.24
ppm

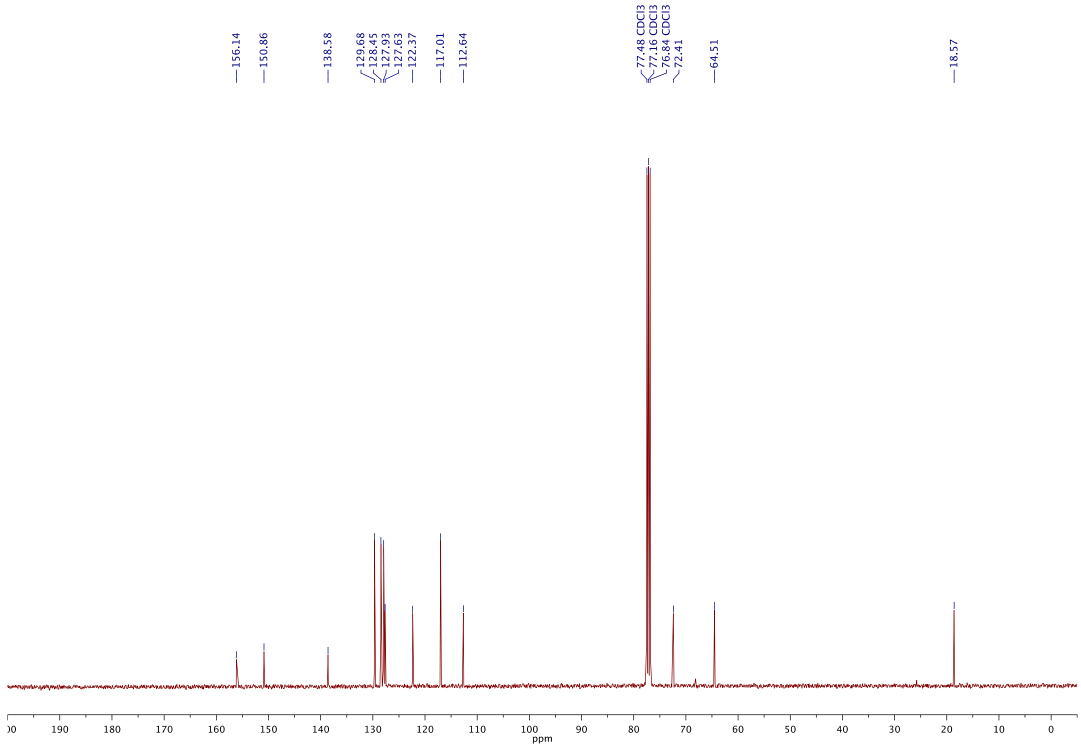
156.14
150.86
138.58
129.68
128.45
127.93
127.63
122.37
117.01
112.64
77.48 CDCl3
77.16 CDCl3
76.84 CDCl3
72.41
64.51
18.57
ppm

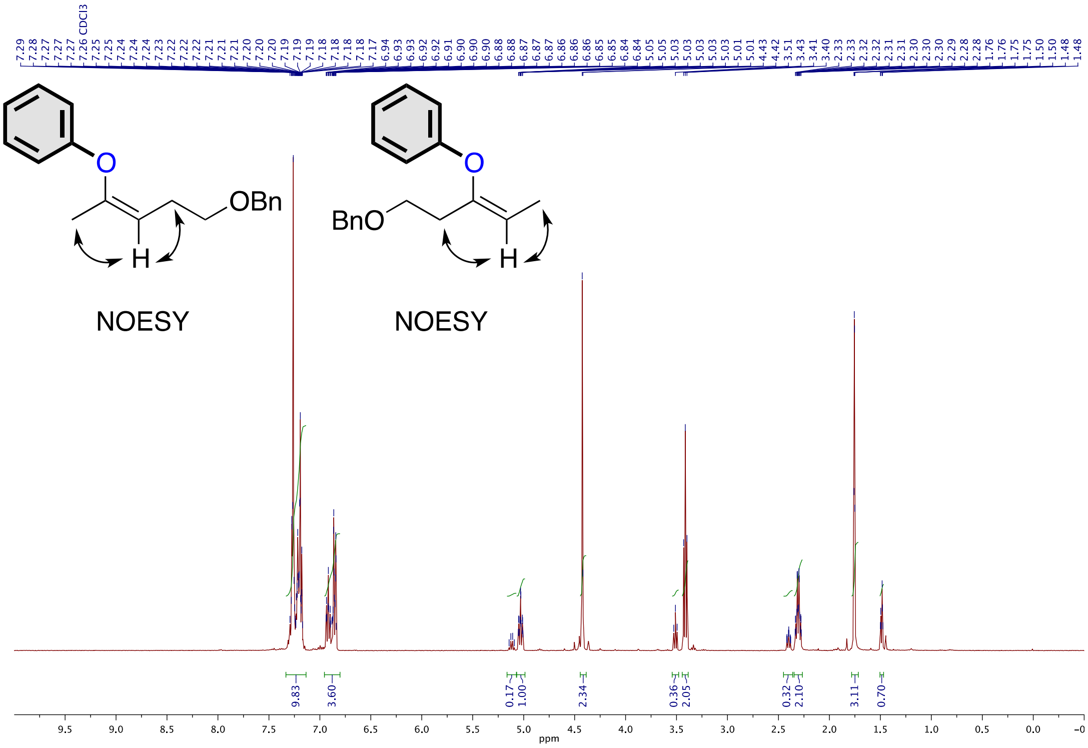

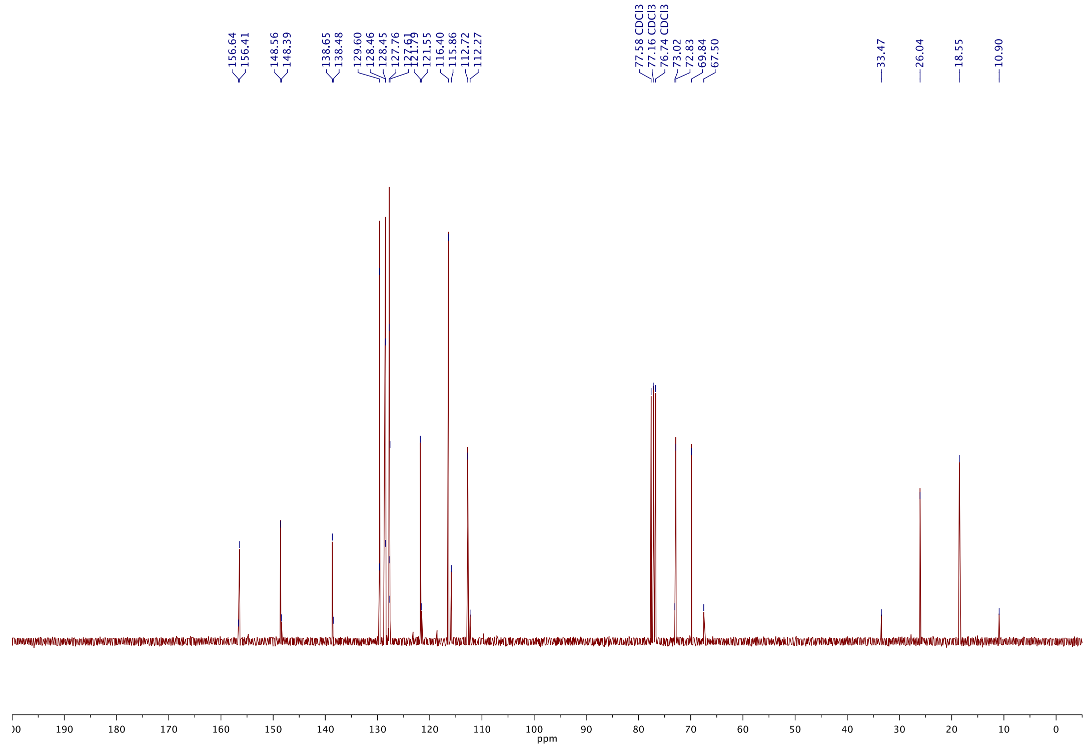

[1] a) R. S. Ramón, S. Gaillard, A. Poater, L. Cavallo, A. M. Z. Slawin, S. P. Nolan, *Chem. Eur. J.* **2011**, *17*, 1238-1246; b) A. Gómez-Suárez, Y. Oonishi, S. Meiries, S. P. Nolan, *Organometallics* **2013**, *32*, 1106-1111.

[2] Y. Oonishi, A. Gomez-Suarez, A. R. Martin, S. P. Nolan, *Angew Chem Int Ed* **2013**, *52*, 9767-9771.
[3] Y. Oonishi, A. Gomez-Suarez, A. R. Martin, Y. Makida, A. M. Slawin, S. P. Nolan, *Chem. Eur. J.* **2014**, *20*, 13507-13510.
[4] M. R. Kuram, M. Bhanuchandra, A. K. Sahoo, *J. Org. Chem.* **2010**, *75*, 2247-2258.
[5] H. Hiranuma, S. I. Miller, *J. Org. Chem.* **1982**, *47*, 5083-5088.
[6] P. Chuentragool, K. Vongnam, P. Rashatasakhon, M. Sukwattanasinitt, S. Wacharasindhu, *Tetrahedron* **2011**, *67*, 8177-8182.
[7] K. Semba, T. Fujihara, J. Terao, Y. Tsuji, *Chem. Eur. J.* **2012**, *18*, 4179-4184.
[8] F. Alonso, I. Osante, M. Yus, *Tetrahedron* **2007**, *63*, 93-102.
